# Light-Activated RPE65 Inhibitors Enable On-Demand Visual Cycle Control

**DOI:** 10.1021/jacs.6c02962

**Published:** 2026-05-19

**Authors:** Marco Bassetto, Bowen Li, Xiuyuan Chen, Jianye Zhang, Yulun Hu, Jordan Zaluski, Lauren M. Brumit, Preston M Willis, Felix Grun, Krzysztof Palczewski, Philip D. Kiser, Gregory P. Tochtrop

**Affiliations:** † Department of Physiology and Biophysics, 8788 University of California, Irvine, Irvine, California 92697, United States; ‡ Gavin Herbert Eye Institute-Robert M. Brunson Center for Translational Vision Research, Department of Ophthalmology and Visual Sciences, 8788 University of California, Irvine, Irvine, California 92697, United States; § Research Service, VA Long Beach Healthcare System, Long Beach, California 90822, United States; ∥ Department of Chemistry, College of Arts and Sciences, 2546 Case Western Reserve University, Cleveland, Ohio 44106, United States; ⊥ Department of Chemistry, 8788 University of California, Irvine, Irvine, California 92697, United States; # Department of Molecular Biology and Biochemistry, 8788 University of California, Irvine, Irvine, California 92697, United States; ∇ Department of Clinical Pharmacy Practice, School of Pharmacy and Pharmaceutical Sciences, 8788 University of California, Irvine, Irvine, California 92697, United States

## Abstract

Light initiates visual perception, but it also exacerbates a subset of blinding diseases in which visual (retinoid) cycle metabolic intermediates contribute to pathophysiology. Small molecule visual cycle modulators (VCMs) have demonstrated efficacy in preclinical models, but have been limited clinically by chronic, indiscriminate visual cycle suppression leading to side effects including night blindness in human subjects. Here, we demonstrate VCMs that are activated via a *Z*→*E* photoisomerization of an azobenzene-containing VCM by visible light within the eye. One such VCM photoswitch, **(** ***Z*** **)-9**, is a weak inhibitor of the visual cycle isomerohydrolase, RPE65, that affords potent, rapid, and on-demand inhibition when photoisomerized to the *E*-configuration by visible light. **(** ***E*** **)-9** protects the retina from visual cycle toxicity and, after oral administration, shows a shorter pharmacodynamic duration than emixustat as measured by electroretinography. These results establish posterior-segment photopharmacology and outline a blueprint for light-activated therapies that mitigate daytime toxicity while sparing night vision.

## Graphical abstract

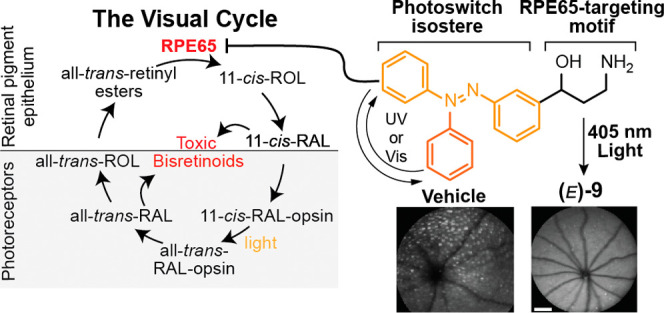

## Introduction

Light impinging on the retina initiates visual perception, but this light can also drive the progression of a subset of retinal diseases.^1^ Therefore, the eye is an ideal testbed for small molecules whose action can be activated by visible light, affording on-demand effect for retinopathy treatment or prevention. Integration of photoswitchable chemical motifs that undergo light-driven interconversion between isomeric states into drug scaffolds has enabled precise spatiotemporal control of drug action.^2^ This photopharmacologic strategy is well exemplified by K^+^ channel blockers that successfully revived light-evoked responses in retinas with degenerated photoreceptors via the isomerization of an azobenzene photoswitch triggered by light.^3^

The visual cycle is a metabolic pathway that regenerates the 11-*cis*-retinal (11-*cis*-RAL) chromophore for photoreceptor opsins.^4^ Dry age-related macular degeneration (AMD) and Stargardt disease type 1 (STGD1) are retinal diseases in which light-driven visual-cycle activity contributes to disease progression by inducing the formation of RAL-derived byproducts such as bisretinoids (Figure 1A).^1,4^ This link between visual cycle activity and retinal degeneration motivated efforts to develop visual cycle modulators (VCMs) as a therapeutic strategy. Emixustat, an orally available inhibitor of the RPE65 isomerohydrolase,^5,6^ entered clinical trials for AMD,^7^ STGD1,^8^ and diabetic retinopathy^9^ but produced dose-limiting side effects due to chronic visual cycle blockade and consequent adverse effects on rod-mediated vision, thereby limiting efficacy.^1^ The metabolic burden imposed on the visual cycle is not constant during the day compared to the night.^10^ Maximally in the photopic regime (bright daylight; > 10 cd·s/m^2^), the visual cycle metabolic flux rises to meet chromophore demand due to the elevated rate of 11-*cis* to all-*trans* photoisomerization, whereas it is minimal in the scotopic regime (dim light; 0.01 < cd·s/m^2^).^11^ We therefore reasoned that a light-activated emixustat photoswitch could curb photopic bis-retinoid production in photopic and mesopic conditions, while sparing scotopic vision.

**Figure 1:**
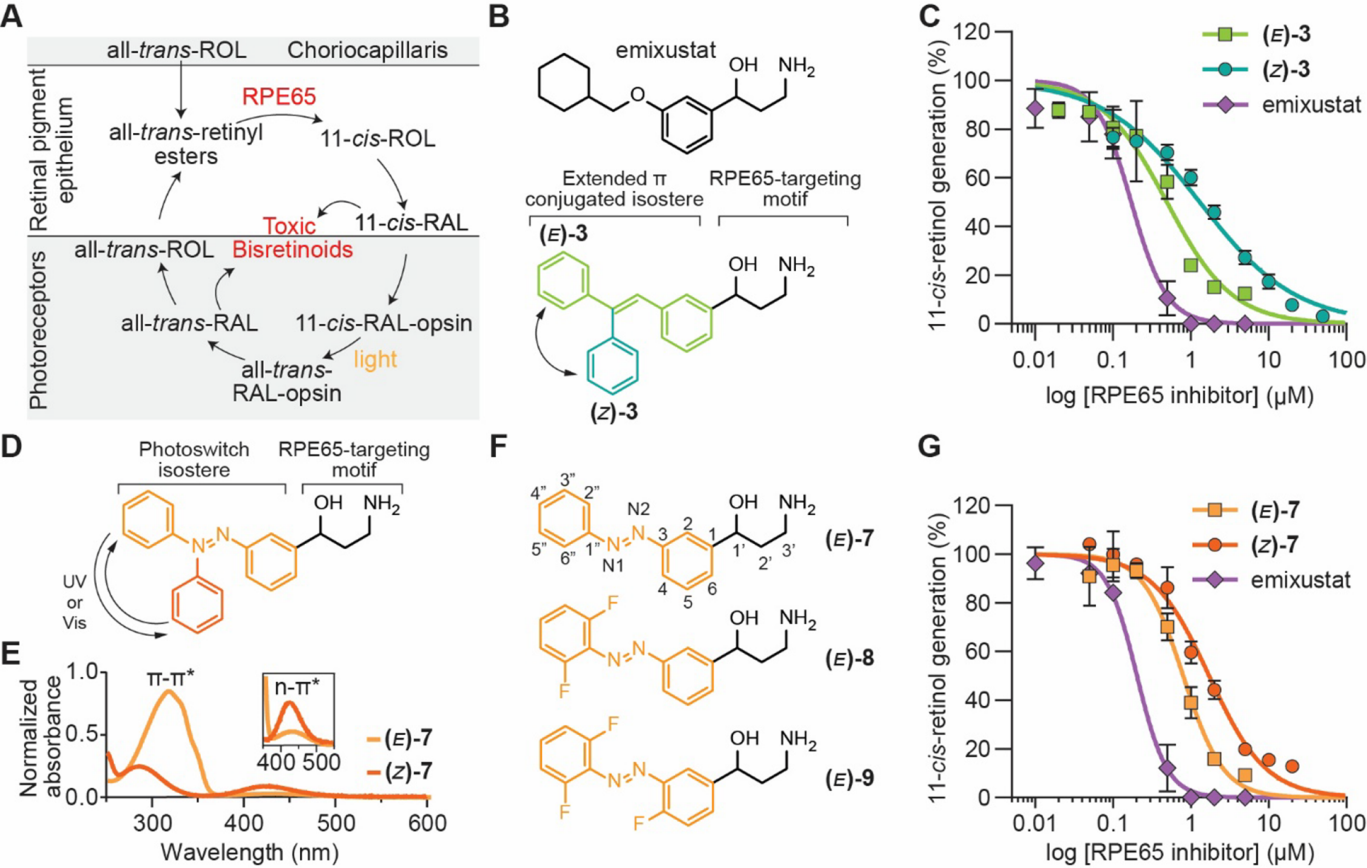
Geometric isomers of stilbene and azobenzene emixustat derivatives have different potencies toward RPE65. (A) Illustration of the visual cycle. (B) Chemical structures of the RPE65 inhibitor emixustat and its stilbene analog (3) in the *E* and *Z* configurations. (C) Dose–response curves obtained from the in vitro RPE65 activity assay showing the half-maximal inhibition concentration (IC_50_) of emixustat (purple, 0.203 ± 0.027 μM), purified **(** ***E*** **)-3** (light green, 0.494 ± 0.096 μM), and purified **(** ***Z*** **)-3** (dark green, 1.306 ± 0.308 μM). Data represent mean ± SD, *n* = 3 independent experiments. IC_50_ values were obtained by fitting the data as described in the methods. (D) Chemical structure of the nonsubstituted emixustat azolog (7) highlighting its two possible geometric isomers and their interconversion with UV or visible light (black arrows). (E) UV–vis spectra of pure **(** ***E*** **)-7** (light orange) and **(** ***Z*** **)-7** (dark orange) highlighting their π–π* and n−π* absorption bands. The n−π* absorption bands are magnified in the inset. (F) Chemical structures of synthesized emixustat azologs. (G) Dose–response curves obtained from in vitro RPE65 activity assay showing the IC_50_ of emixustat (purple, 0.203 ± 0.027 μM), **(** ***E*** **)-7** (bright orange, 0.796 ± 0.102 μM), **(** ***Z*** **)-7** (dark orange, 1.713 ± 0.265 μM). Data represent means ± SD, *n* = 3 independent experiments. IC_50_ values were obtained by fitting the data as described in the methods.

Here, we synthesized new RPE65-targetted VCMs capable of photoswitching in response to visible light by installing an *ortho*-fluorinated azobenzene photoswitch on the RPE65 recognition element of emixustat.^12,13^ In their *Z*-configurations, these emixustat analogs exhibit weak RPE65 inhibitory activity, but upon photoisomerization to the *E*-configuration they acquire more potent RPE65 inhibitory activity. We show that an *ortho*-fluorinated azobenzene VCM can be photoactivated within the mouse eye to suppress the visual cycle, conferring protection against photic retinopathy. Importantly, the *E*-isomer of this derivative was active after oral administration and displayed a shorter pharmacodynamic duration than emixustat in an electroretinography (ERG) response assay.

## Results

### Installation of the RPE65 Recognition Element on Stilbene and Azobenzene Isosteres Affords a New Class of RPE65 Inhibitors with Isomer-Specific Potency

Emixustat is comprised of two key domains: the γ-amino-α-aryl alcohol we previously described as the RPE65 recognition element^14^ and a methyl cyclohexyl moiety connected through an ether linkage. Our first challenge in developing a photoactivatable molecule was to determine whether the aryl methyl cyclohexyl ether could be substituted for a photoswitchable domain. We chose to use first stilbene and then azobenzene as isosteres to answer this question as their molecular dimensions were similar to those of emixustat and these molecules allowed us to understand the effect of an extended π-system on RPE65 inhibition. As such, we first synthesized two stilbene-containing derivatives **(** ***E*** **)-3** and **(** ***Z*** **)-3** (Figure 1B) using the procedure illustrated in Scheme S1. Briefly, A Suzuki coupling between (*Z*)-(2-bromovinyl)­benzene and (3-formylphenyl)­boronic acid yielded (*Z*)-3-styrylbenzaldehyde (**1a**). A Heck coupling between styrene and 3-bromobenzaldehyde afforded (*E*)-3-styrylbenzaldehyde (**1b**). Subsequent Knoevenagel condensation of **1a** and **1b** with acetonitrile yielded the aldehydes **2a** and **2b** that were reduced with lithium aluminum hydride to afford **(** ***E*** **)-3** and **(** ***Z*** **)-3**. While stilbene moieties can photoisomerize when illuminated with UV light, but when operating under standard laboratory lighting conditions we did not observe this process. Therefore, we evaluated purified **(** ***E*** **)-3** and purified **(** ***Z*** **)-3** as stable, noninterconverting geometric isomers under our assay conditions, allowing us to define the relative RPE65 inhibitory potencies of the two geometries.

We tested **(** ***E*** **)-3** and **(** ***Z*** **)-3** in vitro utilizing bovine RPE microsomes as we previously described,^14^ (Figure 2C) and found that **(** ***E*** **)-3** displayed a half-maximal inhibition concentration (IC_50_) of 494 nM, which was ∼2.5 times higher than that of emixustat (∼200 nM). **(** ***Z*** **)-3** was less potent than **(** ***E*** **)-3**, with an observed IC_50_ of 1.31 μM. These results provided proof of concept that installing an extended π-system in the scaffold of emixustat would retain isomer-specific potency for RPE65 inhibition, providing a rationale for developing a photoactivatable azobenzene-containing RPE65 inhibitor (Figure 1D) that exists in two geometric conformations, **(** ***E*** **)-7** and **(** ***Z*** **)-7**.

**Figure 2:**
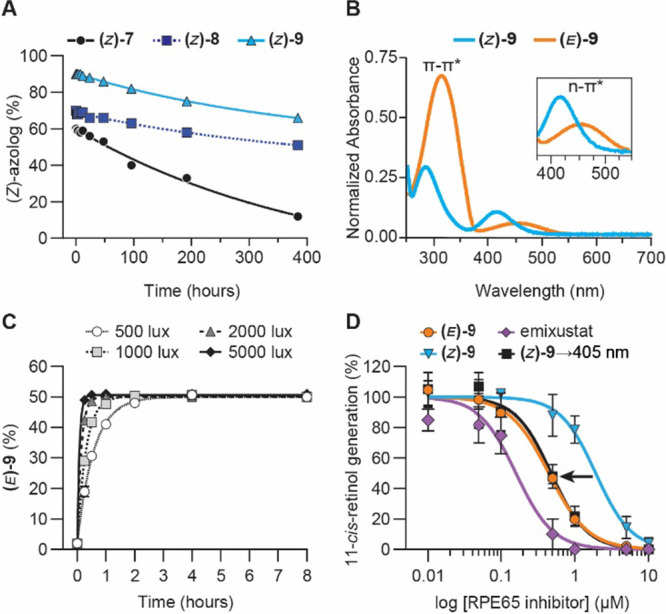
*Ortho*-fluorination yields a stable and photoswitchable azo-emixustat *Z*-isomer that affords potent RPE65 inhibition. (A) Thermal *Z*→*E* isomerization of **(** ***Z*** **)-7**, **(** ***Z*** **)-8**, and **(** ***Z*** **)-9** in PBS supplemented with 10% BSA, at 37 °C, and in the dark. Illustrative thermal isomerization from one of *n* = 3 independent replicates. Curve fitting was performed as described in methods, yielding average half-lives ± SD of 184 ± 96, 914 ± 157, and 926 ± 157 h for **(** ***Z*** **)-7**, **(** ***Z*** **)-8**, and **(** ***Z*** **)-9**, respectively. (B) UV–vis spectra of pure **(** ***E*** **)-9** and **(** ***Z*** **)-9**. The π→π* and n→π* absorbance bands are indicated in the panel, and the n→π* absorption bands are magnified in the inset. (C) Photostationary state (PSS) obtained from a solution of **(** ***Z*** **)-9** (1 mg/mL) under constant illumination with increasing intensities (500–5000 lx) of a xenon light equipped with a filter transparent to visible light. The PSS characterized by an equimolar mixture **(** ***Z*** **)-9** and **(** ***E*** **)-9** isomers, was reached with the following time constants: 34.50 ± 2.10 min (500 lx), 18.00 ± 0.84 min (1000 lx), 8.22 ± 0.24 min (2000 lx), and 4.44 ± 0.78 min (5000 lx). (D) Dose–response curves obtained from RPE65 inhibition in vitro providing the IC_50_ of emixustat (0.160 ± 0.054 μM), **(** ***E*** **)-9** (0.444 ± 0.073 μM), **(** ***Z*** **)-9** (1.921 ± 0.522 μM), and **(** ***Z*** **)-9** together with 405 nm LED light illumination (**(** ***Z*** **)-9**→405 nm, 0.479 ± 0.083 μM). The shift in potency (black arrow) being attributable to photoisomerization of **(** ***Z*** **)-9** to generate the more active **(** ***E*** **)-9** isomer. Data are shown as mean ± SD, *n* = 3. Curve fitting was performed as described in methods.

Azobenzenes undergo light-driven *E*↔*Z* isomerization and the wavelength of light strongly influences the equilibrium of this reaction. UV light excites the π→π* absorption band of both *E* and *Z* isomers while visible light excites n→π* absorption band. Therefore it is possible, in principle, to obtain pure *E* or *Z* by selecting an appropriate wavelength of light.^15^ Because the n→π* bands of the azobenzene VCM isomers overlap (Figure 1E, inset), this photoswitch is nonideal for wavelength-specific photoisomerization with visible wavelengths of light. Thus, we investigated *ortho-*fluorination of the azobenzene moiety as this separates the n→π* bands of the *Z-* and *E-*isomers, allowing wavelength-specific photoswitching in the visible range.^16^ In addition, *ortho*-fluorination markedly prolongs *Z*-isomer thermal lifetimes, enabling selective visible-light isomerization.^16,17^ Based on these considerations and results from **(** ***E*** **)-3** and **(** ***Z*** **)-3**, we generated a working model for a light-activated RPE65 inhibitor VCM wherein a less potent and stable *Z*-isomer could be administered systemically; would remain kinetically stable in vivo; and then be converted to the more potent *E*-isomer within the eye when sufficient photons of light impinge the retina. This model is significantly more compelling when considering that the cornea and lens block UV wavelengths.^18^ As such, only the n→π* band would impact photoconversion.

To implement this design, we installed an *ortho*-fluorinated azobenzene on the RPE65 recognition element of emixustat, yielding the series shown in Figure 1F. Based on our prior work,^19^ *ortho*-fluorination in the 4-position of the emixustat derivatives was expected to be compatible with RPE65 inhibition, but the effect of additional 2-substitution (which would be predicted to enhance *Z*-isomer stability) proved an intractable synthetic hurdle. Broadly speaking, the synthesis of azobenzene-containing emixustat derivatives proved challenging. Scheme S2 summarizes synthetic routes that did not afford the desired azobenzene product. We initially approached this problem utilizing a Knoevenagel-type route analogous to the syntheses of **(** ***E*** **)-3** and **(** ***Z*** **)-3** without success. Next, we attempted a synthetic route similar to our previous publication utilizing a cyclic carbamate to protect the γ-amino alcohol functionality, again without success.^19^ We then tried an enamine-based Heck approach as we published previously, but the azobenzene precursors were not reactive.^20^ Successful synthesis required the construction of the azobenzene functional group as the final step of a sequence due to its incompatibility with the aforementioned approaches. As such, we developed a protecting group-free strategy for functionalized azobenzene synthesis that would chemoselectively tolerate competing amine functional groups and construct the photoswitch as the final step of a pathway.^21^ Based on a detailed account of the conditions required to form azobenzenes in the presence of competing functional groups, we designed a synthetic route to construct an aniline (**6a**) or 1F-*ortho*-aniline (**6b**) derivative of the γ-amino-α-aryl alcohol through a modified Knoevenagel route as shown in Scheme 1.^22^ The nonfluorinated aniline reacted chemoselectively with either nitrosobenzene or 2,6-difluoronitrosobenzene, yielding the pure *E*-isomer of nonfluorinated, **(** ***E*** **)-7**, and difluorinated, **(** ***E*** **)-8**, azo-emixustat derivatives, respectively. The 1F-*ortho*-aniline reacted chemoselectively with 2,6-difluoronitrosobenzene, yielding a trifluorinated azobenzene emixustat analog, **(** ***E*** **)-9**. In all cases the final products were isolated as pure *E-*isomers that did not spontaneously interconvert to the *Z-*isomers in powder form.

**Scheme 1:**
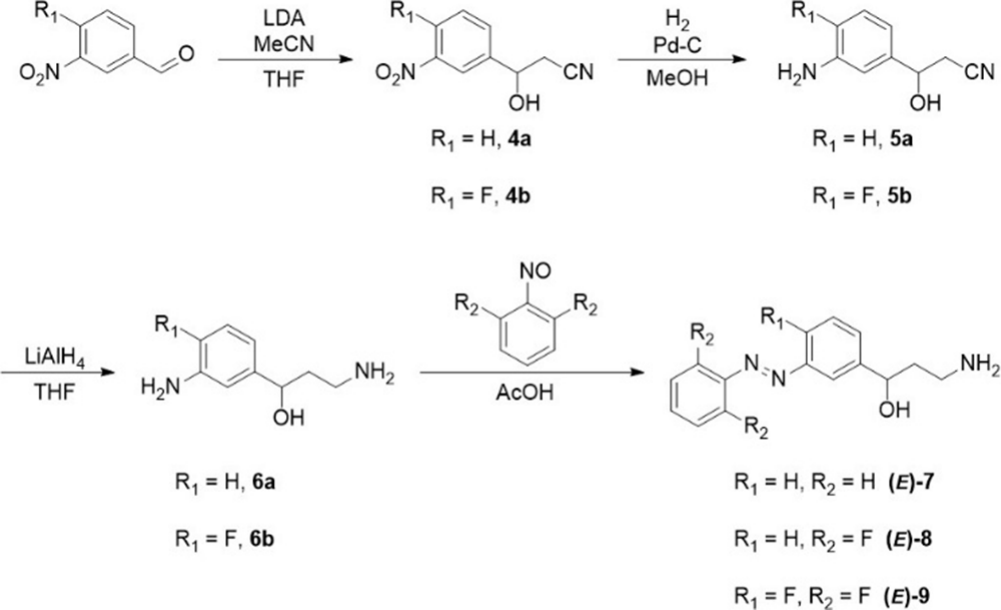
Synthesis of Azo-Emixustat Derivatives

Next, we measured the IC_50_ of **(** ***E*** **)-7** (755 nM) and its *Z*-isomer **(** ***Z*** **)-7** (1.56 μM), respectively, using our in vitro RPE65 assay (Figure 1G). These IC_50_ values were consistent with those observed for the stilbene emixustat derivatives **(** ***E*** **)-3** and **(** ***Z*** **)-3**. Importantly, this result provided the key proof-of-concept data to pursue a model of therapeutic RPE65 inhibition whereby the light illuminating the retina photoconverts a less active azo-emixustat *Z-*isomer into a more active *E*-isomer. At this point we proceeded with a thorough photophysical characterization to determine if **(** ***E*** **)-7**, **(** ***E*** **)-8**, or **(** ***E*** **)-9** would possess characteristics that enable us to test our model.

### Photophysical Characterization of Azobenzene Analogs

Our first task was to determine the optimal photoisomerization conditions to obtain either the *E-* or *Z-*isomer at given wavelengths beginning with the pure *E* synthetic product. Most azobenzenes with and without *ortho*-fluorination photoisomerize in the UV–vis range between 250 and 460 nm.^16,17^ Thus, starting from freshly HPLC-purified *E*-isomers, we measured the photostationary state (PSS) obtained with five different wavelengths of light, ranging from 254 to 460 nm, and expressed it as a % of *Z* (Table 1). In this experiment, the azobenzenes were solubilized in methanol at a concentration of 1 mg/mL. For all test compounds [**(** ***E*** **)-7**, **(** ***E*** **)-8**, and **(** ***E*** **)-9**], 300 nm yielded the PSS most enriched in *Z-*isomer (79.0 < PSS < 83.0%), and 395/419 nm yielded the least enriched PSS in *Z-*isomer (29.5 < PSS < 39.5%). Irradiation at 460 nm also led to PSS enriched in *Z*, attaining 49.5 < PSS < 60.0% while illumination with 350 nm light led to variable PSS among the test compounds (36.5 < PSS < 83.0%). Instead, 254 nm light led to PSS being poor in *Z* for all test compounds (35.5 < PSS < 36.5%). Thus, the illumination with 300 nm light was used to prepare PSS enriched in *Z* isomers for purification by preparative HPLC. Also, this experiment indicated that violet light (395/419 nm) would favor the *Z*→*E* photoisomerization of our azo-emixustat derivatives for our future experiments.

**Table 1:** Photostationary States of Emixustat Azologs (1 mg/mL in Methanol) Obtained by Illuminating Pure *E*-Isomers in Different Wavelengths of Light for 1 h

wavelength (nm)	(*Z*)-7%	(*Z*)-8%	(*Z*)-9%
254	35.5 ± 1.5	36.0 ± 2.0	36.5 ± 2.0
300	83.0 ± 3.0	79.5 ± 0.5	79.0 ± 1.0
350	83.0 ± 1.0	36.5 ± 2.5	63.0 ± 1.0
395/419	39.5 ± 1.5	35.0 ± 1.0	29.5 ± 0.5
460	49.5 ± 2.5	56.6 ± 0.5	60.0 ± 0.1

We then measured the kinetic stability of these HPLC purified *Z-*isomers (Figure 2A). Based on our model, we sought to deliver a kinetically stable *Z*-isomer to the retina in vivo where it would be photoactivated. After preparative HPLC, the sample was immediately assessed by analytical HPLC, providing baseline purity near 100%. Next, the *Z-*isomers were concentrated in vacuo and 1 mg/mL solutions in 10 mM PBS pH 7.4 were prepared. Although performed under dim red light, together these operations led to a decrease in *Z*-isomer composition. Immediately after resolubilization in PBS, analytical HPLC showed *Z* abundances of 56, 66, and 89% for **(** ***Z*** **)-7**, **(** ***Z*** **)-8**, and **(** ***Z*** **)-9**, respectively. Kinetically, the *Z*-isomer composition of nonfluorinated **(** ***Z*** **)-7** decreased to 18% after 16 days, while that of the difluorinated **(** ***Z*** **)-8** and trifluorinated **(** ***Z*** **)-9** decreased to 49 and 66%, respectively. The fitting of these data to the exponential decay equation produced the following half-lives: 184 ± 96, 914 ± 157, and 926 ± 157 h for **(** ***Z*** **)-7**, **(** ***Z*** **)-8**, and **(** ***Z*** **)-9**, respectively (Figure S1). The maximal to minimal *Z*-isomer kinetic stability of **(** ***Z*** **)-9** > **(** ***Z*** **)-8** > **(** ***Z*** **)-7**, although the difference between the half-lives of **(** ***Z*** **)-8** and **(** ***Z*** **)-9** was within the experimental error. Importantly, these data were consistent with the previously noted observations that *ortho*-fluorination of azobenzenes improves the kinetic stability of the *Z-*isomers.^16^ Based on our proposed model, we chose to move forward with **(** ***E*** **)-9** and **(** ***Z*** **)-9** because of the more favorable PSS in the 395/419 nm light and observed kinetic stability of **(** ***Z*** **)-9**. It is also worth noting that compound **8** showed apparent chemical instability during purification and analytical handling, distinct from its measured geometric isomer stability, and this limited further exploration. Next, we measured the thermal stability of **(** ***Z*** **)-9** in DMSO and PBS supplemented with 10% fetal bovine serum (FBS) and found that this fluorinated azo-emixustat derivative was stable for up to 24 h at 37 °C and for up to 1 week when frozen in DMSO at −80 °C (Figure S2). This last data gave us the confidence to prepare frozen aliquots of **(** ***Z*** **)-9** in DMSO for storage. Subsequently, we measured the UV–vis absorption spectra of **(** ***E*** **)-9** and **(** ***Z*** **)-9** (Figure 2B). Consistent with prior literature,^16^ *ortho*-fluorination separated the n−π* bands of the fluorinated azo-emixustat geometric isomers (Figure 2B, inset). Importantly, this result explained the higher enrichment of **(** ***Z*** **)-9** in the PSS of blue light (460 nm, 60% **(** ***Z*** **)-9**) compared to violet light (395/419 nm, 30%) (Table 1) as due to differences of the extinction coefficient between **(** ***E*** **)-9** and **(** ***Z*** **)-9** in this region of the visible spectrum. In blue light, **(** ***E*** **)-9** possesses a higher absorptivity than **(** ***Z*** **)-9**, slightly favoring the *E*→*Z* photoconversion. The opposite happens in violet light where the extinction coefficient of **(** ***Z*** **)-9** is higher than **(** ***E*** **)-9**, favoring *Z*→*E* photoconversion. We then determined the PSS of **(** ***Z*** **)-9** (1 mg/mL) in broad-spectrum white light emitted from a xenon lamp equipped with a filter transparent only to visible wavelengths of light (400–700 nm). This setup mimicked the lighting conditions at the level of the retina because the structures of the anterior segment of the eye (cornea and lens) do not transmit UV light.^18^ Thus, we measured the kinetics by which **(** ***Z*** **)-9** reaches the PSS when illuminated with our white light setup (Figure 2C). In this experiment, we tested four intensities of white light (500, 1000, 2000, and 5000 lx) corresponding to the lighting conditions varying from bright sunlight to a typical overcast day and found that the maximal attainable enrichment of **(** ***E*** **)-9** was 50% for all intensities. However, the time constants to reach this PSS were 4.44, 8.22, 18.00, and 34.50 min for 5000, 2000, 1000, and 500 lx, respectively, demonstrating tunable **(** ***Z*** **)-9**→**(** ***E*** **)-9** photoisomerization as a function of light intensity. Similar observations were published by others where aqueous solutions of *ortho*-fluorinated azobenzenes were exposed to ambient light.^23^ Thus, the PSS of **(** ***Z*** **)-9** in UV-deprived white light can be expected to be reached faster on a sunny day as compared to dim light conditions, highlighting on-demand **(** ***Z*** **)-9**→**(** ***E*** **)-9** photoconversion.

We then designed an in vitro experiment to test our model of delivering a less active *Z*-isomer and photoactivating in situ*.* First, we measured the impact of 10 min of illumination with 385–400 nm light on our RPE65 in vitro assay and found that the RPE65 activity was comparable to the same reaction in the dark (Figure S3). By contrast, emixustat treatment abolished RPE65 activity, validating the robustness of our in vitro system as a tool to study the photoconversion of *Z*-isomer in situ. Thus, we measured the potency of pure **(** ***Z*** **)-9** and **(** ***E*** **)-9** in the dark using our standard RPE65 in vitro assay (Figure 2D). **(** ***E*** **)-9** displayed an IC_50_ of 444 nM (orange curve), which was ∼4.5 times less potent than that of **(** ***Z*** **)-9** (1.921 μM, blue curve). These results showed a larger, net ∼5-fold separation between the potencies of the two geometric azobenzene isomers, which was superior to the net ∼2-fold separation observed for the nonfluorinated **(** ***Z*** **)-7** and **(** ***E*** **)-7**. These measurements define the limiting activities of the two purified geometric isomers. Next, to obtain a functionally relevant photoactivated readout for the azobenzene scaffold, we incubated **(** ***Z*** **)-9** with RPE microsomes concurrent with 395/419 nm illumination. Thus, the illuminated **(** ***Z*** **)-9** curve in Figure 2D represents the PSS-relevant activity experiment under the actual microsomal assay conditions, rather than the activity of a separately prepared bulk PSS mixture. We predicted that in situ photoisomerization would occur and the system would display an IC_50_ reflective of the observed PSS (∼70% **(** ***E*** **)-9**, see Table 1). Instead, this experiment yielded an IC_50_ of 479 nM (black curve), which effectively replicated that of pure **(** ***E*** **)-9** (444 nM) and based on this, our initial interpretation was that the yield of photoisomerization was higher in this reaction system compared to that observed with PBS alone. Collectively, these results demonstrate that **(** ***Z*** **)-9** met the design criteria for an ocular photopharmacologic agent: (i) high *Z*→*E* photoisomerization yield in the visible spectrum, (ii) robust kinetic stability in its *Z*-isomer, and (iii) significantly enhanced RPE65 inhibition upon photoactivation. Based on these data we determined these properties warranted further in vitro and in vivo evaluation.

### Binding to RPE65 Distorts the Planarity of the **(*E*)-9** π System

To gain an understanding of how **(** ***E*** **)-9** is accommodated in the RPE65 active site, we determined the crystal structure of RPE65 in a complex with **(** ***E*** **)-9** (Table S1). **(** ***E*** **)-9** binds to RPE65 in a 1:1 stoichiometry (Figure 3A) and occupies the entrance of the RPE65 active site in the presence of a palmitate molecule that occupies the deeper part of the catalytic site, like in other structures of RPE65 complexed with emixustat-like derivatives.^5,12,13,19^ Notably, the *ortho*-fluorinated azobenzene π system did not disrupt the key polar interactions of the RPE65 recognition element with Thr147, Glu148, and the carboxylic acid of the palmitate. Instead, it provided an additional polar interaction between the fluorine atom of the 1F-*ortho*-aniline derivative of the γ-amino-α-aryl alcohol and Tyr275, as was observed in one of our prior publications.^19^

**Figure 3:**
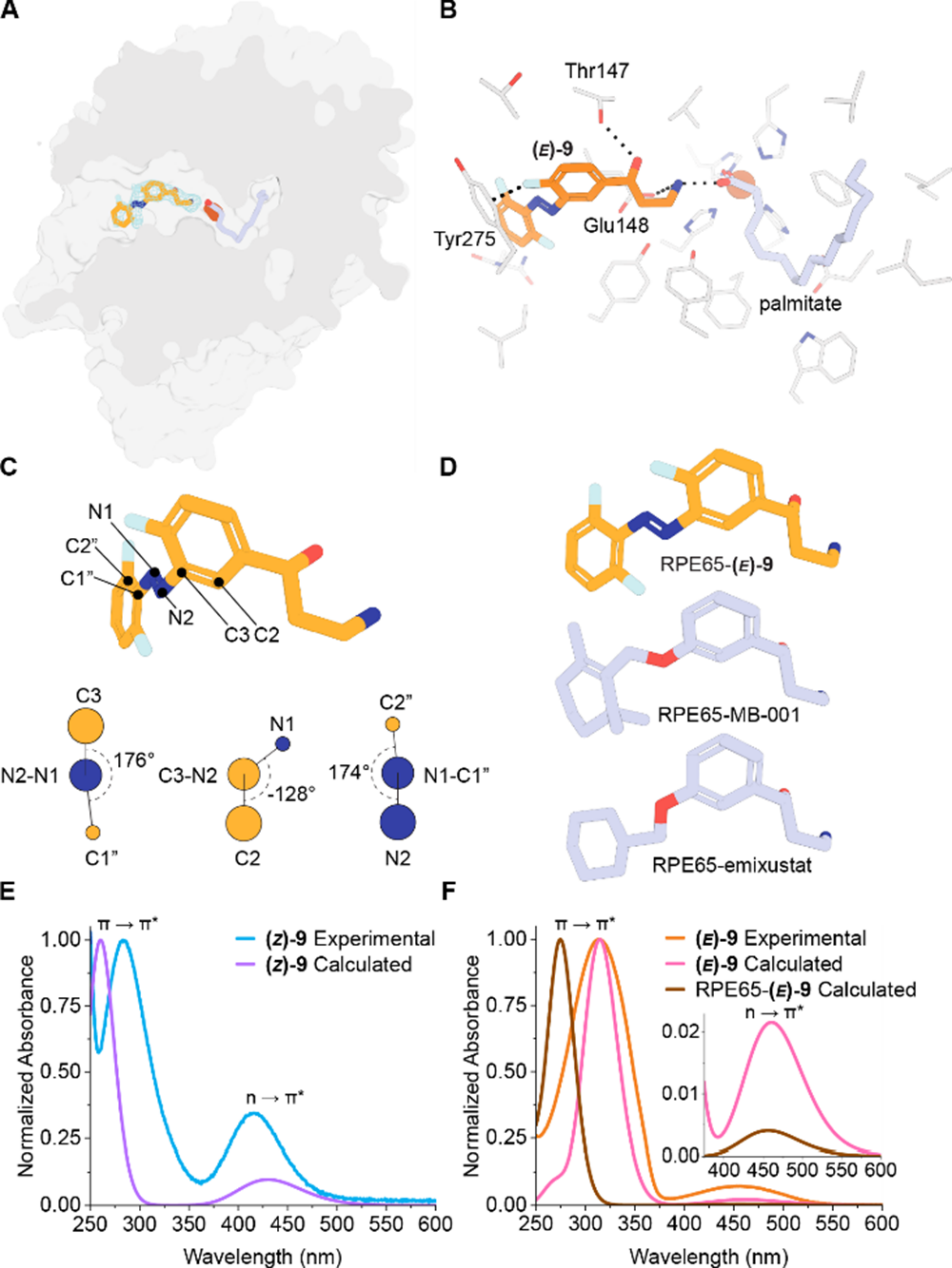
Crystal structure of bovine RPE65 in a complex with **(** ***E*** **)-9**. (A) Cut-through view of the RPE65 active site showing the binding location of **(** ***E*** **)-9** as well as a coordinated palmitate ligand. The corresponding *F*_o_–*F*_c_ electron density map, contoured at 3 RMSD, is shown as green mesh within 3 Å of the bound **(** ***E*** **)-9**. PDB accession code 9OA5. (B) Illustration of the RPE65 residues within 4.5 Å from the bound ligands. The hydrogen bonds with Thr147, Glu148, Tyr275, and the palmitate are indicated by dashed lines. (C) Chemical structure of **(** ***E*** **)-9** as from the crystal structure with the nitrogen, oxygen, and carbon atoms highlighted in blue, red, and gray, respectively. Below, the torsion angles along the N2–N1, C3–N2, and N1–C1” bonds are shown. (D) Comparison of the binding orientations between **(** ***E*** **)-9**, MB-001 (PDB accession code 4RSE), and emixustat (PDB accession code 4RSC) in the RPE65 active site. (E) Calculated time-dependent density functional theory (TDDFT) UV–Vis absorption spectrum of **(** *Z* **)-9** in solution (see Methods, wB97x model shown), showing a distinct n→π* feature in the visible region in addition to the near-UV π→π* transition. (F) TDDFT spectrum computed for the RPE65-bound conformation of RPE65-**(** ***E*** **)-9**, in which the n→π* transition is markedly attenuated, consistent with binding-induced twisting of the azobenzene core that is expected to reduce the propensity for visible-light isomerization.

Interestingly, the most striking observation we made when investigating the binding of our *ortho*-fluorinated **(** ***E*** **)-9** azobenzene to RPE65 was a deviation from the planarity of the azobenzene π system as illustrated in the top panel of Figure 3C. These deviations from planarity, shown in the bottom panels of Figure 3C, is attributable to the binding constraints characteristics of the RPE65 catalytic pocket as illustrated by the comparison between the bound conformation of **(** ***E*** **)-9** with that of other potent VCMs, emixustat and MB-001 (Figure 3D). We then studied whether this deviation from planarity would have a substantial impact on the photophysical properties of **9** bound to RPE65 (RPE65-**(** ***E*** **)-9**). To test this, we first computed time-dependent density functional theory (TDDFT) UV–Vis spectra of optimized structures of **(** ***Z*** **)-9** and **(** ***E*** **)-9** (Figures 3E,F and S4) to determine if the n→π* and π→π* bands of the *Z-* and *E-*isomers could be accurately predicted by theory. What we observed was that while the absolute position was variable, the relative intensities of the n→π* and π→π* absorption bands were well predicted. We then used the same approach to predict the relative intensities of the n→π* and π→π* bands of RPE65-**(** ***E*** **)-9**. Interestingly, when using the model (wB97x) that most accurately predicted the experimental spectra (see Table S2 for all calculated parameters), the calculations showed a near complete suppression of the n→π* absorption band while retaining a strong π→π* absorption band. These calculations suggest one possible explanation for the contrasting results shown in Table 1 and Figure 2D. Specifically, attenuation of the n→π* absorption band in the RPE65-bound conformation of **(** ***E*** **)-9** could reduce **(** ***E*** **)-9→(** ***Z*** **)-9** photoreversal and thereby contribute to the stronger-than-expected activity observed in the illuminated microsomal assay. Specifically, when pure **(** ***E*** **)-9** was irradiated with 395/419 nm light the PSS featured a 70% enrichment of the *E-*isomer while the incubation of **(** ***Z*** **)-9** with RPE microsomes followed by 395/419 nm irradiation replicated the IC_50_ of pure **(** ***E*** **)-9**, indicating that there was a factor(s) in the **(** ***Z*** **)-9**-microsome system boosting the photoequilibrium toward pure **(** ***E*** **)-9**. However, these calculations do not establish that compound **9** photoisomerizes while fully bound to RPE65, nor do they exclude contributions from other factors, including the photoequilibrium achieved in the assay medium or light-independent/thermal isomerization. We therefore interpret the bound-state TDDFT analysis as a mechanistic hypothesis rather than a definitive explanation. Based on these compelling in vitro results we proceeded to evaluate **(** ***Z*** **)-9** and **(** ***E*** **)-9** in vivo.

### In Vivo Pharmacodynamic Properties of **(*E*)-9** and **(*Z*)-9**

To investigate the ability of **(** ***E*** **)-9** and **(** ***Z*** **)-9** to modulate the visual cycle (via RPE65 inhibition) in vivo, we examined whether systemic administration would result in differences in recovery of visual chromophore as assessed by scotopic electroretinogram (ERG) signal after exposure to white light, which bleaches >90% of visual pigment. In this experiment, dark-adapted BALB/cJ mice were subjected to a 10 min, 10,000 lx photobleach with white LED light and then received a single intraperitoneal (IP) injection of either vehicle (DMSO) or test compounds (1, 5, or 10 mg/kg) in the dark followed by 2 h of rearing in darkness to allow rod photoreceptors to recover their sensitivity (Figure 4A). The spectral properties of the white LED cluster used for the photobleach are shown in Figure S5. The pattern of scotopic ERG of dark-adapted mice (black trace) closely resembled that of mice treated with vehicle (dark gray trace), featuring pronounced a- and b-waves. In contrast, the mice tested immediately after the photobleach (light gray trace) did not feature the a- and b-waves. As expected, emixustat treatment (purple traces) suppressed the ERG at all doses, leading to patterns like that of the photobleached group. In contrast, the suppression of the scotopic ERG induced by **(** ***E*** **)-9** (orange traces) and **(** ***Z*** **)-9** (blue traces) was dose-dependent, showing that these azo-emixustat derivatives are less potent than emixustat also in vivo. Notably, at the dose of 5 mg/kg, **(** ***E*** **)-9** led to nearly quantitative ERG suppression that was significantly higher than that of **(** ***Z*** **)-9**, which suppressed 80% of the a-wave and 62% of the b-wave, demonstrating the inferior potency of the *Z* isomer in vivo. Figure 4B,C show the quantification of the a- and b-wave amplitudes.

**Figure 4:**
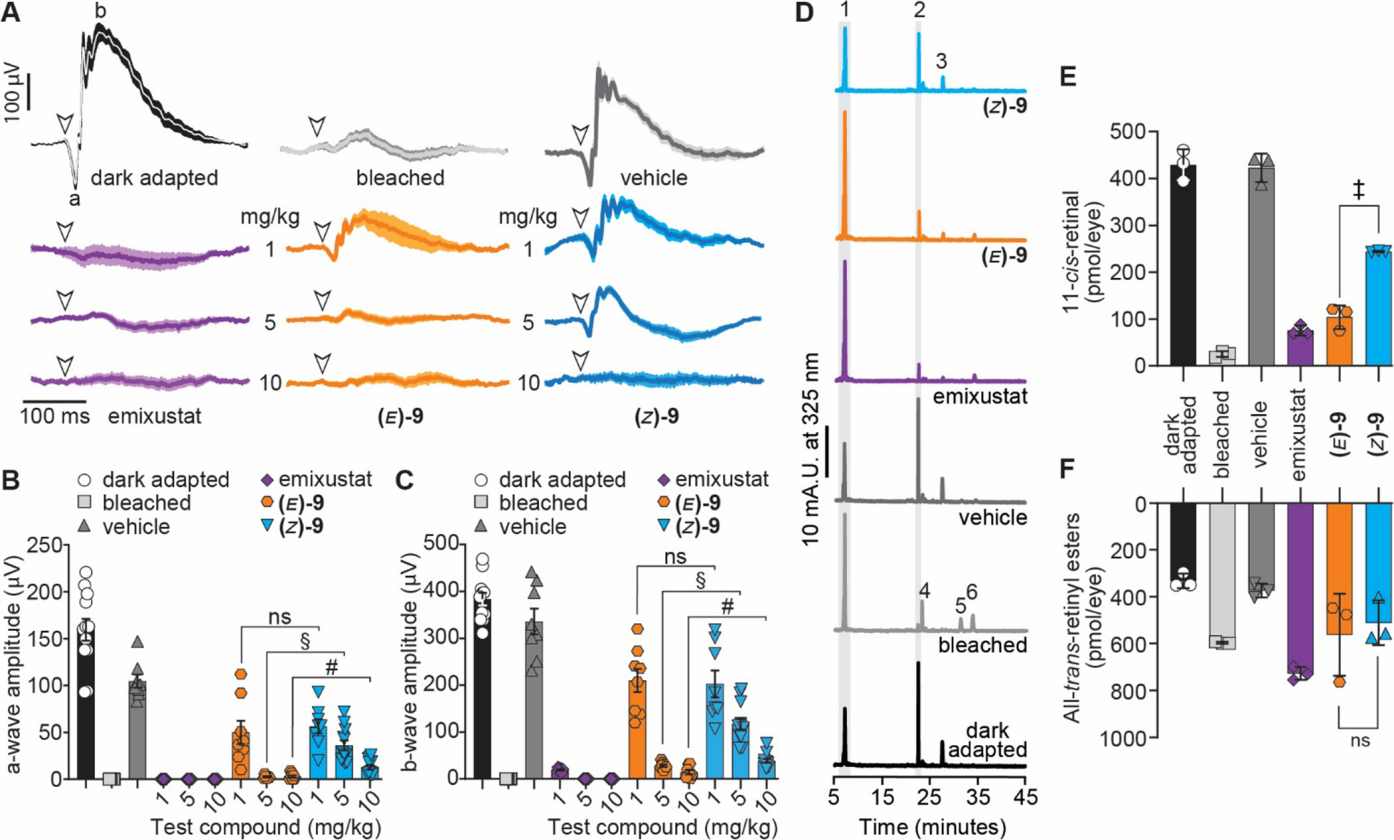
**(*E*)-9** is a more potent RPE65 inhibitor than **(*Z*)-9** in vivo. (A) Scotopic ERG responses from 6 to 8 weeks old BALB/cJ obtained using a flash intensity of 0.1 cd · s/m^2^. The white arrows indicate the time of flash delivery. Dark-adapted mice were administered a single IP injection of vehicle or test compounds (1, 5, or 10 mg/kg) in the dark, photobleached (10 min, 10,000 lx), and then housed in the dark for 2 h before scotopic ERG assessment. The data are shown as means (solid lines) ± SEM (uncertainty band), *n* = 6 or 12 eyes per group. (B, C) Quantification of scotopic a- and b- waves, respectively. Data represent mean ± SEM, each data point corresponds to one eye. The results from the *t* test are reported as ^ns^ *p* > 0.05, *p < 0.05, ^#^ *p* < 0.01, ^‡^ *p* < 0.001, ^§^ *p* < 0.0001. (D) Raw HPLC chromatograms showing the effects of vehicle or test compounds (5 mg/kg) on the retinoid extracted from whole mouse eye homogenates. This experiment was run using the same protocol as in panel A. The peak identity is as follows: 1, all-*trans*-retinyl esters; 2, *syn*-11-*cis*-RAL oxime; 3, *anti*-11-*cis*-RAL oxime; 4, *syn*-all-*trans*-RAL oxime; 5, *anti*-all-*trans*-RAL oxime; 6, all-*trans*-ROL. (E, F) Absolute 11-*cis*-RAL and all-*trans*-retinyl esters quantification from mouse eyes. Data represent mean ± SD, each data point corresponds to one animal. The results from the *t* test are reported as ^ns^ *p* > 0.05,*p < 0.05, ^#^ *p* < 0.01, ^‡^ *p* < 0.001, ^§^ *p* < 0.0001.

To confirm that the observations from scotopic ERG experiments corresponded to a differential inhibition of RPE65 (due to the relative potencies of **(** ***E*** **)-9** and **(** ***Z*** **)-9**), we repeated the same experiment described above, but evaluated the status of visual pigment regeneration in the mouse eyes by analyzing the composition of the retinoid pool, specifically by quantitating retinyl esters and 11-*cis-*RAL via HPLC (Figure 4D). If RPE65 were inhibited we would predict that retinyl esters would accumulate as RPE65’s substrate, and 11-*cis*-RAL levels would diminish. The HPLC chromatograms from dark adapted (black) and vehicle-treated (dark gray) mice were similar, featuring a small peak corresponding to the all-*trans*-retinyl esters (1) and the two large peaks corresponding to the two oximes formed from 11-*cis*-RAL (2 and 3, *syn* and *anti*, respectively) produced from derivatization with hydroxylamine, a necessary step to effectively extract RALs from tissues. The chromatogram obtained from bleached mice (light gray), featured a large all-*trans*-retinyl ester peak together with peaks for *syn- and anti*-all-*trans*-RAL oximes (4 and 5, respectively) and all-*trans*-ROL, while 11-*cis*-RAL oxime peaks were greatly diminished, indicating near a quantitative visual pigment photobleach and partial reduction of the released all-*trans*-RAL. As expected, the chromatogram from emixustat treatment (purple) showed a large all-*trans*-retinyl ester peak and exceptionally small 11-*cis*-RAL peak, signifying strong suppression of RPE65 activity. The chromatogram from **(** ***E*** **)-9** treatment (orange) matched that of emixustat while the chromatogram of **(** ***Z*** **)-9** treatment (blue) featured a smaller all-*trans*-retinyl esters peak and a ∼50% recovery of the 11-*cis*-RAL peak, confirming that the suppression of the scotopic ERG effects we observed could be directly connected to RPE65 inhibition. Additionally, these data demonstrated the lower potency of **(** ***Z*** **)-9** in vivo compared to **(** ***E*** **)-9**. The absolute quantification of 11-*cis*-RAL and all-*trans*-retinyl esters is shown in Figure 4E,F, respectively. Together, these results motivated the pursuit of using **(** ***Z*** **)-9** as a less active VCM photoswitch for light-triggered visual cycle modulation in vivo.

### Violet Light Photoswitchs **(*Z*)-9** in the Living Mouse Eye

To determine whether **(** ***Z*** **)-9→(** ***E*** **)-9** photoisomerization could occur within the matrix of a living mouse eye we developed a multiple reaction monitoring (MRM) triple quadrupole LC-MS (LC-MS/MS) method to quantify the ratio of **(** ***E*** **)-9** over **(** ***Z*** **)-9** utilizing **(** ***E*** **)-3** as an internal standard (Figure S6). In this experiment, 6–8 weeks-old BALB/cJ mice received a single IP injection of pure **(** ***Z*** **)-9** (5 mg/kg) in the dark. Thirty min later, the mice were either exposed to 10 min of 405 or 630 nm (negative control) LED light or kept in the dark for the same amount of time and immediately euthanized, their eyes were collected and homogenized to measure the respective PSS (Figure 5).

**Figure 5:**
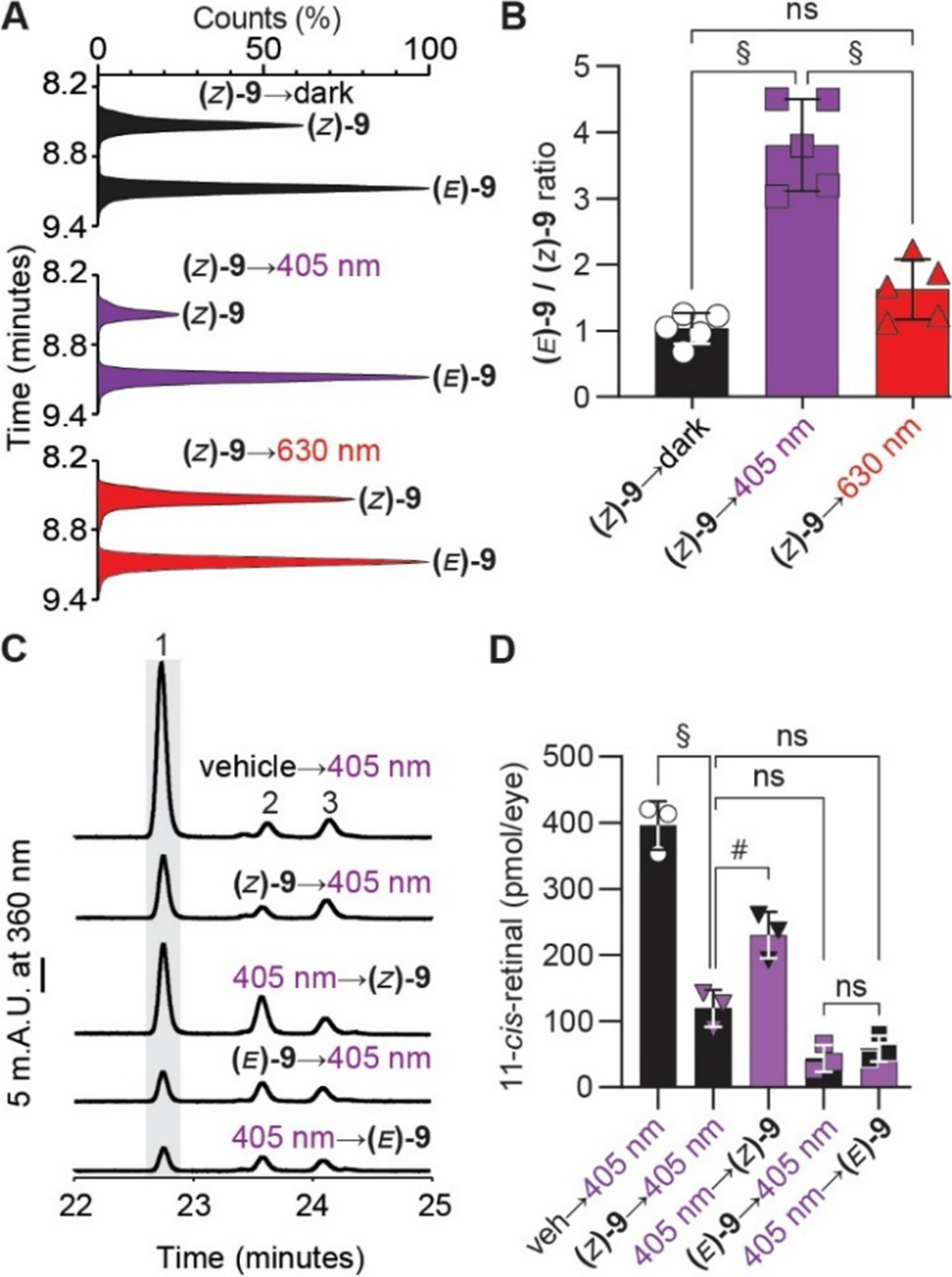
violet light photoisomerizes **(*Z*)-9** in the retina. (A) Raw LC-MS/MS chromatograms showing the isomeric distribution of **9** in whole eye extracts following systemic administration of pure **(** ***Z*** **)-9** in the dark and after exposure to 405 or 630 nm light. In this experiment, 6–8 weeks-old BALB/cJ mice received a single IP injection of **(** ***Z*** **)-9** (5 mg/kg) in the dark, followed by 10 min of dark housing or 10 min illumination with 405 or 630 nm LED light. The black arrow (→) indicated the order of drug administration and illumination. (B) **(** ***E*** **)-9**/**(** ***Z*** **)-9** ratio obtained by quantification of the LC-MS/MS data. Data represent mean ± SEM and each data point corresponds to one mouse, *n* = 5. ANOVA analysis showed a statistically significant difference between the groups (*p* < 0.0001). The results of Dunnett’s multiple comparison test are reported in the panel. (C) Raw HPLC chromatograms showing the effect of a 5 min exposure to 405 nm LED light before and after a single IP injection of test compounds (**(** ***Z*** **)-9** and **(** ***E*** **)-9**) on visual cycle activity. Mice were dark-adapted for 8 h following the IP injection and light exposure, then euthanized and their eyes collected for retinoid analysis by HPLC. Peak identities are as follows: 1, *syn*-11-*cis*-RAL oxime; 2-, *syn*-all-*trans*-RAL oxime; 3, *syn*-13-*cis*-RAL oxime. (D) Absolute 11-*cis*-RAL quantification from the eyes of mice treated with vehicle or test compounds (5 mg/kg) and 405 nm LED lights. ANOVA showed a significant effect of the test compounds (*p* < 0.0001). The results of Dunnett’s multiple comparison test are reported in the figure. Data represent mean ± SD, each data point corresponds to one mouse. Abbreviations: ^ns^ *p* > 0.05, *p < 0.05, ^#^ *p* < 0.01, ^‡^ *p* < 0.001, ^§^ *p* < 0.0001.

The spectral properties of the LED lights used in this experiment are shown in Figure S5. The raw LC-MS/MS chromatograms obtained from this experiment are shown in Figure 5A, and the ratio of **(** ***E*** **)-9** over **(** ***Z*** **)-9** is shown in Figure 5B. Interestingly, in the absence of light we found roughly equal amounts of **(** ***Z*** **)-9** and **(** ***E*** **)-9** (black peaks and bar), indicating the presence of a light-independent **(** ***Z*** **)-9→(** ***E*** **)-9** isomerization mechanism(s) in vivo that could explain the relatively small (∼5-fold) separation of **(** ***E*** **)-9** than **(** ***Z*** **)-9** IC_50_ values in vitro. In contrast, a 10 min exposure to 405 nm LED light yielded net 4-fold more **(** ***E*** **)-9** than **(** ***Z*** **)-9** (purple peaks and bar), demonstrating the significant effect of violet light on the **(** ***Z*** **)-9→(** ***E*** **)-9** isomerization in the living mouse eye. Instead, illumination with 630 nm red LED light led to a net 1.5-fold increment of **(** ***E*** **)-9** over **(** ***Z*** **)-9** (red peaks and bar), which is not significant compared to the group left in the dark. Importantly, these results were consistent with the data obtained from studying the optimal wavelength for **(** ***Z*** **)-9→(** ***E*** **)-9** photoisomerization (Table 1) and explained the observed RPE65 inhibition of the **(** ***Z*** **)-9** isomer in vivo as due to a partial, light-independent isomerization into the more potent **(** ***E*** **)-9** isomer.

Having demonstrated the capacity of **(** ***Z*** **)-9** to photoisomerize due to light penetration into the outer retina, we next asked whether such conversion can be exploited for on-demand visual cycle inhibition in a living mouse. The 405 nm (violet) light that optimally photoconverts **(** ***Z*** **)-9** can also photobleach visual pigments (Figure S7), albeit less efficiently compared to green light. Thus, for experimental simplicity, we used 405 nm light for visual pigment photobleaching and to effect **(** ***Z*** **)-9→(** ***E*** **)-9** photoisomerization. We studied the effect of administering **(** ***Z*** **)-9** or **(** ***E*** **)-9** to mice either 5 min before or immediately after 5 min of violet light illumination. Whereas visual pigment bleaching is equivalent in the two dosing schedules, only in the former schedule does **(** ***Z*** **)-9** have the opportunity to be photoactivated during the bleaching light exposure. Afterward, the mice were housed in the dark for 8 h, euthanized, and their eyes collected for quantification of 11*-cis*-RAL by HPLC. To address the concern that prebleach compound administration could be intrinsically more visual cycle suppressive due to the extra time available for ocular distribution, we first tested the efficacy of visual cycle suppression exerted by **(** ***E*** **)-9** dosed pre- and postillumination. Importantly, **(** ***E*** **)-9→(** ***Z*** **)-9** photoisomerization is expected to be negligible at this wavelength (Table 1 and Figure 2D). As compared to mice treated with vehicle control, which completely recovered their 11*-cis*-RAL levels, mice treated with either dosing regimen of **(** ***E*** **)-9** had strong and equivalently suppressed 11*-cis*-RAL recovery (Figure 5C,D). Notably, this result demonstrated that **(** ***E*** **)-9** rapidly distributes to its target site in the RPE and that negligible 11*-cis*-RAL recovery occurs during the time lag separating the pre- and postillumination regimens. With this important control established, we tested **(** ***Z*** **)-9** in the same experimental paradigm. As shown in Figure 5C,D, prebleach administration of **(** ***Z*** **)-9** resulted in ∼2-fold less regeneration of 11*-cis*-RAL as compared to postbleach administration, a difference we ascribe to **(** ***Z*** **)-9→(** ***E*** **)-9** photoactivation occurring in the former treatment schedule. These data thus support the ability of **(** ***Z*** **)-9** to be photoactivated in the living eye upon exposure to light of appropriate energy and intensity to exert a pharmacological effect on the visual cycle.

### Administration of **(*E*)-9** Prevents Retinal Damage Induced by Aberrant Visual Cycle Activity

Having established that systemically administered **(** ***Z*** **)-9** can photoisomerize within the living eye and acutely modulate visual-cycle activity, we next asked whether the active isomer of this azobenzene scaffold retains protective efficacy in a model of light-induced retinal injury. Because compound **9** still undergoes appreciable light-independent/thermal **(** ***Z*** **)**→**(** ***E*** **)** conversion in vivo, a photoprotection experiment initiated from systemically administered **(** ***Z*** **)-9** would be difficult to interpret unequivocally at this stage. We therefore utilized **(** ***E*** **)-9** in this initial study to determine whether the active isomer of the scaffold is itself capable of reproducing the protective effect of emixustat^24^ and analogs.^12,13^ For this experiment, we administered a single IP injection of either vehicle or test compounds (emixustat or **(** ***E*** **)-9**, 10 mg/kg) to 6–8 weeks-old BABL/cJ mice in the dark. Thirty min later, the mice were exposed to 8 h of white 15,000 lx white LED light, followed by rearing in a regular light/dark (12*h*/12h) cycle for 1 week. Next, the structure of the retina was examined by scanning laser ophthalmoscopy (SLO) and optical coherence tomography (OCT) while retinal function was examined by ERG of dark-adapted mice. Eventually, the mice were euthanized, and their eyes were processed for histological examination. These data are illustrated in Figure 6.

**Figure 6:**
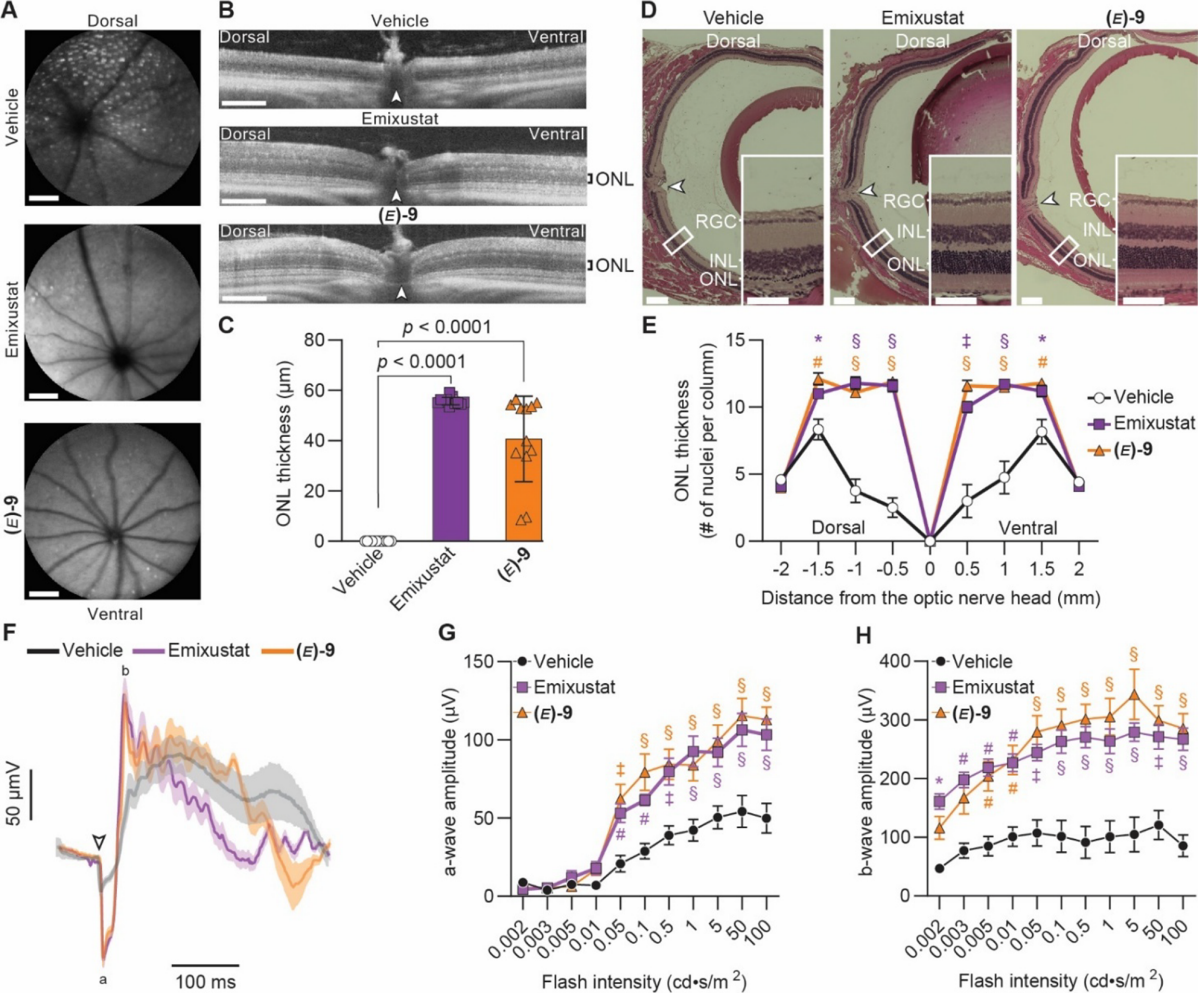
**(*E*)-9** prevents photic retinal damage in BALB/cJ mice. (A) Scanning laser ophthalmoscopy images showing the effect of a single IP injection of vehicle or test compounds (emixustat and (***E*)-9**, 10 mg/kg) on retinal autofluorescence 1 week after inducing retinal damage with an 8 h long exposure to a 15,000 lx white LED light. Scale bar 500 μm. (B) Corresponding retinal optical coherence tomography (OCT) images. Test compounds maintain an intact outer nuclear layer (ONL) (black brackets). The white arrows indicate the optic nerve head. Scale bar 200 μm. (C) ONL thickness as measured in OCT images at 500 μm from the optic nerve head. Data represent means ± SEM, and circles individual eyes, *n* = 12. ANOVA analysis showed a significant effect of test compounds (*p* < 0.0001). The results of multiple comparison test are reported in the figure. (D) Images of retinal cross sections stained with H&E; scale bars 100 μm. The white arrows indicate the optic nerve head. The white rectangles correspond to the area magnified in the insets; scale bar 50 μm. Abbreviations: retinal ganglion cell layer (RGC); inner nuclear layer (INL). (E) Spider plot showing the thickness of ONL. Data represent mean ± SEM, *n* = 10. ANOVA analysis showed a significant effect of test compounds (*p* < 0.0001). The results of multiple comparisons are shown in the figure. (F) Scotopic ERG swipes measured using a flash intensity of 0.1 cd · s/m^2^. The black arrowheads indicate the timing of test flash delivery. The data are shown as means (solid traces) ± SEM (contour), *n* = 10 or 12 eyes per group. (G, H) Quantification of scotopic a-wave and b-wave amplitudes, respectively. ANOVA analysis showed a significant effect of the test compounds on the amplitude of a- (*p* = 0.0005) and b- (*p* = 0.0016) waves. The results of the multiple comparisons test are reported in the figure (*p < 0.05, ^#^ *p* < 0.01, ^‡^ *p* < 0.001, ^§^ *p* < 0.0001).

SLO showed the appearance of autofluorescent puncta in the retina of mice treated with vehicle (Figure 6A). These autofluorescent puncta were less present in the groups treated with emixustat and **(** ***E*** **)-9**. OCT retinal images highlighted the disappearance of the outer nuclear layer (ONL), the retinal layer where the photoreceptor soma localizes, within ± 750 μm from the optic nerve in the group treated with vehicle (Figure 6B). In contrast, emixustat and **(** ***E*** **)-9** afforded a significant preservation of the ONL within ± 750 μm from the optic nerve (black brackets). Specifically, **(** ***E*** **)-9** afforded ∼70% of the protective effect provided by emixustat, as shown in Figure 6C. Histological analysis of these animals (Figure 6D) showed that the vehicle treatment did not afford the ONL protection along the ventral-dorsal axis within ± 2 mm from the optic nerve, while the ONL of the emixustat and **(** ***E*** **)-9** groups was preserved throughout the retinal cross sections (Figure 6E). The pattern of ONL degeneration in the vehicle group was consistent with prior literature describing light damage in mice.^25^ Figure 6F shows the results obtained from ERG measurements of dark-adapted mice using a flash intensity of 0.1 cd·s/m^2^. In the vehicle-treated group (gray trace), the a- and b-wave amplitudes are reduced compared to those recorded from the emixustat (purple trace) and **(** ***E*** **)-9** groups (orange trace). The quantification of the a- (Figure 6G) and b- (Figure 6H) waves over a broad range of flash intensities (0.002–100 cd·s/m^2^) showed a significant difference between the vehicle group (black) compared to the emixustat (purple) and **(** ***E*** **)-9** (orange) groups throughout the range of flash tested.

Together, these results demonstrated that the equilibrium between **(** ***E*** **)-9** and **(** ***Z*** **)-9** in the retina illuminated with white light provides structural and functional protection of the retina from damage induced by aberrant visual cycle activity like emixustat.

Finally, we asked whether orally administered **(** ***E*** **)-9** was pharmacologically active and whether its visual-cycle suppressive effect persisted as long as that of emixustat.^13,26^ To test this, 6–8-week-old mice received a single oral dose of **(** ***E*** **)-9** (5 mg/kg, 10% DMSO in soybean oil, 100 μL) in the dark. Thirty min later, the mice were illuminated for 10 min (10,000 lx), and the course of dark adaptation was followed by scotopic ERG. Unlike emixustat, whose effect on the scotopic ERG remains long-lasting after oral administration, **(** ***E*** **)-9** allowed recovery of the scotopic ERG to ∼50% of baseline within 8 h after dosing and to baseline levels within 24 h (Figure S8). These results show that **(** ***E*** **)-9** remains active after oral administration and exhibits a shorter pharmacodynamic duration than emixustat in this oral dosage followed by ERG paradigm.

## Discussion

Our results demonstrate, for the first time, that a systemically administered photoswitchable small molecule can be remotely activated by light within the intact posterior segment of an animal’s eye, establishing a new paradigm for spatiotemporal control of drug activity inside the eye. By coupling the γ-amino-α-aryl alcohol RPE65 recognition element of emixustat to an *ortho*-fluorinated azobenzene scaffold, we achieved retinal distribution of a kinetically stable *Z*-isomer that could be isomerized in situ to its more active *E*-isomer using visible light. As a result, we achieved on-demand suppression of the visual cycle in mice by simply illuminating the eye, an outcome that broadens the translatability of photopharmacology into treatments for retinal diseases.

From a therapeutic perspective, our photoswitchable RPE65 VCMs exemplify a potentially safer alternative to traditional visual cycle modulators like emixustat. Emixustat’s clinical limitations stem from continuous inhibition of RPE65 even in darkness, leading to bothersome visual effects (night blindness and delayed dark adaptation) in healthy human subjects,^26^ which translated into low patient compliance in clinical trials.^7^ With our new system, drug activity is tightly coupled to illumination: the inhibitor remains less active in the dark (*Z*-isomer) and is converted into the more active state (*E*-isomer) when light is applied. Critically, this light-induced blockade of RPE65 is lost on the time scale of hours, resulting in the recovery of physiological visual cycle activity within the same day. At the same time, the light-activated drug retained the protective efficacy of emixustat. This combination of on-demand activation and conveniently rapid offset can mitigate the dose-limiting side effects of emixustat while preserving its therapeutic benefit. Compared to other short-acting RPE65 inhibitors,^13^ our new system offers the crucial advantage of being activatable by light.

Our findings also help clarify the design trade-offs in photopharmacology for ocular applications. The earlier azobenzene-based phototherapeutics applied to visual systems relied on targeting retinal ganglion cells with intravitreal injections of thermally labile *Z*-isomers that rapidly isomerize to the *E* configuration in the dark.^3^ This strategy allows drug activity to be turned “off” automatically when illumination ceases. In those donor–acceptor azobenzene (push–pull) systems, emphasis is placed on maximizing the light-induced equilibrium shift toward the active isomer, often at the expense of *Z*-isomer kinetic stability. By contrast, our strategy requires a long-lived inactive *Z*-isomer that distributes in RPE cells after systemic administration and can be activated for on-demand RPE65 inhibition. We specifically engineered a photoswitch with a long-lived *Z* form so that a light trigger induces a sustained therapeutic effect. The *ortho*-fluorinated azobenzene scaffold is instrumental in achieving dramatically slower thermal relaxation of the *Z*-isomer (days to weeks) while still enabling efficient photoswitching with visible light. Thus, our work shifts the focus toward kinetic stability as a key design criterion for photopharmacology in living systems, highlighting how prolonging (or otherwise controlling) the lifetime of the active isomer can broaden the scope of drug targets, here, extending to an iconic enzymatic pathway within the eye.

A compelling aspect of our crystallographic analysis of the RPE65-**(** ***E*** **)-9** complex is that the azobenzene core is twisted in the enzyme’s binding pocket, deviating significantly from the characteristic planarity of this conjugated π system as it is expected to exist in solution. Mechanistically, we hypothesize that this distortion prevents deactivation through **(** ***E*** **)-9→(** ***Z*** **)-9** photoisomerization for two reasons. First, based on our crystal structure, the RPE65 cavity cannot accommodate **(** ***Z*** **)-9**. Second, we showed a dramatic reduction (∼10-fold) of the calculated n**→**π* absorption band oscillatory strength in the RPE65-**(** ***E*** **)-9** geometry compared to the geometry of **(** ***E*** **)-9** in solvent (Table S2). Such a change attenuates the n**→**π* molar absorptivity of **(** ***E*** **)-9** (Figure 3F, inset) and makes it more resistant to **(** ***E*** **)-9→(** ***Z*** **)-9** deactivation. Importantly, we acknowledge that **(** ***E*** **)-9** binds RPE65 reversibly and thus the unbound fraction of **(** ***E*** **)-9** could be photoconverted into **(** ***Z*** **)-9** in the retina.

Looking forward, two major challenges remain as opportunities for future optimization. First, we will look to increase the potency gap between **(** ***Z*** **)-9** and **(** ***E*** **)-9** from 5-fold to ∼100-fold to obtain an inactive *Z*-isomer. Achieving such an inhibitory index will likely require implementing new designs, such as incorporating steric blocking groups that only align properly in one isomer or designing different photochromic scaffolds that undergo more drastic shape changes upon isomerization. Second, augmentation of the kinetic stability of **(** ***Z*** **)-9** will be important. Although **(** ***Z*** **)-9** is far more kinetically stable than the nonfluorinated analogue **(** ***Z*** **)-7**, we observed light-independent **(** ***Z*** **)-9→(** ***E*** **)-9** conversion in vivo, eroding the fraction of drug remaining in the inactive state over time. This is why we did not pursue a standalone ERG recovery time course for systemically administered **(** ***Z*** **)-9** in this initial study, as the resulting pharmacodynamic profile would reflect an evolving (*E*)/(*Z*) mixture rather than a stable pure dark-state species. The exact cause of this isomerization remains to be determined. One obvious approach to overcome this problem is to extend the *ortho*-substitution at position 2 or additional withdrawing groups at position 4’’ in the azobenzene core (Figure 1F), which is expected to yield the most stable *Z* configuration.^16^ Finally, it is important to state that a translational limitation of the present study is that all in vivo experiments were performed in albino BALB/cJ mice, which were selected intentionally to minimize melanin as a confounding variable during this initial proof-of-concept evaluation of ocular photopharmacology, not confounded by melanin. Direct evaluation of melanin binding and performance in pigmented models will therefore be an important next step in defining the translational behavior of this class of molecules.

In conclusion, by integrating a photoswitch into an RPE65 inhibitor, we achieved reversible, highly precise spatiotemporal modulation of visual cycle activity using visible light. While challenges such as optimizing isomer potency and *Z*-isomer stability remain, our results chart a clear path toward a new class of VCMs capable of “on-demand” activation and timed restoration of rod-mediated night vision. More broadly, this work establishes that the outer retina can be precisely and noninvasively targeted by photopharmacology, opening the door to a spectrum of light-directed treatments in ophthalmology and beyond. Looking ahead, continued advances in photoswitch design and molecular targeting, guided by mechanistic insights from this study, should enable safe, reversible control of many biological targets in vivo, ultimately translating the precision of light into tangible patient benefits.

## Supplementary Material



## Notes

Detailed experimental procedures, materials, and characterization data including NMR data for all new compounds (PDF)

## Abbreviations

VCM: visual cycle modulator
AMD: age-related macular degeneration
STGD1: Stargardt disease type 1
RAL: retinal
ROL: retinol
atRAL: all-trans-retinal
11-cis-RAL: 11-cis-retinal
atRE: all-trans-retinyl ester
RPE: retinal pigment epithelium
RPE65: retinal pigment epithelium–specific protein 65 kDa
CRALBP: cellular retinaldehyde-binding protein
ERG: electroretinography
OCT: optical coherence tomography
SLO: scanning laser ophthalmoscopy
PSS: photostationary state
TDDFT: time-dependent density functional theory
