# Supplementary material for: Light-Activated RPE65 Inhibitors Enable On-Demand Visual Cycle Control
Source: J Am Chem Soc. 2026 May 19;148(21):21846–57. doi: 10.1021/jacs.6c02962 (PMC13244443; doi:10.1021/jacs.6c02962)
Supplement: Supplementary file 1 [file ja6c02962_si_001.pdf]

Supporting Information for:

Light-Activated RPE65 Inhibitors Enable On-demand Visual Cycle Control

Marco Bassetto[1,2,3*], Bowen Li[4*], Xiuyuan Chen[4], Jianye Zhang[2], Yulun Hu[4], Jordan Zaluski[4], Lauren M. Brumit[4], Preston M Willis[4], Felix Grun[5], Krzysztof Palczewski[1,2,5,6], Philip D. Kiser[1,2,3,7,#], Gregory P. Tochtrop[4,#]

[1]Department of Physiology and Biophysics, University of California, Irvine, Irvine, CA 92697, USA

[2]Gavin Herbert Eye Institute-Robert M. Brunson Center for Translational Vision Research, Department of Ophthalmology and Visual Sciences, University of California, Irvine, Irvine, CA 92697, USA

[3] Research Service, VA Long Beach Healthcare System, Long Beach, CA 90822, USA

[4]Department of Chemistry, College of Arts and Sciences, Case Western Reserve University, Cleveland, OH 44106, USA

[5]Department of Chemistry, University of California, Irvine, Irvine, CA 92697, USA

[6]Department of Molecular Biology and Biochemistry, University of California, Irvine, Irvine, CA 92697, USA

[7] Department of Clinical Pharmacy Practice, School of Pharmacy and Pharmaceutical Sciences, University of California, Irvine, Irvine, CA 92697, USA

*These authors contributed equally to these studies

# To whom correspondence should be addressed:

Marco Bassetto – orcid.org/0000-0003-0048-0203

Felix Grun – 0000-0002-1891-7285

Lauren M Brumit – orcid.org/0009-0005-3395-098X

Yulun Hu – orcid.org/ 0000-0002-2181-1182

Philip D Kiser – orcid.org/0000-0003-1184-9539

Bowen Li – orcid.org/0000-0002-9538-892X

Krzysztof Palczewski – orcid.org/0000-0002-0788-545X

Gregory P Tochtrop – orcid.org 0000-0003-2447-254X

Preston M Willis – orcid.org/0009-0003-1384-1810

Jordan Zaluski – orcid.org/0000-0002-2563-8807

Jianye Zhang – orcid.org/0000-0002-9579-1399

Table of Contents

Methods 6

Scheme S1: Synthesis of stilbene emixustat derivatives. 15

Scheme S2: Examples of unsuccessful synthesis of *ortho*-fluorinated azo-emixustat. 16

Table S1: X-ray diffraction data collection, processing, and refinement. 17

Table S2: Vertical excitation of the S1 (n→π*) and S2 (π →π*) transitions for **(*E*)-9**, **(*Z*)-9**, and RPE65-**(*E*)-9.** 18

Figure S1: Stability of **(*Z*)-7**, **(*Z*)-8**, and **(*Z*)-9** in PBS in the darkness 19

Figure S2: Stability of **(*E*)-9** solutions (1 mg/ml) in DMSO (red trace) and PBS supplemented with 10% FBS (green trace) incubated at 37°C, and DMSO solution kept frozen at - 80°C (blue trace). 20

Figure S3: Illumination with 385-400 nm light does not inhibit RPE65 isomerization in vitro. 20

The reaction mixture was supplemented with vehicle or emixustat (50 µM) and incubated for 10 min on ice either in the dark or in the presence of 385-400 nm light. 20

Figure S4: Computational modeling of **(*Z*)-9**, **(*E*)-9**, and RPE65-**(*E*)-9** absorbance spectra using various TDDFT functionals. 21

Figure S5: UV-Vis spectrum of light sources. 22

Figure S6: LC-MS/MS method for (*E*)-3 and (*E*)-9. 23

Figure S7: Effect of 10 min illumination with 405 nm LED light on dark adaptation 24

Figure S8: **(*E*)-9** is active after oral administration and a short-acting RPE65 inhibitor. 24

General Synthetic Procedure for Knoevenagel Condensation 26

General Synthetic Procedure for Catalytic Hydrogenation 26

General Synthetic Procedure for Nitrile Reduction 26

Synthetic Procedure for 2,6-Difluoronitrosobenzene 26

General Synthetic Procedure for Azobenzene Derivatives 27

Synthetic Procedure for (*E*)-3-styrylbenzaldehyde (1a) 27

Figure S9: ^{1}H NMR spectrum of (*E*)-3-styrylbenzaldehyde **(1a)** ($CDCl_3$, 500 MHz). 28

Figure S10: ^{13}C NMR spectrum of (*E*)-3-styrylbenzaldehyde **(1a)** ($CDCl_3$, 126 MHz). 28

Synthetic Procedure for (*Z*)-(2-bromovinyl)benzene 29

Synthetic Procedure for (*Z*)-3-styrylbenzaldehyde 29

Figure S11: ^{1}H NMR spectrum of (*Z*)-3-styrylbenzaldehyde **(1b)** ($CDCl_3$, 500 MHz)............................ 31

Figure S12: ^{13}C NMR spectrum of (*Z*)-3-styrylbenzaldehyde **(1b)** ($CDCl_3$, 126 MHz). 31

Synthetic Procedure for (*E*)-3-hydroxy-3-(3-styrylphenyl)propanenitrile (2a) 32

Figure S13: ^{1}H NMR spectrum of (*E*)-3-hydroxy-3-(3-styrylphenyl)propanenitrile **(2a)** ($CDCl_3$, 500 MHz). 33

Figure S14: ^{13}C NMR spectrum of (*E*)-3-hydroxy-3-(3-styrylphenyl)propanenitrile **(2a)** ($CDCl_3$, 126 MHz). 33

Synthetic Procedure for (*Z*)-3-hydroxy-3-(3-styrylphenyl)propanenitrile (2b) 34

Figure S15: ^{1}H NMR spectrum of (*Z*)-3-hydroxy-3-(3-styrylphenyl)propanenitrile **(2b)** ($CDCl_3$, 500 MHz). 35

Figure S16: ^{13}C NMR spectrum of (*Z*)-3-hydroxy-3-(3-styrylphenyl)propanenitrile **(2b)** ($CDCl_3$, 126 MHz). 35

Synthetic Procedure for (*E*)-3-amino-1-(3-styrylphenyl)propan-1-ol (3a).. 36

Figure S17: ^{1}H NMR spectrum of (*E*)-3-amino-1-(3-styrylphenyl)propan-1-ol **(3a)** ($CDCl_3$, 500 MHz).... 37

Figure S18: ^{13}C NMR spectrum of (*E*)-3-amino-1-(3-styrylphenyl)propan-1-ol **(3a)** ($CDCl_3$, 126 MHz). . 37

Synthetic Procedure for (*Z*)-3-amino-1-(3-styrylphenyl)propan-1-ol (3b).. 38

Figure S19: ^{1}H NMR spectrum of (*Z*)-3-amino-1-(3-styrylphenyl)propan-1-ol **(3b)** ($CDCl_3$, 500 MHz).... 39

Figure S20: ^{13}C NMR spectrum of (*Z*)-3-amino-1-(3-styrylphenyl)propan-1-ol **(3b)** ($CDCl_3$, 125 MHz). . 39

Synthetic Procedure for 3-(3-Nitrophenyl)-3-hydroxypropanenitrile (4a) 40

Figure S21: ^{1}H NMR spectrum of 3-(3-Nitrophenyl)-3-hydroxypropanenitrile **(4a)** ($CDCl_3$, 500 MHz). ... 41

Figure S22: ^{13}C NMR spectrum of 3-(3-Nitrophenyl)-3-hydroxypropanenitrile **(4a)** ($CDCl_3$, 126 MHz). .. 41

Synthetic Procedure for 3-(4-Fluoro-3-nitrophenyl)-3-hydroxypropanenitrile (4b) 42

Figure S23: ^{1}H NMR spectrum of 3-(4-Fluoro-3-nitrophenyl)-3-hydroxypropanenitrile **(4b)** ($CDCl_3$, 500 MHz). 42

Figure S24: ^{13}C NMR spectrum of 3-(4-Fluoro-3-nitrophenyl)-3-hydroxypropanenitrile **(4b)** ($CDCl_3$, 126 MHz). 43

Figure S25: ^{19}F NMR spectrum of 3-(4-Fluoro-3-nitrophenyl)-3-hydroxypropanenitrile **(4b)** ($CDCl_3$, 471 MHz). 43

Synthetic Procedure for 3-(3-Aminophenyl)-3-hydroxypropanenitrile (5a)..................................... 44

Figure S26: ^{1}H NMR spectrum of 3-(3-Aminophenyl)-3-hydroxypropanenitrile **(5a)** (CD_3OD, 500 MHz).45

Figure S27: ^{13}C NMR spectrum of 3-(3-Aminophenyl)-3-hydroxypropanenitrile **(5a)** (CD_3OD, 126 MHz). 45

Synthetic Procedure for 3-(3-Amino-4-fluorophenyl)-3-hydroxypropanenitrile (5b) 46

Figure S28: ^{1}H NMR spectrum of 3-(3-Amino-4-fluorophenyl)-3-hydroxypropanenitrile **(5b)** (CD_3OD, 500 MHz). 46

Figure S29: ^{13}C NMR spectrum of 3-(3-Amino-4-fluorophenyl)-3-hydroxypropanenitrile **(5b)** (CD_3OD, 126 MHz). 47

Figure S30: ^{19}F NMR spectrum of 3-(3-Amino-4-fluorophenyl)-3-hydroxypropanenitrile **(5b)** (CD_3OD, 471 MHz). 47

Synthetic Procedure for 3-Amino-1-(3-aminophenyl)propan-1-ol (6a) 48

Figure S31: ^{1}H NMR spectrum of 3-Amino-1-(3-aminophenyl)propan-1-ol **(6a)** (CD_3OD, 500 MHz)...... 49

Figure S32: ^{13}C NMR spectrum of 3-Amino-1-(3-aminophenyl)propan-1-ol **(6a)** (CD_3OD, 126 MHz)..... 49

Synthetic Procedure for 3-Amino-1-(3-amino-4-fluorophenyl)propan-1-ol (6b) 50

Figure S33: ^{1}H NMR spectrum of 3-Amino-1-(3-amino-4-fluorophenyl)propan-1-ol **(6b)** (CD_3OD, 500 MHz). 50

Figure S34: ^{13}C NMR spectrum of 3-Amino-1-(3-amino-4-fluorophenyl)propan-1-ol **(6b)** (CD_3OD, 126 MHz). 51

Figure S35: ^{19}F NMR spectrum of 3-Amino-1-(3-amino-4-fluorophenyl)propan-1-ol **(6b)** (CD_3OD, 471 MHz). 51

Synthetic Procedure for (*E*)-3-Amino-1-(3-(phenyldiazenyl)phenyl)propan-1-ol [(*E*)-7] 52

Figure S36: ^{1}H NMR spectrum of (*E*)-3-Amino-1-(3-(phenyldiazenyl)phenyl)propan-1-ol [**(*E*)-7**] (CD_3OD, 500 MHz). 53

Figure S37: ^{13}C NMR spectrum of (*E*)-3-Amino-1-(3-(phenyldiazenyl)phenyl)propan-1-ol [**(*E*)-7**] (CD_3OD, 126 MHz). 53

Synthetic Procedure for (*E*)-3-Amino-1-(3-((2,6-difluorophenyl)diazenyl)phenyl)propan-1-ol [(*E*)-8] 54

Figure S38: ^{1}H NMR spectrum of (*E*)-3-Amino-1-(3-((2,6-difluorophenyl)diazenyl)phenyl)propan-1-ol [**(*E*)-8**] (CD_3OD, 500 MHz). 54

Figure S39: ^{13}C NMR spectrum of (*E*)-3-Amino-1-(3-((2,6-difluorophenyl)diazenyl)phenyl)propan-1-ol [(***E***)-**8**] (CD_3OD, 126 MHz). 55

Figure S40: ^{19}F NMR spectrum of (*E*)-3-Amino-1-(3-((2,6-difluorophenyl)diazenyl)phenyl)propan-1-ol **[(*E*)-8]** (CD_3OD, 471 MHz). 55

Synthetic Procedure for (*E*)-3-Amino-1-(3-((2,6-difluorophenyl)diazenyl)-4-fluorophenyl)propan-1-ol [(*E*)-9] 56

Figure S41: ^{1}H NMR spectrum of (*E*)-3-Amino-1-(3-((2,6-difluorophenyl)diazenyl)-4-fluorophenyl)propan-1-ol **[(*E*)-9]** (CD_3OD, 500 MHz). 56

Figure S42: ^{13}C NMR spectrum of (*E*)-3-Amino-1-(3-((2,6-difluorophenyl)diazenyl)-4-fluorophenyl)propan-1-ol [(***E***)-**9**] (CD_3OD, 126 MHz). 57

Figure S43: ^{19}F NMR spectrum of (*E*)-3-Amino-1-(3-((2,6-difluorophenyl)diazenyl)-4-fluorophenyl)propan-1-ol **[(*E*)-3F]** (CD_3OD, 471 MHz). 57

References 58

Detailed Description of Experimental Contributions 59

Methods

General Experimental Details for Chemical Synthesis

All reactions were performed in oven-dried glassware, under an inert atmosphere with exclusion of moisture from reagents and solvents. Reagents were used as supplied. Liquid chromatography was performed using flash chromatography on silica gel (230-400 mesh), on a CombiFlash NextGen 300+ system, using eluting solvent (reported as a V:V-ratio mixture). Analytical thin layer chromatography (TLC) was performed on 0.25 mm glass-backed Silicycle 60 F254 plates. Visualization of the developed chromatogram was accomplished with UV light (254 nm). High resolution mass spectra were acquired on a Waters (Micromass) LCT Premier-TOF mass spectrometer. ^{1}H and ^{13}C-NMR spectra were recorded on a Bruker Ascend Avance III HDTM spectrometer operating at MHz, 500 MHz, Chemical shifts reported in δ units (part per million (ppm)) with reference to TMS or the residual solvent peak. NMR data are presented in the following order: chemical shift, peak multiplicity (s = singlet, bs = broad singlet, d = doublet, t = triplet, q = quartet, dd = doublet of doublets, m = multiplet), coupling constant (in Hz). Detailed procedures and analytical data can be found in Supplemental Information.

Procedures for HPLC purification and Analysis of synthetic compounds. Normal phase analytical high performance liquid chromatography (HPLC) was performed on Agilent 1260 series system. The separation was performed on an Agilent Zorbax Sil (5 μm, 250 mm × 4.6 mm) column using a mobile phase consisting of hexanes (A) and ethyl acetate (B) at a flow rate of 1.4 mL/min by isocratic elution by 90% A for 25 min. Reverse phase analytical high performance liquid chromatography (HPLC) was performed on Agilent 1200 series system. The separation was performed on Shimadzu Premier C18 (5 μm, 100 mm × 4.6 mm) column using a mobile phase consisting of 0.1% formic acid in water (A) and 0.1% formic acid in methanol (B) at a flow rate of 1 mL/min, and the mobile-phase gradients and time course were as follows: 0-17 min from 100% A to 50% A, 17-18 min 50% A. Preparative high performance liquid chromatography (HPLC) was performed on Shimadzu LC-20AP instrument with SPD-M20A UV/Vis photodiode array detector and FRC-10A spectrofluorometric detector. The separation was performed on Agilent Prep C18 (10 μm, 250 mm × 21.2 mm) column using a mobile phase consisting of 0.1% formic acid in water (A) and 0.1% formic acid in methanol (B) at a flow rate of 10 mL/min, and the mobile-phase gradients and time course were as follows: 0-30 min from 100% A to 50% A, 30-45 min from 50% A to 100% A.

Determination of the photostationary states (PSS) of azo-emixustat derivative by irradiation with different wavelengths of light and in white light of different intensities. Photoisomerization was performed using Rayonet RPR 100 photoreactor with 254 nm, 300 nm, 350 nm, 419 nm lamp; Afanty 30W 385-400 nm diode; 30 W 460 nm LED light (Amazon); DarkDawn V5 5W 395 nm violet flashlight;

Newport 67005 xenon lamp with Newport 69920 power supply through Life Pixel 82 mm diameter external mounted visible bandpass filter, and DarkDawn V5 5W 395 nm violet flashlight. Light intensity was determined by FISHR RS232 light meter. 1 mg/mL **(*E*)-7**, **(*E*)-8**, or **(*E*)-9** solution was prepared in methanol and transferred to a 3 cm × 1 cm× 1 cm quartz cuvette. The cuvette was sealed with cap and parafilm and irradiated with various wavelengths of light via Rayonet RPR 100 photoreactor (460 nm light source was provided by a 30 W 460 nm LED light with 18 beads purchased from Amazon). After 1 hour, the solution was transferred to an amber color HPLC sample vial and analyzed by Agilent 1200 series reverse phase analytical HPLC system. The separation was performed on Shimadzu Premier C18 (5 µm, 100 mm × 4.6 mm) column using a mobile phase consisting of 0.1% formic acid in water (A) and 0.1% formic acid in methanol (B) at a flow rate of 1 mL/min, and the mobile-phase gradients and time course were as follows: 0-17 min from 100% A to 50% A, 17-18 min 50%A. Peaks were detected by monitoring absorbance at 254 nm (ref 600 nm).

The PSS of solution of **(*E*)-9** (1 mg/mL) in white light was determined under illumination with white light of different intensities. **(*E*)-9** solution was prepared in methanol and transferred to a 3 cm × 1 cm× 1 cm quartz cuvette. The cuvette was sealed with cap and parafilm and irradiated with 500-5000 nm light via Rayonet RPR 100 photoreactor. After 1 hour, the **(*Z*)-9** was purified by Shimadzu LC-20AP HPLC system with SPD-M20A UV/Vis photodiode array detector and FRC-10A spectrofluorometric detector. The separation was performed as above. The solution was then transferred into 2 mL vials and irradiated by Newport 67005 xenon lamp with Newport 69920 power supply through Life Pixel 82 mm diameter external mounted visible bandpass filter or irradiated by DarkDawn V5 5W 395 nm violet flashlight under various light intensities (measured by FISHR RS232 light meter) for a given time and analyzed by Agilent 1200 series reverse phase analytical HPLC system as above.

Kinetic stability test of azo-emixustat derivative *Z*-isomers. 1 mg/mL **(*Z*)-7**, **(*Z*)-8**, or **(*Z*)-9** solution were prepared in methanol and transferred to a 3 cm × 1 cm× 1 cm quartz cuvette. The cuvette was sealed with cap and parafilm and irradiated with 300 nm light via Rayonet RPR 100 photoreactor. After 1 hour, the *Z* isomers were purified by Shimadzu LC-20AP HPLC system with SPD-M20A UV/Vis photodiode array detector and FRC-10A spectrofluorometric detector. The separation was performed on Agilent Prep C18 (10 µm, 250 mm × 21.2 mm) column using a mobile phase consisting of 0.1% formic acid in water (A) and 0.1% formic acid in methanol (B) at a flow rate of 10 ml/min, and the mobile-phase gradients and time course were as follows: 0-30 min from 100% A to 50%A, 30-45 min from 50%A to 100% A. Peaks were detected by monitoring absorbance at 254 nm (ref 600 nm). The solvent was removed under reduced pressure, and purified (*Z*)-azo-emixustat was redissolved in 10 ml PBS buffer (phosphate buffered saline, pH = 7.4), incubated under 37°C for a given time and analyzed by Agilent

1200 series reverse phase analytical HPLC system. The separation was performed on Shimadzu Premier C18 (5 μm, 100 mm × 4.6 mm) column using a mobile phase consisting of 0.1% formic acid in water (A) and 0.1% formic acid in methanol (B) at a flow rate of 1 ml/min, and the mobile-phase gradients and time course were as follows: 0-17 min from 100% A to 50% A, 17-18 min 50%A. Peaks were detected by monitoring absorbance at 254 nm (ref 600 nm).

Plotting % (*Z*)-isomer versus time for each individual replicate gave the rate constant *k* by using the first-order kinetic equation $[Z] = [Z]_0 e^{-kt}$ and the corresponding thermal half-life ($\tau_{1/2}$) using the equation $\tau_{1/2} = \ln(2)/k$. The fitting data obtained by each individual replicate were averaged and results are reported the legend of **Fig. 2**..

RPE microsomal preparations. Bovine RPE microsomes were isolated from RPE homogenates by differential centrifugation as previously described [1]. The resulting microsomal precipitate was resuspended in 10 mM Bis-Tris propane/HCl buffer, pH 7.4, to achieve a total protein concentration of approximately 5 mg/mL. Then, the mixture was placed into a quartz cuvette and irradiated for 6 min at 4 °C with a ChromatoUVE transilluminator (model TM-15; UVP) to eliminate residual retinoids. After irradiation, DTT was added to the RPE microsomal mixture to achieve a final concentration of 5 mM.

Experimental Procedure of Transformation, Growth, and Purification of CRALBP. A human 6-His-tagged CRALBP-encoding plasmid (pET19b-CRALBP) was transformed by heat shock into BL-21, which was then grown in LB media at 37 °C until an OD 600 of 0.5 was obtained. CRALBP expression was then induced with the addition of IPTG (0.1 mM final concentration) followed by culturing for 14 h at 22 °C with shaking (200 rpm). Cells were pelleted by centrifugation (10,000g at 4 °C for 15 min), resuspended in buffer, and then subjected to nitrogen cavitation (2 × 900 psi) with stirring. Lysed cells were centrifuged (30,000g at 4 °C for 30 min), and the supernatant was collected. CRALBP was purified by Ni-sepharose chromatography and de-salted on a HiPrep 26/10 column. Fractions were monitored by 280 nm absorbance and collected. Protein concentration was determined by the BCA assay.

In vitro RPE65 activity assay. Under dim red-light illumination, synthesized compounds and emixustat (2 μL in DMF, with the final concentration ranging between 0.1 and 10 μM) were added to 10 mM Bis-Tris propane/HCl buffer, pH 7.4, containing 150 μg RPE microsomes, 1% BSA, 1 mM disodium pyrophosphate, and 20 μM apo-CRALBP. The resulting mixture was preincubated at room temperature for 5 min. Then, all-*trans*-retinol (1 μL in DMF, at a final concentration of 20 μM) was added. The resulting mixture was incubated at 37 °C for 1 h. The reaction was quenched by adding 300 μL methanol, and products were extracted with 300 μL hexanes. Production of 11-*cis*-retinol was quantified

by normal-phase HPLC with 10% (v/v) ethyl acetate in hexanes as the eluent at a flow rate of 1.4 mL/min. Retinoids were detected by monitoring absorbance at 325 nm and quantified based on a standard curve representing the relationship between the amount of synthetic 11-*cis*-retinol standard and the area under the corresponding chromatographic peak.

The experiment investigating the photoisomerization of **(*Z*)-9** was carried out as above with the difference that the reaction mixture supplemented with **(*Z*)-9** was irradiated by 385-400 nm light for 10 min on ice, then all-*trans* retinol (5 mM in DMF, 0.5 μL) was added and the resulting mixture was incubated at 37 °C for 1 h in the dark and analyzed as above.IC_{50} and relative SD were obtained by fitting the results from each inhibitor using the [Inhibitor] vs normalized response – variable slope function of GraphPad Prism software.

The same experimental setup was used to study the impact of 385-400 nm light on RPE65 isomerization activity, with the difference that an aliquot of the reaction mixture was illuminated for 10 min with 405 nm LED light. Specifically, this aliquot of reaction mixture was placed in a quartz cuvette laid on ice, and the LED light was placed in contact with the cuvette. In the aliquot of reaction mixture containing emixustat control, the concentration of the test compound was 50 μM. Finally, all samples were processed as above.

RPE65 crystallization and structure determination

Crystals of RPE65 in the complex with **(*E*)-9** were obtained using previously described procedures. Briefly, isolated bovine RPE membranes were incubated with 10 mM **(*E*)-9** (delivered in DMF) for 15 min before solubilization with 24 mM hexaethylene glycol mono-octyl ether (C_8E_6). After anion-exchange chromatography, purified RPE65 was concentrated to 10 mg/mL and **(*E*)-9** was added again to a concentration of 10 mM before crystallization. Crystals were grown by the hanging-drop vapor diffusion method by mixing 2 μL of a 10 mg/mL RPE65 sample with 2 μL of crystallization solution consisting of 30% v/v PEG 200, 100 mM Tris base/Hydrochloric acid pH 8.5, and 200 mM (ammonium phosphate dibasic. The drops were incubated over 500 μL of well solution consisting of 100 mM 2-(cyclohexylamino)ethanesulfonic acid–NaOH, pH 9.5, containing 40% (v/v) polyethylene glycol 300 and 200 mM NaCl. After incubation for 1–2 weeks at 8 °C, crystals of ∼100 × 100 × 300 μm^3 in size were observed. Mature crystals were harvested directly into liquid nitrogen for X-ray data collection. X-ray diffraction data were collected at the SSRL 12-1 beamline. Data were processed using XDS [2], and the initial model was obtained by direct refinement using published RPE65 coordinates in which ligands had been removed (PDB accession code: 4RSC). The structure was refined by alternating reciprocal space refinement in REFMAC and manual building and adjustments in Coot [3]. Ligand coordinates and

geometry dictionary files were generated using the Grade server (http://grade.globalphasing.org/cgi-bin/grade/server). The models were validated using MolProbity [4] and the wwPDB validation server [5].

TDDFT calculations of (*Z*)-9, (*E*)-9, and RPE65 in a complex with (*E*)-9

The X-ray crystal structure of RPE65-**(*E*)-9** was protonated with OpenBabel v3.1.1[6] and used as the starting structure for all RPE65-**(*E*)-9** calculations. Initial models of (E)-9 and (Z)-9 were generated from the RPE65-**(*E*)-9** scaffold, pre-optimized with XTB v6.7.1[7] (ALPB water), and subjected to a conformational search using CREST[8] v3.0.2. All resultant structures were confirmed as true minimum by harmonic frequency analysis. The resulting candidate **(*E*)-9** and **(*Z*)-9** structures were then optimized with ORCA[9] v6.0.1 at B3LYP-D3BJ/def2-TZVP/SMD(water) to determine the lowest energy **(*E*)-9** and **(*Z*)-9** structure, confirmed of no imaginary frequencies, and were then taken forward. Electronic absorption spectra for each isomer were computed by TDDFT (nroots=50, TDA off) at B3LYP/def2-TZVPD/SMD(water), CAM-B3LYP/def2-TZVPD/SMD(water), PBE0/def2-TZVPD/SMD(water), and ωB97X/def2-TZVPD/SMD(water) levels following established procedures.[10] Stick spectra were broadened with a Gaussian width of 0.5 eV and plotted in Origin 2024b.

Study approvals. All animal procedures were approved by the Institutional Animal Care and Use Committees (IACUC) at the University of California Irvine and the Tibor Rubin VA Long Beach Medical Center. All experimental protocols were conducted following the *NIH Guide for the Care and Use of Laboratory Animals*, the recommendations of the American Veterinary Medical Association Panel on Euthanasia, and the Association for Research in Vision and Ophthalmology (ARVO) *Statement for the Use of Animals in Ophthalmic and Visual Research.*

Quantification and statistical analysis. No statistical methods were applied to predetermine sample size. The ages of mice used for experiments are given in figure legends. The experiments were not randomized, and the investigators were not blinded to allocation prior to data analysis. Statistical analyses and graph generation were carried out using GraphPad Prism. Statistical methods and details of descriptive statistics are provided in the figure legends. A p value of less than 0.05 was considered significant.

Sex as a biological variable. Our study examined male and female animals, and similar findings were reported for both sexes.

Animal husbandry. BALB/cJ mice (Jackson, strain #000651) were housed in a standard 12/12-h light/dark cycle environment, fed a standard soy protein-free rodent chow diet (Envigo Teklad 2020X),

provided water *ad libitum*, and housed in plastic cages with standard corncob rodent bedding and 6 g nestlets (Ancare). The spectral information of the white fluorescent lights in our vivarium is shown in **Fig. S3**.

Assessment of rod dark adaptation by ERG.

The scotopic ERG was measured as follows: mice were anesthetized by isoflurane inhalation and their pupils were dilated with topical administration of 1% tropicamide ophthalmic solution (Akorn; 17478-102-12) and 10% phenylephrine ophthalmic solution (MWI Animal Health #054243), followed by 0.3% hypromellose (Akorn; 9050-1) to maintain corneal hydration. Anesthetized mice were placed on a heated pad set to 37 °C to prevent hypothermia. ERG responses were measured using a Diagnosys Celeris rodent-ERG device (Diagnosys LCC, Lowell, MA, USA). Ocular stimulator electrodes were placed on the corneas, the reference electrode was positioned subdermally between the ears, and a ground electrode was placed in the rear leg. The eyes were subjected to a green-light stimulus (peak emission 544 nm, bandwidth ~160 nm) of 0.1 cd · s/m^2, which exclusively excites rod photoresponses. The responses obtained from 5 consecutive stimuli with an inter-stimulus interval of 30 s were averaged, and the a- and b-wave amplitudes were acquired from the averaged ERG waveform. Data were analyzed with Espion V6 software (Diagnosys LLC).

For the assessment of RPE65 target engagement, dark-adapted BALB/cJ mice were subjected to a 10 min, 10,000 lux photobleach with white LED light and then received a single IP injection of either vehicle (DMSO) or test compounds (1, 5, or 10 mg/kg) in the dark followed by 2 h of rearing in darkness to allow rod photoreceptors to recover their sensitivity.

For the assessment of the effect of violet LED light on dark adaptation, dark-adapted BALB/cJ mice were subjected to a 10 min, 10,000 lux photobleach with white LED light or 3000 lux violet LED light photobleach. The course of dark adaptation was monitored over 1-4 h as shown in **Fig. S4**.

For the assessment of the oral bioavailability of **(*E*)-9**, dark-adapted BALB/cJ mice were subjected to a 10 min, 10,000 lux photobleach with white LED light and then received a single oral administration of either vehicle (DMSO) or test compounds (emixustat or **(*E*)-9**) in the dark. The oral bolus was formulated as a 10% DMSO emulsion in soybean oil with final test compound concentration of 5 mg/kg) and it was administered in 100 µL per mice. Next, mice were reared in darkness for 2 h to allow rod photoreceptors to recover their sensitivity.

HPLC analysis of retinoid extracts from whole mouse eye homogenates. For retinoid analysis, both eyes from each animal were homogenized in 1 mL of 10 mM sodium phosphate buffer, pH 8, containing 50% v/v methanol (Sigma–Aldrich; 34860-1L-R) and 100 mM hydroxylamine, pH = 8 (Sigma-Aldrich; 159417-100G). After a 15 min incubation at room temperature, 2 mL of 3 M sodium chloride were added to the homogenate. The resulting sample was extracted twice with 3 mL of ethyl acetate (Fisher Scientific; E195-4). The combined organic phase was dried *in vacuo* and reconstituted in 450 µL hexane. Retinoids extracts (100 µL) were analyzed with an Agilent 1260 Infinity II HPLC system equipped with a diode array detector (DAD) and a Zorbax Sil column (5 µm; 4.6 mm × 250 mm; Agilent Technologies). The mobile phase consisted of 0.6% v/v ethyl acetate in hexanes (Fisher Scientific; H302-4) flowing at 1.4 mL/min for 17 min followed by a step increase to 10% v/v ethyl acetate in hexanes flowing at 1.4 mL/min for an additional 25 min. Retinoids were detected by monitoring absorbance at 325 nm and 360 nm with continuous spectral recording. Peaks were identified based on their absorbance spectra and retention times relative to authentic standards. Absolute quantification of retinoids was conducted by peak integration with reference to authentic retinoid standards.

Functional assessment of (*Z*)-9→(*E*)-9 photoisomerization in vivo by retinoid analysis

6-8 weeks-old BALB/cJ mice were dark adapted overnight. All the procedures that followed were carried out under a dim red LED light. The mice received one of the following treatments: either a single 50 µL IP injection of vehicle (DMSO) or test compounds (**(*Z*)-9** or **(*E*)-9**, 5 mg/kg) and an immediate exposure to 405 nm or 630 nm LED light for 5 min; or a 5 min exposure to 405 nm or 630 nm LED light and then an IP injection of vehicle or test compounds (**(*Z*)-9** or **(*E*)-9**, 5 mg/kg). After these procedures, the mice were dark adapted for 8 h followed by euthanasia and collection of the eyes for retinoid analysis.

Sample preparation for quantitative assessment of (*Z*)-9→(*E*)-9 photoisomerization in vivo by LC-MS/MS

6-8 weeks-old BALB/cJ mice were dark adapted overnight. All the procedures that followed were carried out under a dim red LED light. The mice received a single IP injection of **(*Z*)-9** (5 mg/kg, 50 µL) dissolved in DMSO in the dark. 30 min later, the mice were exposed to either a 405 nm or a 630 nm LED light for 10 min. Next, the mice were euthanized and both eyes were collected in a single plastic test tube, one tube for each animal, that was wrapped in aluminum foil and snap frozen in liquid nitrogen. A separate group of mice that received the same treatment but without exposing the animals to any light source was included to determine the baseline thermal isomerization of **(*Z*)-9→(*E*)-9**. Next, the eyes were homogenized in 600 µL of 50% methanol in water. These homogenates were spun for 15 min at 13,500 g and 4 °C. The supernatant was collected and transferred to a test tube for LC-MS/MS.

Quantification of (*Z*)-9 and (*E*)-9 by mass LC-MS/MS

An Agilent 1290 UPLC was used to separate the **(*Z*)-9** and **(*E*)-9** azobenzene emixustat derivatives as well as the internal standards stilbene emixustat derivatives **(*Z*)-3** and **(*E*)-3**. The chromatography method was initially developed with reverse-phase chromatography (Kinetex 2.6 µm Polar C18 100 Å, LC Column 100 × 2.1 mm) using the Agilent 1290 platform. Mobile phase A and B were 0.1% formic acid in water and 0.1% formic acid in acetonitrile, respectively. The gradient was: 0–30% B over 0–6 min, hold at 30% B for 6–10 min, then return to 0% B for 10–15 min; the flow rate was 0.2 mL/min. Injection volume was 10 µL. Observed retention times were: 8.5 min for **(*Z*)-9**, 9.1 min for **(*E*)-9**, 9.5 min for **(*Z*)-3**, and 9.9 min for **(*E*)-3**. For the MS analysis, an Agilent 6475 Triple Quadrupole LC/MS was used with instrument optimization to identify abundant and selective multiple reaction monitoring (MRM) transitions for the analyte and internal standard. We used Agilent MassHunter Optimizer to perform direct-infusion product-ion scans of the azobenzene and stilbene emixustat derivatives, sweeping fragmentor voltage and collision energy to locate the most intense, selective precursor/product pairs. The most abundant fragments were determined by collision-induced dissociation (CID) experiments. Samples were infused in positive ionization mode, and fragmentation was performed with a collision energy of 14 eV. To obtain optimized MRM transitions, standard solutions were directly infused into the mass spectrometer, and the most abundant daughter ions were determined as a function of ionization energy. Based on ion intensity, as shown in (**Fig. S6**), the selected transitions were: **(*Z*)-9** and **(*E*)-9** m/z 309.1/263.0 (collision energy 14 eV); **(*Z*)-3** and **(*E*)-3** (internal standard) m/z 253.2/30.2 (collision energy 14 eV).

Induction of retinal phototoxicity in mice

6-8-week-old BALBc/J mice were dark-adapted for 24 h prior to the experiment, and all drug administration procedures were performed under dim red light. Animals were administered 50 µL of vehicle or test compounds (emixustat or **(*E*)-9**) dissolved in DMSO at a dose of 10 mg/kg in DMSO vehicle by IP injection. Pupils were dilated by the application of a drop of 1% atropine sulfate to the eye (Ophthalmics Inc.). The mice were placed in a cage with a light-reflective white coating. Each cage was divided into four equal compartments with transparent Plexiglas separators. To prevent grouping and consequent light shielding, single animals were placed into their own compartment for the duration of the experiment. Mice were exposed to broad-spectrum 15,000 lux white light emitted by clusters of LEDs placed on top of each white cage for 8 h. The animals had full access to food and water during light exposure. During the light exposure, atropine sulfate was applied to mouse eyes every 2 h to maintain mydriasis. After light exposure, animals were returned to their normal cages and kept under standard lighting conditions for one week before SLO and OCT imaging and ERG.

In vivo ERG scotopic intensity-response measurements after light damage

One week after induction of retinal damage induced by light, mice were dark-adapted overnight for rod ERG recordings. All procedures were performed like described above for the measurement of rod dark-adaptation with the difference that several intensities of green-light stimulus were assessed (0.002 to 100 cd · s/m^2). The responses obtained from consecutive stimuli were averaged, and the a- and b-wave amplitudes were acquired from the averaged ERG waveform. Data were analyzed with Espion V6 software (Diagnosys LLC).

In vivo retinal imaging. Mice were anesthetized by an IP injection of ketamine (100 mg/kg) and xylazine (8.75 mg/kg) and pupils were dilated with 1% tropicamide and 10% phenylephrine before imaging. Ultrahigh-resolution spectral domain OCT (Bioptigen, Research Triangle Park, NC) was performed for cross-sectional imaging of mouse retinas as described previously[11]. Briefly, five frames of OCT images were first acquired in the B-mode in two orthogonal directions and then averaged. Images were then analyzed using ImageJ software. Briefly, for each animal, the images collected from each eye were used to measure the thickness of the outer nuclear layer (ONL) in the four retinal quadrants at 0.5 mm from the optic nerve head. The values from the right and left eye of the same animal were averaged. During the same session, the fundus was imaged by scanning laser ophthalmoscopy (SLO) (Heidelberg Engineering, Heidelberg, Germany) in autofluorescence mode as previously described [11].

Histology

The enucleated mouse eyes were kept in Hartman's fixative (Millipore Sigma) for 24 h at RT, transferred to 70% ethanol, and embedded in paraffin. Sagittally cut 6-μm paraffin sections spanning the optic nerve head (ONH) were stained with hematoxylin and eosin (H&E) and imaged with light microscopy with BZ-X800 (Keyence) instruments. Manual counting of photoreceptor nuclei per row, every 500 μm starting starting from the edge of the optic nerve head along both superior and inferior directions, was performed manually. Average values of ONL nuclei counts for each animal group represent data obtained from 6 eyes.

Schemes

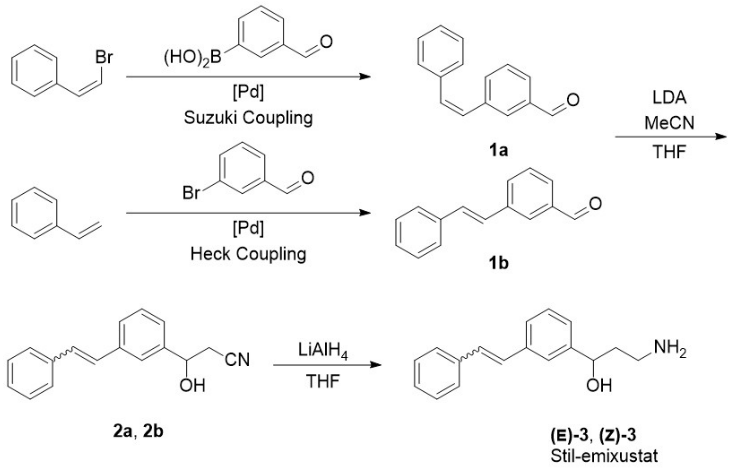

Scheme S1: Synthesis of stilbene emixustat derivatives.

(A)

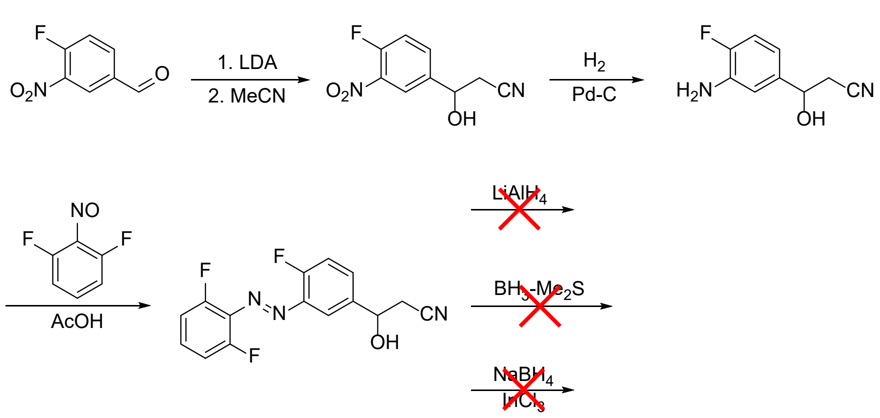

(B)

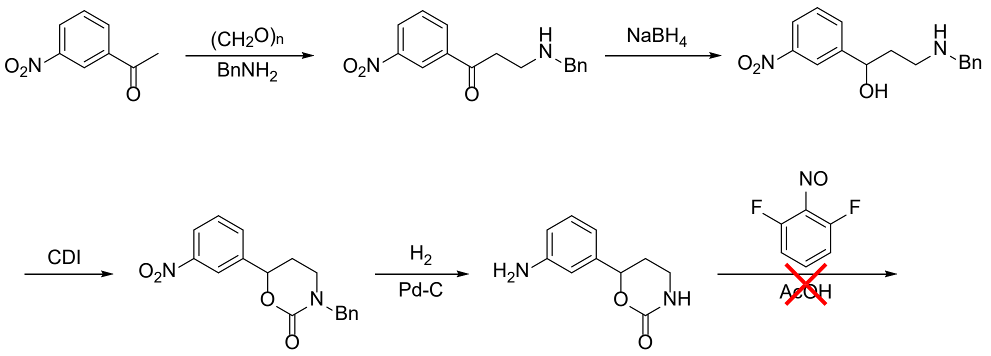

(C)

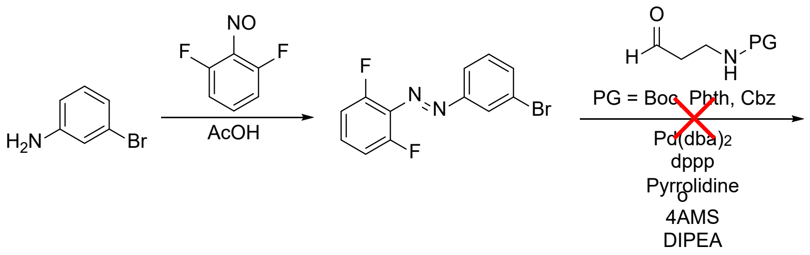

Scheme S2: Examples of unsuccessful synthesis of *ortho*-fluorinated azo-emixustat.

(A) Classic synthetic route. **(B)** Cyclic carbamate protection. **(C)** Enamine-based Heck coupling (EBHC).

Supplementary Tables

Data collection and processing	
Crystal	RPE65-**(*E*)-9** ∇
X-ray source	SSRL 12-1
Wavelength (Å)	0.979460
Space group	$P6_5$
Unit cell lengths (Å)	a = 175.72, c = 86.59
Resolution (Å)	50.0 – 1.75 (1.85 – 1.75)†
Unique reflections	153,073 (23,417)
Multiplicity	13.1 (8.4)
Completeness (%)	100 (100)
$<I/\sigma I>$	14.75 (0.56)
$R_{merge}I$ (%)	10.8 (347.8)
$CC_{1/2}$ (%)	99.9 (21.8)
Wilson *B* factor (Å^2)	40
Resolution (Å)	47.9 – 1.75
No reflections	145,313 (7,758)‡
R_{work}/R_{free} (%)	14.8/18.1
No atoms	9,246
Protein	8,184
Metal	2 FE2
Water	980
Ligand	44 **(*E*)-9**, 36 PLM
<*B*-factor> (Å^2)	42.6
Protein	40.7
Metal	32.2
Water	56.6
Ligand	66.5 **(*E*)-9**, 57.1 PLM
RMS deviations	
Bond lengths (Å)	0.004
Bond angles (º)	0.934
Ramachandran plot (% favored/outliers)*	97.2/0
All-atom clashscore*	2.15 (99th percentile)
PDB accession code	pdb_00009oa5

Table S1: X-ray diffraction data collection, processing, and refinement.

∇ Final data set obtained by merging data from two isomorphous crystals

† Values in parentheses are for the highest resolution shell of data

‡ Value in parentheses indicates the number of reflections used for cross-validation

* Evaluated using Molprobity [4]

Compound	Functional	pi-pi* v(max) (nm)	Osc. Strength	Transition Dipole Strength (D^2)	n-pi* v(max) (nm)	Osc. Strength	Transition Dipole Strength (D^2)
**(*E*)-9**	B3LYP	359.8	0.7038	53.8681	497.3	0.0310	3.2761
**(*Z*)-9**	B3LYP	343.7	0.0174	1.2727	457.7	0.0589	5.7375
RPE65-**(*E*)-9**	B3LYP	344.5	0.2856	20.9209	506.2	0.0084	0.9058
**(*E*)-9**	CAM-B3LYP	327.9	0.9269	64.6435	467.7	0.0249	2.4815
**(*Z*)-9**	CAM-B3LYP	288.6	0.0481	2.9492	438.6	0.0481	3.8453
RPE65-**(*E*)-9**	CAM-B3LYP	298.8	0.6486	41.2223	480.1	0.0041	0.4186
**(*E*)-9**	PBE0	348.9	0.7919	58.7664	494.4	0.0278	2.9188
**(*Z*)-9**	PBE0	327.5	0.0220	1.5331	451.0	0.0524	5.0250
RPE65-**(*E*)-9**	PBE0	329.3	0.3686	25.8173	504.6	0.0066	0.7081
**(*E*)-9**	wB97x	314.2	0.9442	63.1040	460.5	0.0204	2.0008
**(*Z*)-9**	wB97x	269.9	0.0807	4.6322	429.9	0.0340	3.1133
RPE65-**(*E*)-9**	wB97x	282.4	0.7440	44.6877	472.0	0.0027	0.2695

Table S2: Vertical excitation of the S1 (n→π*) and S2 (π →π*) transitions for **(*E*)-9**, **(*Z*)-9**, and RPE65-**(*E*)-9.**

Calculated using the respective functional at the def2-TZVPD/SMD(Water) level of theory.

Supplementary Figures

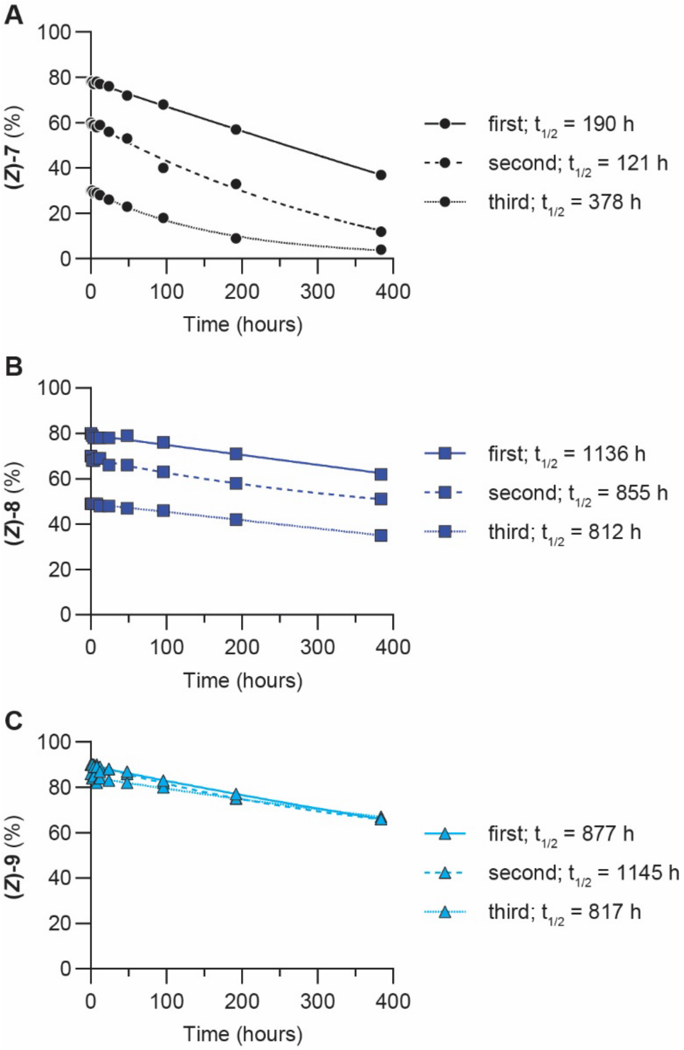

Figure S1: Stability of **(*Z*)-7**, **(*Z*)-8**, and **(*Z*)-9** in PBS in the darkness.

(A) Individual replicates of **(*Z*)-7**. **(B)** Individual replicates of **(*Z*)-8**. **(C)** Individual replicates of **(*Z*)-9**. The results from individual replicates were fit with a one-phase decay equation using Prism GraphPad software with the plateau constrained to the value of 0.

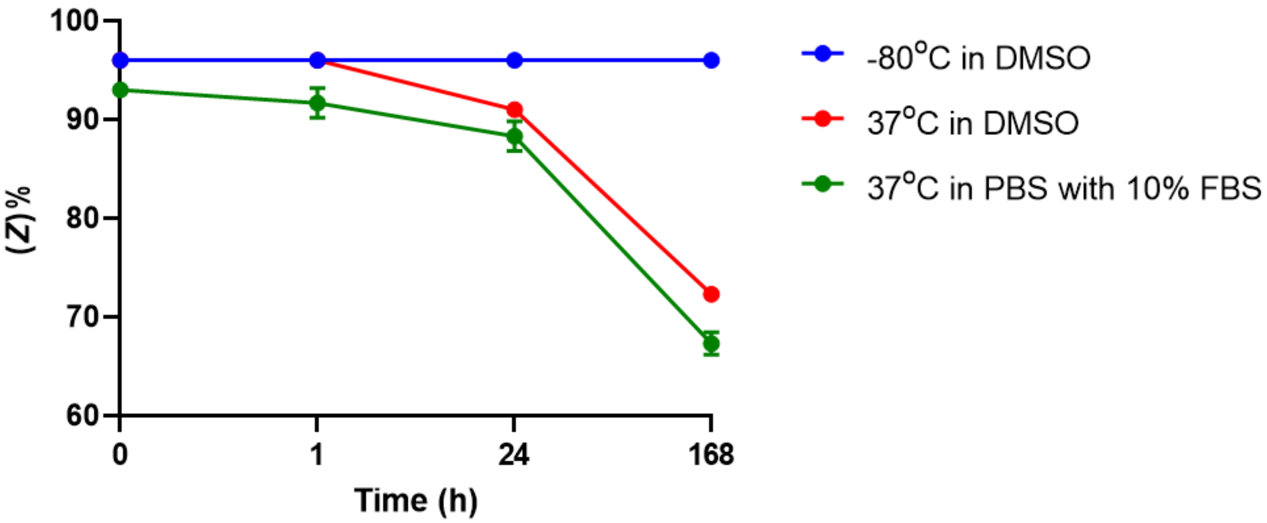

Figure S2: Stability of ***(E)*-9** solutions (1 mg/ml) in DMSO (red trace) and PBS supplemented with 10% FBS (green trace) incubated at 37°C, and DMSO solution kept frozen at - 80°C (blue trace).

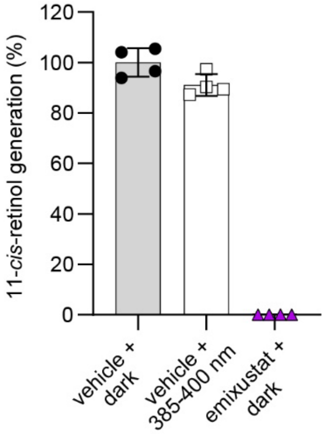

Figure S3: Illumination with 385-400 nm light does not inhibit RPE65 isomerization in vitro.

The reaction mixture was supplemented with vehicle or emixustat (50 µM) and incubated for 10 min on ice either in the dark or in the presence of 385-400 nm light. Data represent mean ± SD, each data point corresponds to a technical replicate.

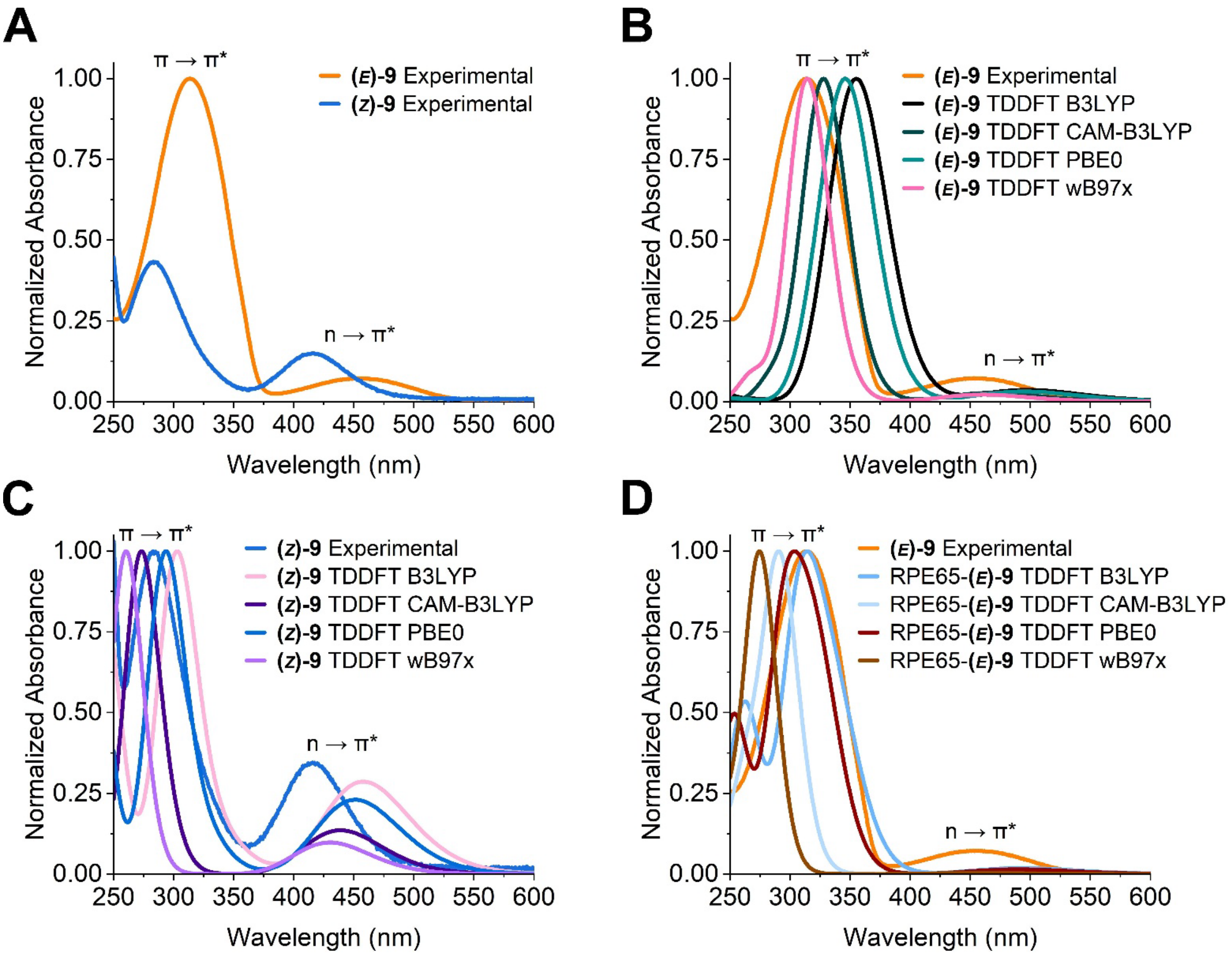

Figure S4: Computational modeling of **(*Z*)-9**, **(*E*)-9**, and RPE65-**(*E*)-9** absorbance spectra using various TDDFT functionals.

(A) The **(*Z*)-9→(*E*)-9** photoisomerization results in a pronounced attenuation of the n→π* transition, promotion of the π→π* transition, and a net hypsochromic shift in the absorbance spectrum. **(B and C)** TDDFT reproduces this trend, with oscillator strength increasing in the n→π* transition of **(*Z*)-9** relative to **(*E*)-9**, while the oscillator strength for the π→π* transition increased for **(*E*)-9** relative to **(*Z*)-9**. **(D)** The predicted absorbance structure of RPE65-**(*E*)-9** shows an absorbance almost completely dominated by the π →π* transition. Models 1-4 use the functionals B3LYP, CAM-B3LYP, PBE0, and wB97x, respectively.

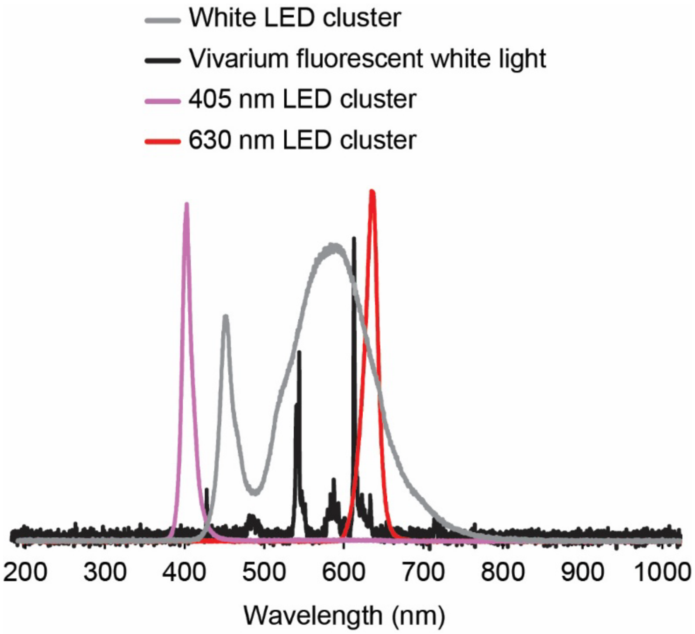

Figure S5: UV-Vis spectrum of light sources.

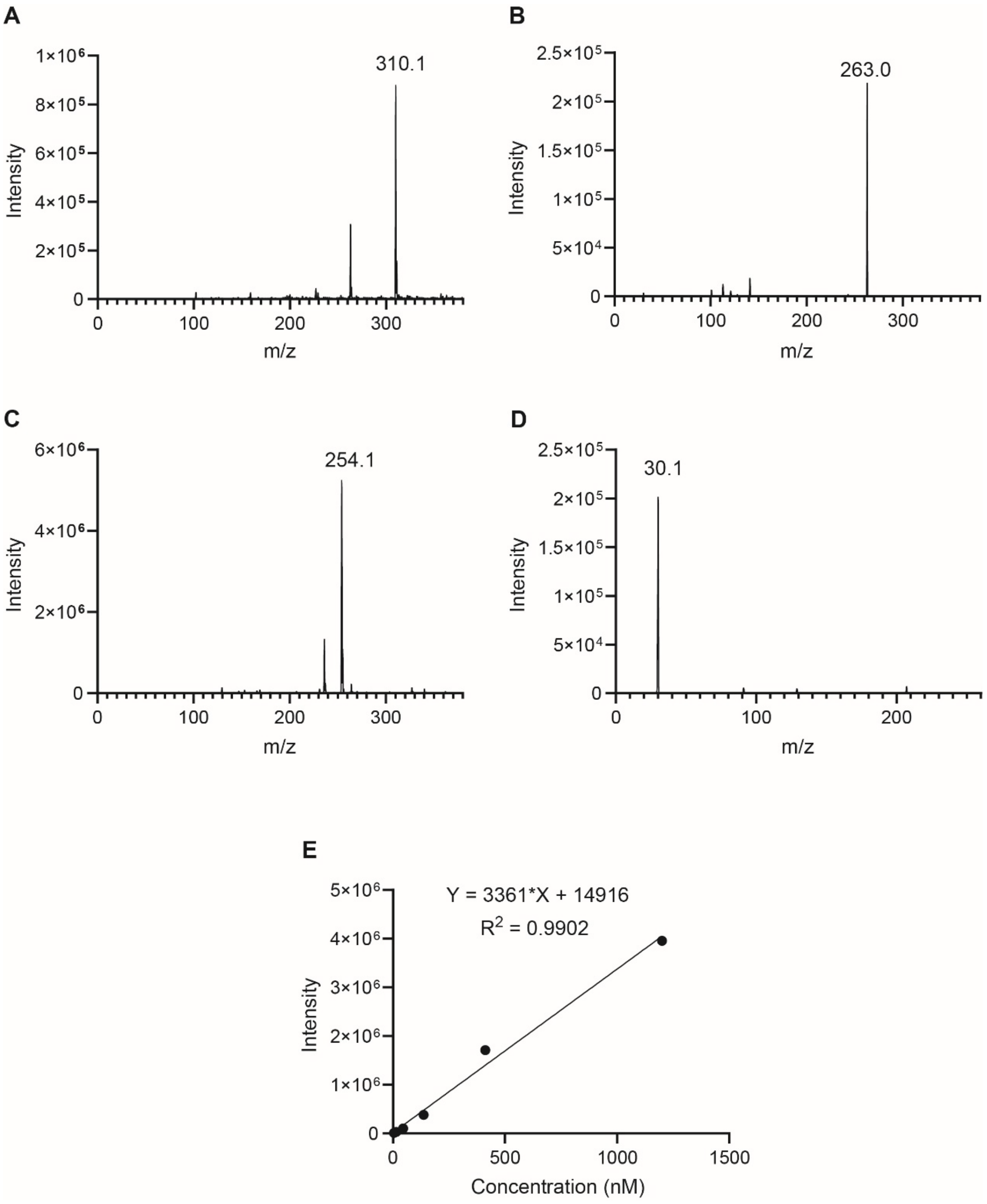

Figure S6: LC-MS/MS method for (*E*)-3 and (*E*)-9.

(**A and B**) Illustrative LC-MS/MS intensity response showing **(*E*)-9** parent and daughter ions. (**C and D**) Illustrative LC-MS/MS intensity response showing **(*E*)-3** parent and daughter ions. (**E**) Calibration curve of **(*E*)-9** based on the daughter ion. Results are shown as Mean ± SD, n = 3.

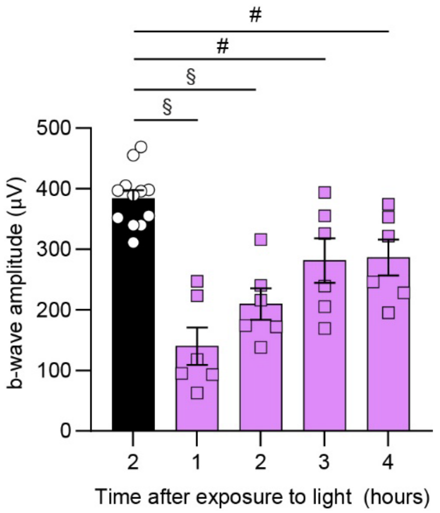

Figure S7: Effect of 10 min illumination with 405 nm LED light on dark adaptation.

Quantification of scotopic b- waves from 6-8 week-old BALBc/J mice photobleached by a 10 min exposure of white LED light (10,000 lux) (black bar) or violet LED light (3000 lux) (purple bars) after a period of dark adaptation (1-4 h). Scotopic ERG measurements were run using a flash intensity of 0.1 cd · s/m^2. Data represent mean ± SEM, each data point corresponds to one eye. The results of statistical t-test are reported as $^{ns}p > 0.05$, $^{\#}p < 0.01$, $^{§}p < 0.0001$.

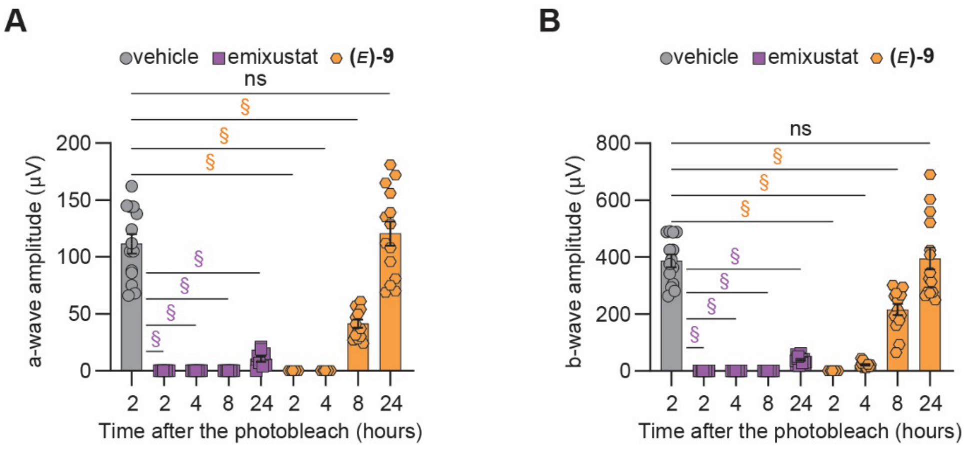

Figure S8: (*E*)-9 is active after oral administration and a short-acting RPE65 inhibitor.

Quantification of scotopic a- and b- waves, respectively. Dark-adapted, 6-8 weeks old mice were illuminated for 10 min (10,000 lux) and then received a single oral dose (5 mg/kg, 10% DMSO in soybean oil, 100 µL) in the dark. The course of dark adaptation was followed at regular intervals (2 - 24h) after drug administration by scotopic ERG measurements using a flash intensity of 0.1 cd · s/m^2. Data represent mean ± SEM, each data point corresponds to one eye. ANOVA analysis showed a statistically

significant difference between the groups ($p < 0.0001$). The results of Dunnett's multiple comparison test are reported as $^{ns}p > 0.05$, $^{\S}p < 0.0001$.

Detailed Synthesis Methods

General Synthetic Procedure for Knoevenagel Condensation

An oven dried 100 mL round bottom flask with a magnetic stir bar was charged with 0.52 mL acetonitrile (10.06 mmol, 1.7 equiv), 10 mL anhydrous THF under -78 °C. 10 mL 1M LDA in THF (6 mmol, 1.7 equiv) was added to react for 1 h with stirring. Then corresponding benzaldehyde (5.92 mmol, 1 equiv) in 10 mL THF was added to react under -78°C with stirring. After 6 hours, the reaction mixture was quenched with 30 mL 1M HCl and extracted by 3 × 100 mL ethyl acetate, dried over Na_2SO_4, and concentrated under reduced pressure. Crude products were purified by flash column chromatography using hexanes/ethyl acetate = 3/1 to give pure products **2a-2b**, **4a-4b** as yellow solids.

General Synthetic Procedure for Catalytic Hydrogenation

An oven dried 50 mL round bottom flask with a magnetic stir bar was charged with 200 mg β-hydroxynitrile (1.36 mmol, 1 equiv), palladium on activated carbon (40 mg, 20 wt%). 15 mL methanol was added as solvent. The flask was placed into a high-pressure reaction vessel and degased under vacuum followed by refilling with hydrogen gas to 200 psi. The mixture was left to react under room temperature with vigorous stirring. After 24 hours, the high-pressure reaction vessel was carefully vented, the crude reaction mixture was filtered by gravity, and the solvent was removed under reduced pressure. The crude product was purified by flash column chromatography using hexane/ethyl acetate = 1.5/1 to give pure products **5a-5b** as white to pale yellow solids.

General Synthetic Procedure for Nitrile Reduction

A 100 mL high pressure reaction flask was sealed with a magnetic stir bar was charged with nitrile (1 equiv), 10 mL anhydrous THF, and 1M $LiAlH_4$ in THF (5 equiv). The reaction tube was sealed and heated to 50°C (cooled to 0°C for **2a**) with stirring. After 24 hours (1 hour for **2a**), the reaction tube was cooled to room temperature, the reaction mixture was quenched by 30 mL methanol and concentrated under reduced pressure. The crude product was purified by flash column chromatography using ethyl acetate/methanol/ammonium hydroxide = 9/1/0.4 to give pure products **3a-4b**, **6a-6b** as pale yellow oil.

Synthetic Procedure for 2,6-Difluoronitrosobenzene

Following a published work[12], a 100 mL round bottom flask with a magnetic stir bar was charged with oxone (2.04 equiv), and water (30 mL) to have a completely transparent solution, then solution of 2,6-difluoroaniline (1 equiv) in DCM (15 mL) was slowly added to the aqueous solution via glass Pasteur pipet. The reaction was run under room temperature with stirring. After 24 hours, the reaction mixture was extracted by 3 × 100 mL DCM. The combined organic layer was dried over Na_2SO_4, and

concentrated under reduced pressure to give crude product as tan solid. The crude product was used without any further purification.

General Synthetic Procedure for Azobenzene Derivatives

Following a published work[13], a 100 mL round bottom flask with a magnetic stir bar was charged with aromatic amine **6a-6b** (1 equiv), corresponding nitrosobenzene (1.1 equiv), and 15 mL AcOH. The reaction was run under room temperature in dark with stirring. After 24 hours, the reaction mixture was quenched by 5M NaOH (50 mL) and extracted by 3 × 100 mL EtOAc. The combined organic layer was dried over Na_2SO_4, and concentrated under reduced pressure. The crude product was purified by flash column chromatography using ethyl acetate/methanol/ammonium hydroxide = 14/1/0.4 to give pure products **(*E*)-7**, **(*E*)-8**, and **(*E*)-9** as orange solid.

Synthetic Procedure for (*E*)-3-styrylbenzaldehyde (1a)

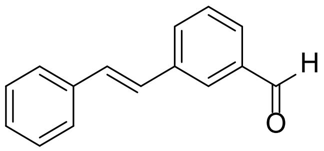

1a

Following a published work[14], a 2-necked 25 mL round bottom flask with a magnetic stir bar was charged with 3-bromobenzaldehyde (1 equiv), palladium (II) acetate (1 mol%), triethanolamine (1.84 equiv), and styrene (1.02 equiv, filter through basic alumina before use to remove stabilizer). The flask was purged under vacuum and flushed with argon for 5 times, then was heated to 120°C overnight under argon with stirring. After cooling to room temperature, the mixture was diluted with water (30 mL), and extracted with 5 × 10 mL DCM. The combined organic layer was dried over Na_2SO_4, and concentrated under reduced pressure. The crude product was purified by flash column chromatography using hexane /ethyl acetate = 2.5/1 to give pure products **1a** as yellow solid. **1a** was purified by flash column chromatography using hexanes/ethyl acetate = 2.5/1 as yellow solid, yield 50%. **Figure S7:** ^1H NMR (500 MHz, $CDCl_3$) δ 10.04 (s, 1H), 8.01 (s, 1H), 7.75 (d, *J* = 7.5 Hz, 2H), 7.53-7.50 (m, 3H), 7.39 (dd, *J* = 7.5 Hz, 2H), 7.28 (dd, *J* = 7.5 Hz, 1H), 7.22 (d, *J* = 16.4 Hz, 1H), 7.20 (d, *J* = 16.0 Hz, 1H). **Figure S8:** $^{13}C(^1H)$ NMR (126 MHz, $CDCl_3$) δ 192.6, 138.6, 137.0, 136.9, 132.6, 130.8, 129.6, 129.1, 129.0, 128.4, 127.4, 127.3, 126.9. HRMS (ESI): (m/z) calculated for $C_{15}H_{12}O$ $[M]^+$ 208.0888; found 208.0892.

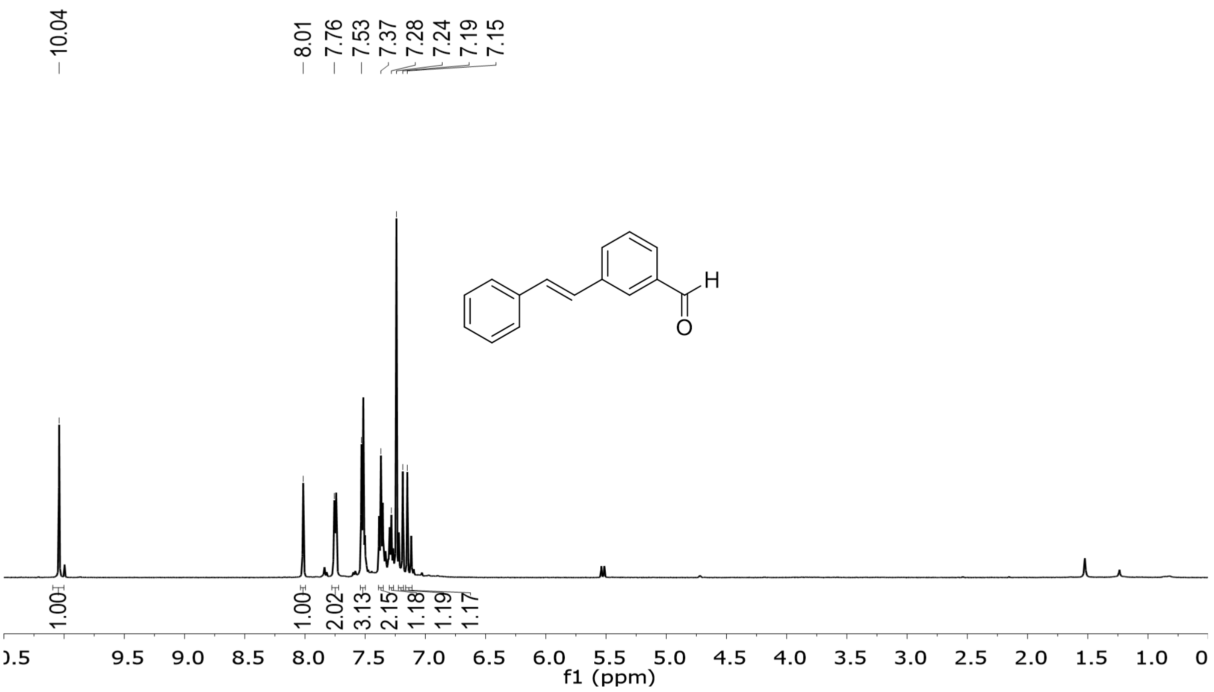

Figure S9: ^{1}H NMR spectrum of (*E*)-3-styrylbenzaldehyde **(1a)** ($CDCl_3$, 500 MHz).

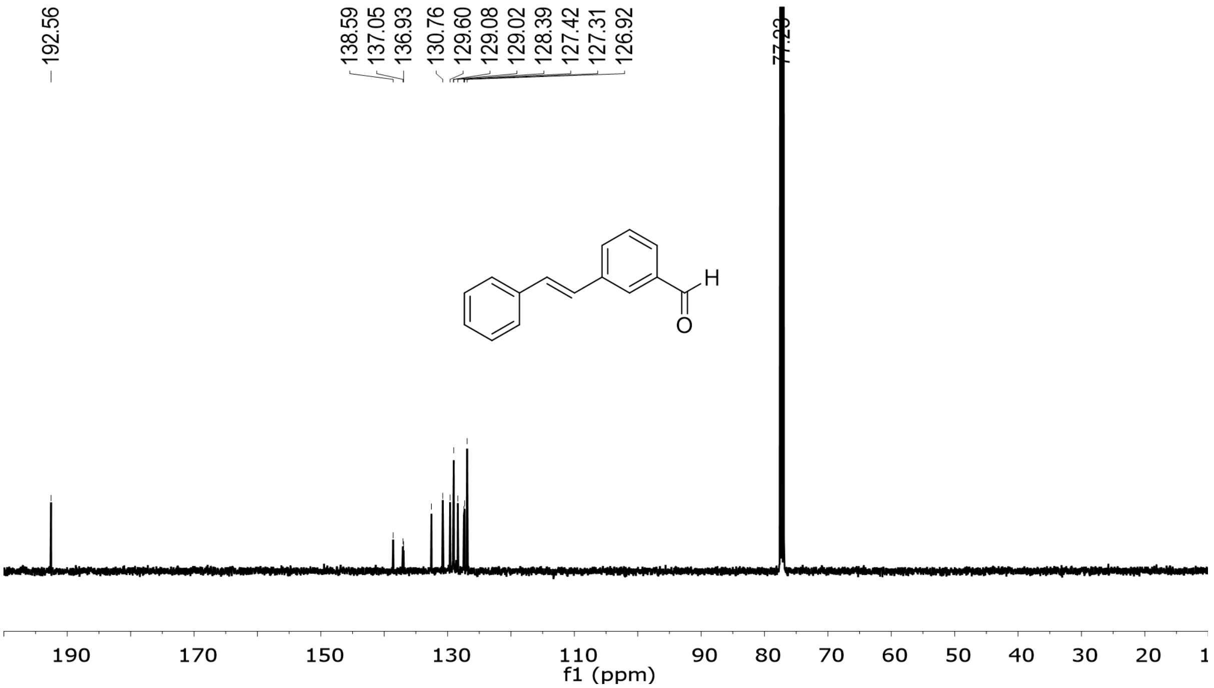

Figure S10: ^{13}C NMR spectrum of (*E*)-3-styrylbenzaldehyde **(1a)** ($CDCl_3$, 126 MHz).

Synthetic Procedure for (*Z*)-(2-bromovinyl)benzene

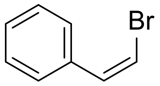

Following a published work[15], a 100 mL round bottom flask with a magnetic stir bar was charged with (*E*)-cinnamic acid (1 equiv), $CHCl_3$ (25 mL) and cooled to 0 °C. Bromine (1.08 equiv) was then added dropwise through an addition funnel. The reaction mixture slowly warmed to room temperature with stirring. After 16 hours, the reaction mixture was filtered and wash with cold 3 × 30 mL $CHCl_3$ to give 2,3-dibrom-3-phenylpropanoic acid as light-orange solid. Crude 2,3-dibrom-3-phenylpropanoic acid (1 equiv) was dissolved in DMF (30 mL) and cooled to 0 °C in a 100 mL round bottom flask with a magnetic stir. Triethylamine (7.17 equiv) was added dropwise to the solution through an addition funnel. The reaction was run under 0°C with stirring. After for 3 hours the reaction mixture was diluted with water (50 mL), and extracted with 3 × 100 mL EtOAc. The combined organic layer was washed with 2 × 100 mL saturated K_2CO_3 in water, dried over Na_2SO_4, and concentrated under reduced pressure. The crude product was purified by flash column chromatography using hexane /ethyl acetate = 20/1 to give pure product as colorless oil.

Synthetic Procedure for (*Z*)-3-styrylbenzaldehyde

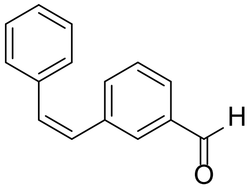

1b

Following a published work[15], a 2-necked 25 mL round bottom flask with a magnetic stir bar was charged with (3-formylphenyl)boronic acid (1.1 equiv), tetrakis(triphenylphosphine) palladium (0) (2 mol%), tri(o-tolyl)phosphine (4 mol%), sodium carbonate (3.1 equiv). This flask was purged under vacuum and flushed with argon for 3 times. In a pear-shaped flask, ethanol (6 mL), water (1 mL), and (*Z*)-(2-bromovinyl)benzene (1 equiv) was bubbled with argon. The reaction mixture from in the pear-shaped flask were then cannulated into the round bottom flask then was then heated at 80°C overnight under argon with stirring. After cooling to room temperature, the mixture was diluted in ethyl acetate (5 mL), filtered through celite, and concentrated under reduced pressure. The crude material was purified by flash column chromatography using hexane /ethyl acetate = 5/1 to give pure products **1b** as yellow oil yield 37%. ^1H NMR (500 MHz, $CDCl_3$) δ 9.88 (s, 1H), 7.70 (d, J = 12.5 Hz, 2H), 7.47 (d, J = 8.0 Hz, 1H), 7.35 (dd, J = 7.5 Hz, 1H), 7.20 (m, 5H), 6.70 (d, J = 12.0 Hz, 1H), 6.61 (d, J = 12.0 Hz, 1H). $^{13}C(^1H)$ NMR

(126 MHz, $CDCl_3$) δ 192.5, 138.5, 136.8, 136.7, 135.0, 132.2, 130.9, 129.1, 129.0, 128.9, 128.6, 128.2, 127.8. HRMS (ESI): (m/z) calculated for $C_{15}H_{12}NaO$ $[M+Na]^+$ 232.0864; found 232.0814.

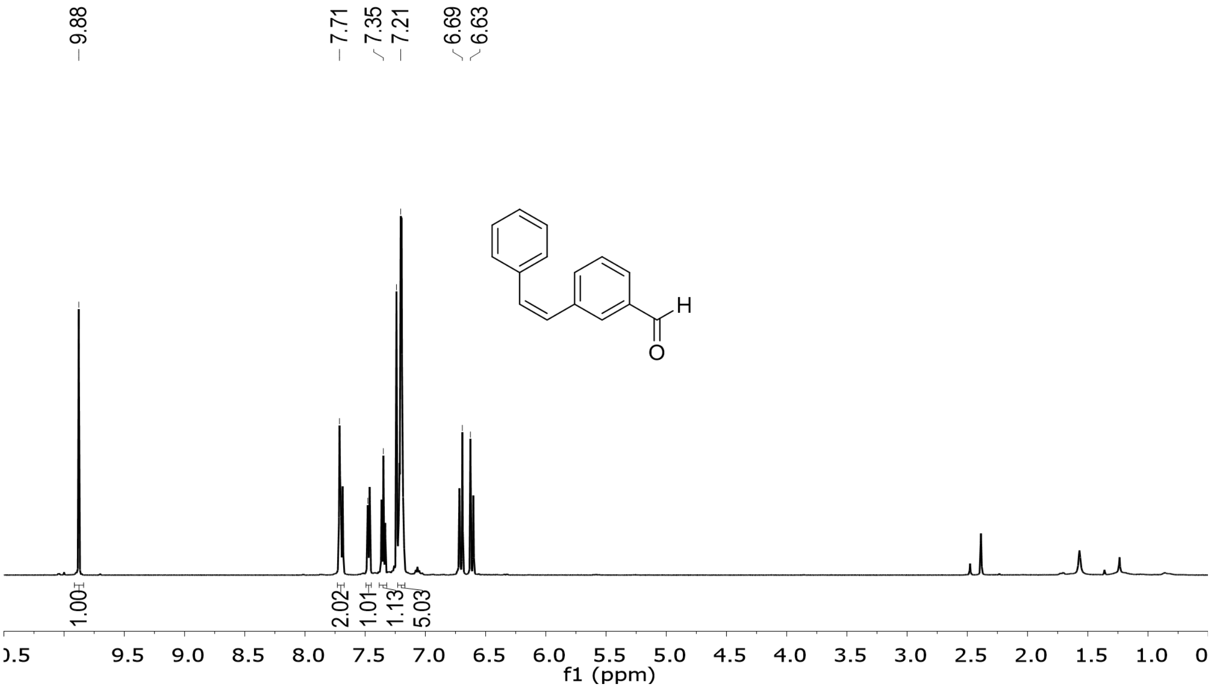

Figure S11: ^{1}H NMR spectrum of (*Z*)-3-styrylbenzaldehyde **(1b)** ($CDCl_3$, 500 MHz).

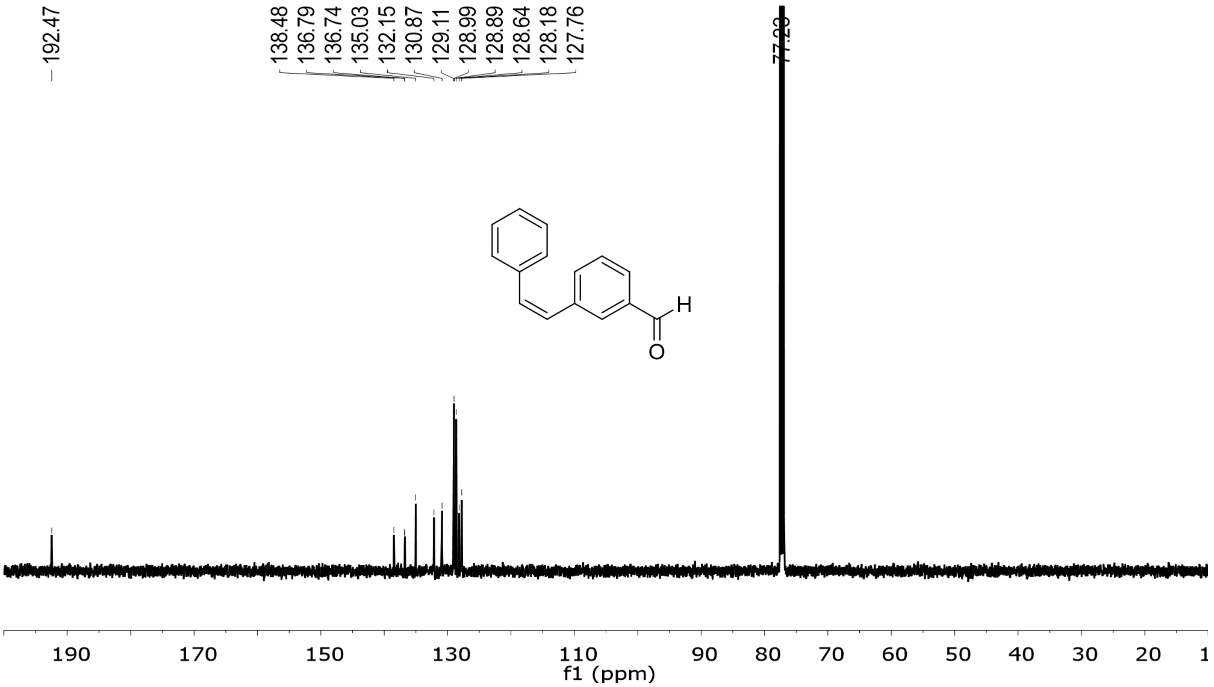

Figure S12: ^{13}C NMR spectrum of (*Z*)-3-styrylbenzaldehyde **(1b)** ($CDCl_3$, 126 MHz).

Synthetic Procedure for (*E*)-3-hydroxy-3-(3-styrylphenyl)propanenitrile (2a)

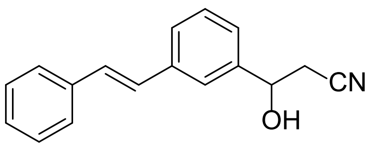

2a

2a was synthesized following general synthetic procedure (purified by flash column chromatography using hexanes/ethyl acetate = 2.5/1) as colorless oil, yield 63%. ^{1}H NMR (500 MHz, $CDCl_3$) δ 7.58 (s, 1H), 7.54 (m, 3H), 7.40-7.34 (m, 3H), 7.28-7.26 (m, 2H), 7.13 (d, *J* = 16.5 Hz, 1H), 7.09 (d, *J* = 16.5 Hz, 1H), 5.07 (dd, *J* = 6.5 Hz, 1H), 2.81-2.79 (m, 2H), 2.28 (s, 1H). ^{13}C(^{1}H) NMR (126 MHz, $CDCl_3$) δ 141.7, 138.3, 137.2, 129.9, 129.6, 129.0, 128.2, 128.1, 127.2, 126.8, 124.8, 123.7, 117.4, 70.5, 28.2. HRMS (ESI): (m/z) calculated for $C_{17}H_{15}NO$ $[M]^+$ 249.1154; found 249.1157.

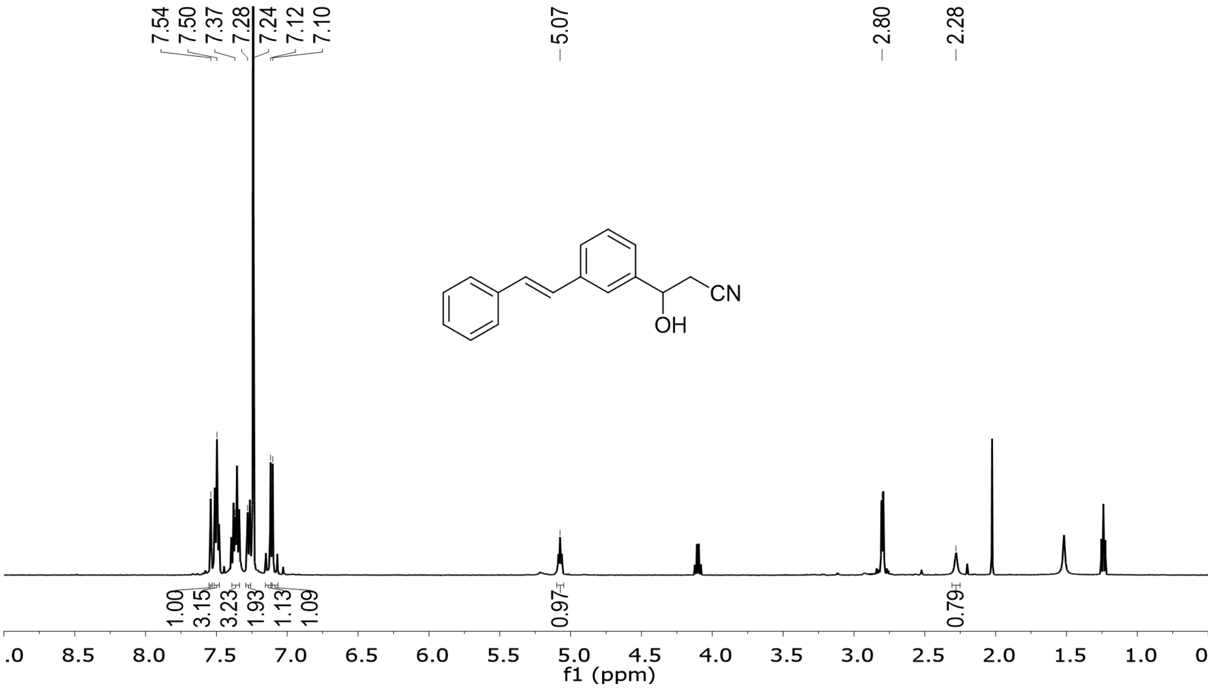

Figure S13: ^{1}H NMR spectrum of (*E*)-3-hydroxy-3-(3-styrylphenyl)propanenitrile **(2a)** ($CDCl_3$, 500 MHz).

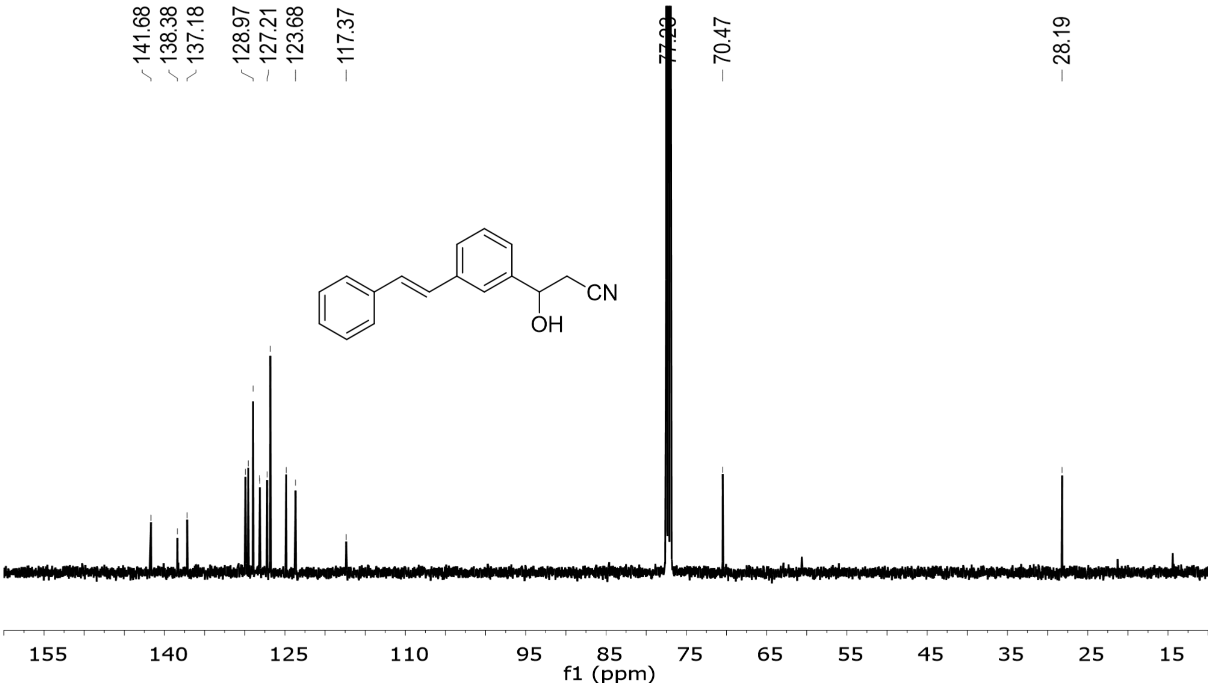

Figure S14: ^{13}C NMR spectrum of (*E*)-3-hydroxy-3-(3-styrylphenyl)propanenitrile **(2a)** ($CDCl_3$, 126 MHz).

Synthetic Procedure for (*Z*)-3-hydroxy-3-(3-styrylphenyl)propanenitrile (2b)

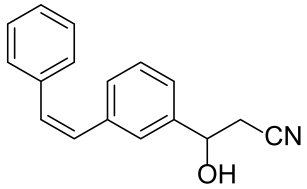

2b

2b was synthesized following general synthetic procedure (purified by flash column chromatography using hexanes/ethyl acetate = 2.5/1) as yellow oil, yield 45%. ^{1}H NMR (500 MHz, $CDCl_3$) δ 7.26-7.19 (m, 8H), 6.65 (d, *J* = 12.0 Hz, 1H), 6.57 (d, *J* = 12.0 Hz, 1H), 4.91-4.88 (m, 1H), 2.66-2.57 (m, 2H), 2.11 (d, *J* = 3.5 Hz, 1H). $^{13}C(^{1}H)$ NMR (126 MHz, $CDCl_3$) δ 141.0, 138.2, 137.2, 131.4, 129.8, 129.7, 129.2, 129.1, 128.5, 127.6, 126.3, 124.5, 117.3, 70.3, 27.9. HRMS (ESI): (m/z) calculated for $C_{17}H_{15}NNaO$ $[M+Na]^+$ 273.1130; found 273.1180.

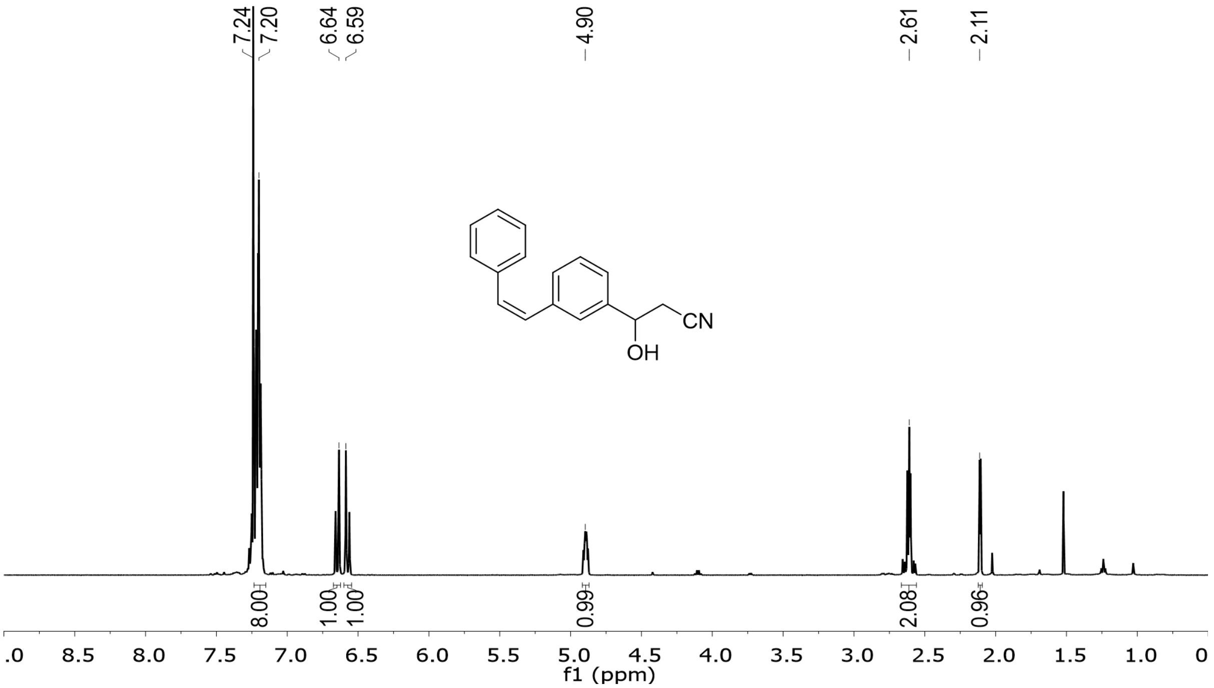

Figure S15: ^{1}H NMR spectrum of (*Z*)-3-hydroxy-3-(3-styrylphenyl)propanenitrile **(2b)** ($CDCl_3$, 500 MHz).

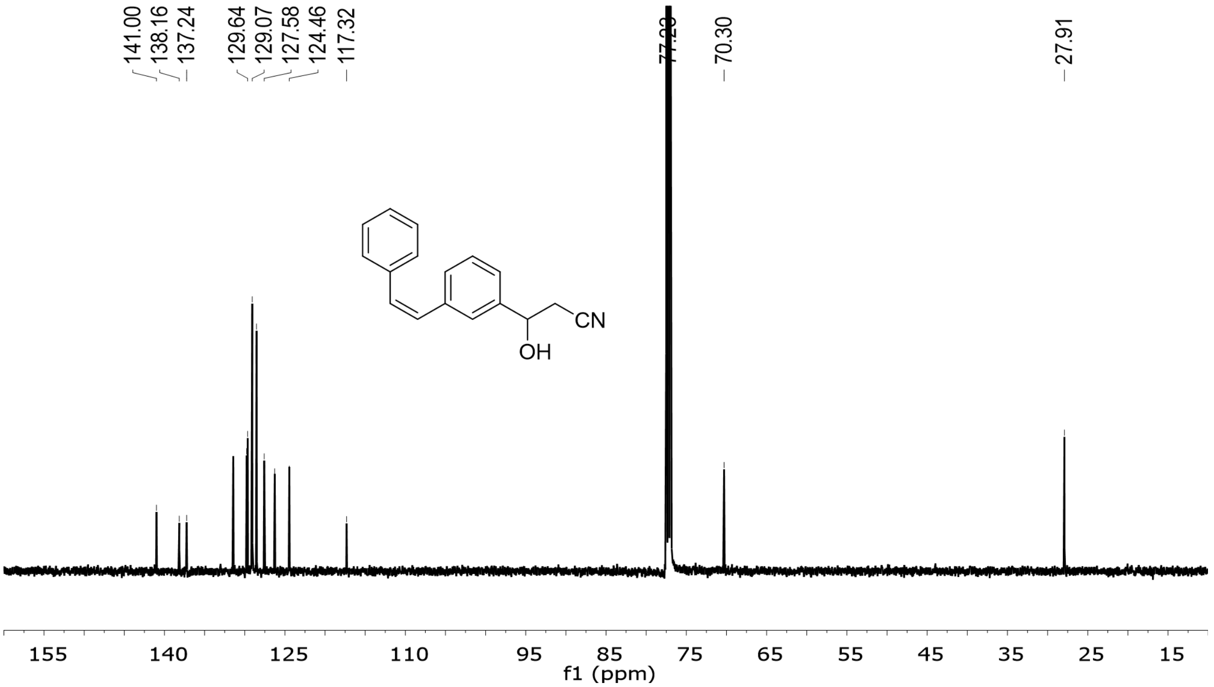

Figure S16: ^{13}C NMR spectrum of (*Z*)-3-hydroxy-3-(3-styrylphenyl)propanenitrile **(2b)** ($CDCl_3$, 126 MHz).

Synthetic Procedure for (*E*)-3-amino-1-(3-styrylphenyl)propan-1-ol (3a)

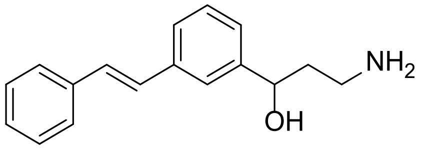

3a

3a was synthesized following general synthetic procedure (purified by flash column chromatography using ethyl acetate/methanol/ammonium hydroxide = 9/1/0.4 as colorless oil, yield 31%. ^{1}H NMR (500 MHz, $CDCl_3$) δ 7.56 (s, 1H), 7.49 (d, *J* = 7.5 Hz, 2H), 7.39-7.30 (m, 4H), 7.25 (s, 1H), 7.11 (s, 2H), 5.00 (dd, *J* = 8.5 Hz, 3.0 Hz, 1H), 3.15-3.10 (m, 1H), 3.01-2.96 (m, 1H), 1.92-1.87 (m, 1H), 1.80-1.73 (m, 1H). ^{13}C(^{1}H) NMR (126 MHz, $CDCl_3$) δ 145.8, 137.6, 137.5, 129.0, 128.9, 128.9, 128.8, 127.8, 126.7, 125.5, 125.3, 123.9, 75.9, 40.9, 39.8. HRMS (ESI): (m/z) calculated for $C_{17}H_{20}NO$ [M+H]$^+$ 254.1539; found 254.1540.

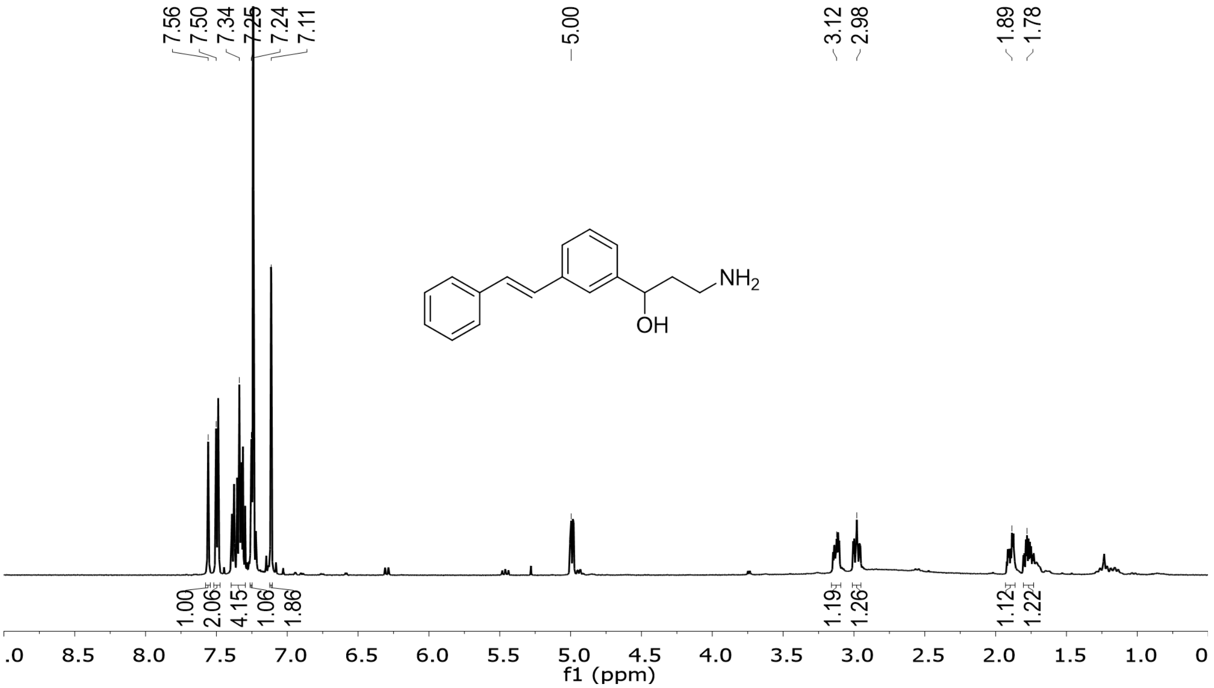

Figure S17: ^{1}H NMR spectrum of (*E*)-3-amino-1-(3-styrylphenyl)propan-1-ol **(3a)** ($CDCl_3$, 500 MHz).

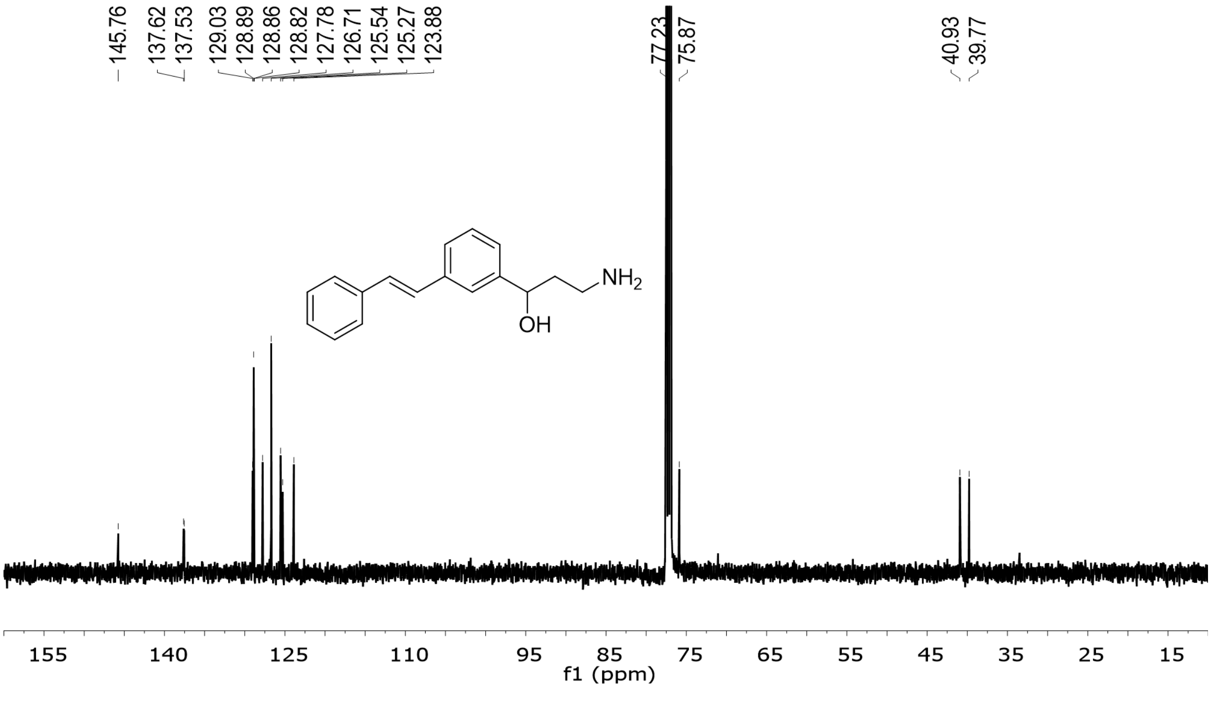

Figure S18: ^{13}C NMR spectrum of (*E*)-3-amino-1-(3-styrylphenyl)propan-1-ol **(3a)** ($CDCl_3$, 126 MHz).

Synthetic Procedure for (*Z*)-3-amino-1-(3-styrylphenyl)propan-1-ol (3b)

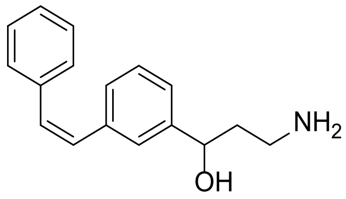

3b

3b was synthesized following general synthetic procedure (purified by flash column chromatography using ethyl acetate/methanol/ammonium hydroxide = 9/1/0.4) as pale yellow oil, yield 44%. ^{1}H NMR (500 MHz, $CDCl_3$) δ 7.23-7.11 (m, 9H), 6.60 (d, *J* = 12 Hz, 1H), 6.57 (d, *J* = 12 Hz, 1H), 4.84 (dd, *J* = 8.3 Hz, 3.0 Hz, 1H), 3.01-2.97 (m, 1H), 2.90-2.85 (m, 1H), 1.80-1.74 (m, 1H), 1.66-1.59 (m, 1H). ^{13}C(^{1}H) NMR (126 MHz, $CDCl_3$) δ 145.2, 137.6, 137.3, 130.7, 130.4, 129.1, 128.4, 128.4, 127.7, 127.2, 126.6, 124.7, 75.6, 40.6, 39.5. HRMS (ESI): (m/z) calculated for $C_{17}H_{20}NO$ $[M+H]^+$ 254.1539; found 254.1540.

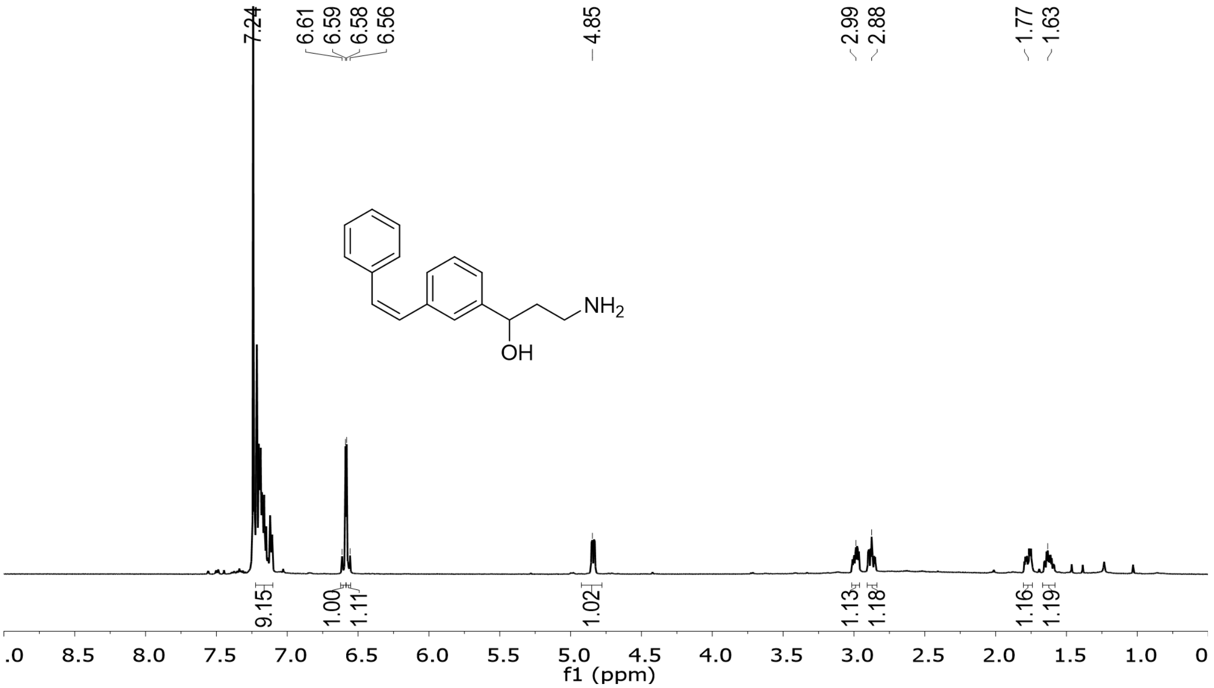

Figure S19: ^{1}H NMR spectrum of (*Z*)-3-amino-1-(3-styrylphenyl)propan-1-ol **(3b)** ($CDCl_3$, 500 MHz).

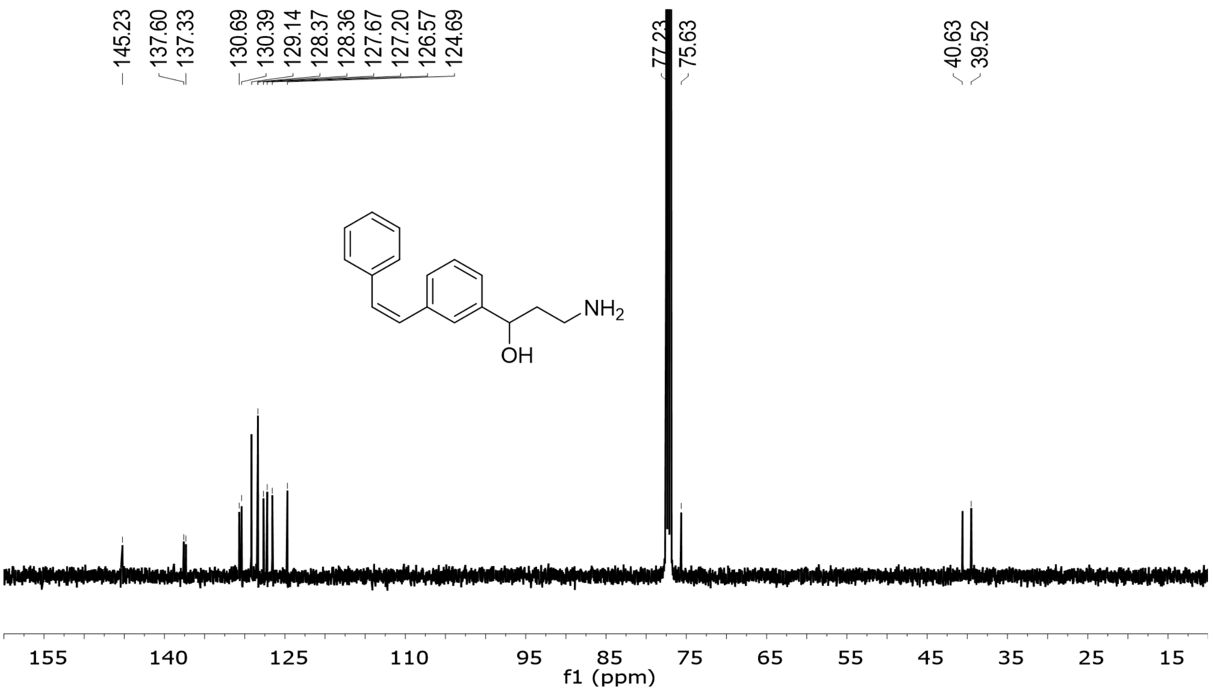

Figure S20: ^{13}C NMR spectrum of (*Z*)-3-amino-1-(3-styrylphenyl)propan-1-ol **(3b)** ($CDCl_3$, 125 MHz).

Synthetic Procedure for 3-(3-Nitrophenyl)-3-hydroxypropanenitrile (4a)

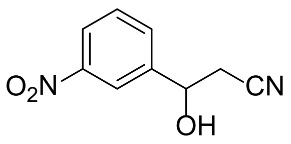

4a

4a was synthesized following general synthetic procedure (purified by flash column chromatography using hexanes/ethyl acetate = 3/1) as yellow solid, yield 41%. ^{1}H NMR (500 MHz, $CDCl_3$) δ 8.29 (dd, *J* = 2.0 Hz, 1H), 8.23-8.20 (m, 1H), 7.78 (d, *J* = 7.5 Hz, 1H), 7.60 (dd, *J* = 8.0 Hz, 1H), 5.19 (dd, *J* = 6.0 Hz, 1H), 2.82 (s, 1H), 2.81 (s, 1H), 2.52 (s, 1H). ^{13}C(^{1}H) NMR (126 MHz, $CDCl_3$) δ 148.8, 143.1, 131.9, 130.3, 124.0, 120.9, 116.6, 69.4, 28.3. HRMS (ESI): *m/z* calculated for $C_9H_8N_2O_3$ $[M]^+$ 192.0535; found 192.0533.

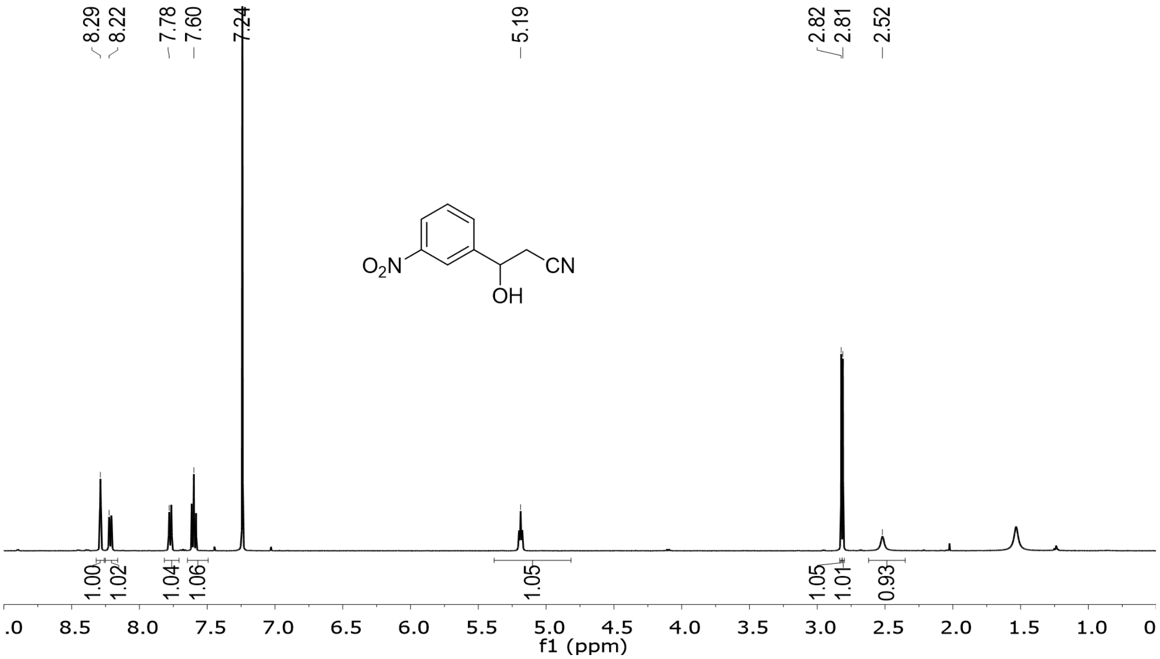

Figure S21: ^{1}H NMR spectrum of 3-(3-Nitrophenyl)-3-hydroxypropanenitrile **(4a)** ($CDCl_3$, 500 MHz).

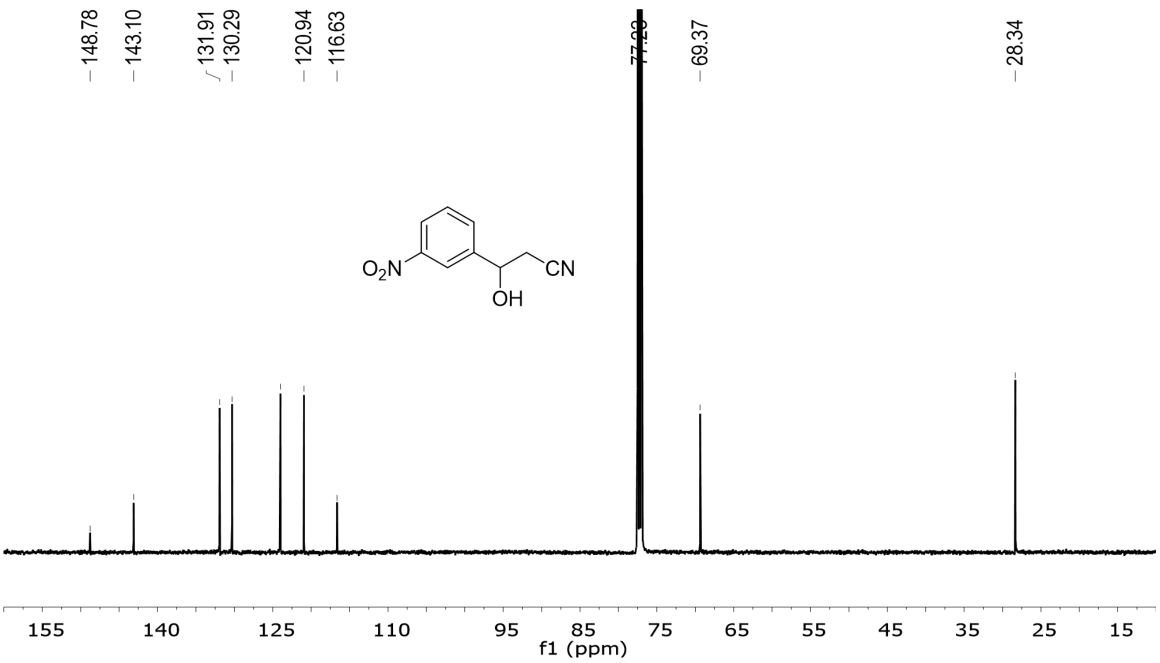

Figure S22: ^{13}C NMR spectrum of 3-(3-Nitrophenyl)-3-hydroxypropanenitrile **(4a)** ($CDCl_3$, 126 MHz).

Synthetic Procedure for 3-(4-Fluoro-3-nitrophenyl)-3-hydroxypropanenitrile (4b)

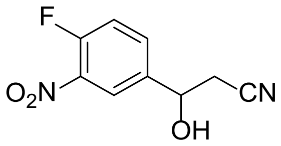

4b

4b was synthesized following general synthetic procedure (purified by flash column chromatography using hexanes/ethyl acetate = 2.5/1) as yellow solid, yield 43%. ^{1}H NMR (500 MHz, $CDCl_3$) δ 8.12 (dd, J = 6.8 Hz, 2.0 Hz, 1H), 7.72-7.70 (m, 1H), 7.33 (dd, J = 10.2 Hz, 9.0 Hz, 1H), 5.14 (dd, J = 6.0 Hz, 1H), 2.97(s, 1H), 2.83-2.75 (m, 3H). ^{13}C(^{1}H) NMR (126 MHz, $CDCl_3$) δ 155.5 (d, $J_{C\text{-}F}$ = 265.86 Hz), 138.2 (d, $J_{C\text{-}F}$ = 3.78 Hz), 137.6 (d, $J_{C\text{-}F}$ = 7.56 Hz), 133.0 (d, $J_{C\text{-}F}$ = 8.82 Hz), 123.7 (d, $J_{C\text{-}F}$ = 2.52 Hz), 119.32 (d, $J_{C\text{-}F}$ = 21.42 Hz), 116.7, 68.5, 28.3. ^{19}F NMR (471 MHz, $CDCl_3$) δ -117.06. HRMS (ESI): *m/z* calculated for $C_9H_7FN_2O_3$ $[M]^+$ 210.0441; found 210.0434.

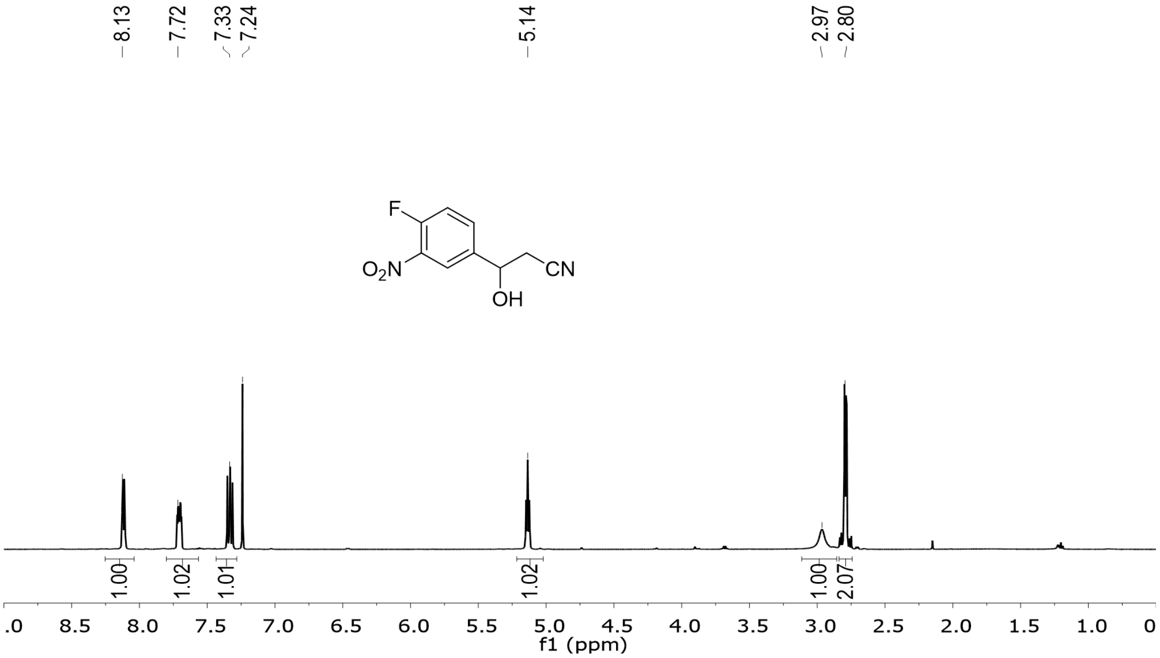

Figure S23: ^{1}H NMR spectrum of 3-(4-Fluoro-3-nitrophenyl)-3-hydroxypropanenitrile **(4b)** ($CDCl_3$, 500 MHz).

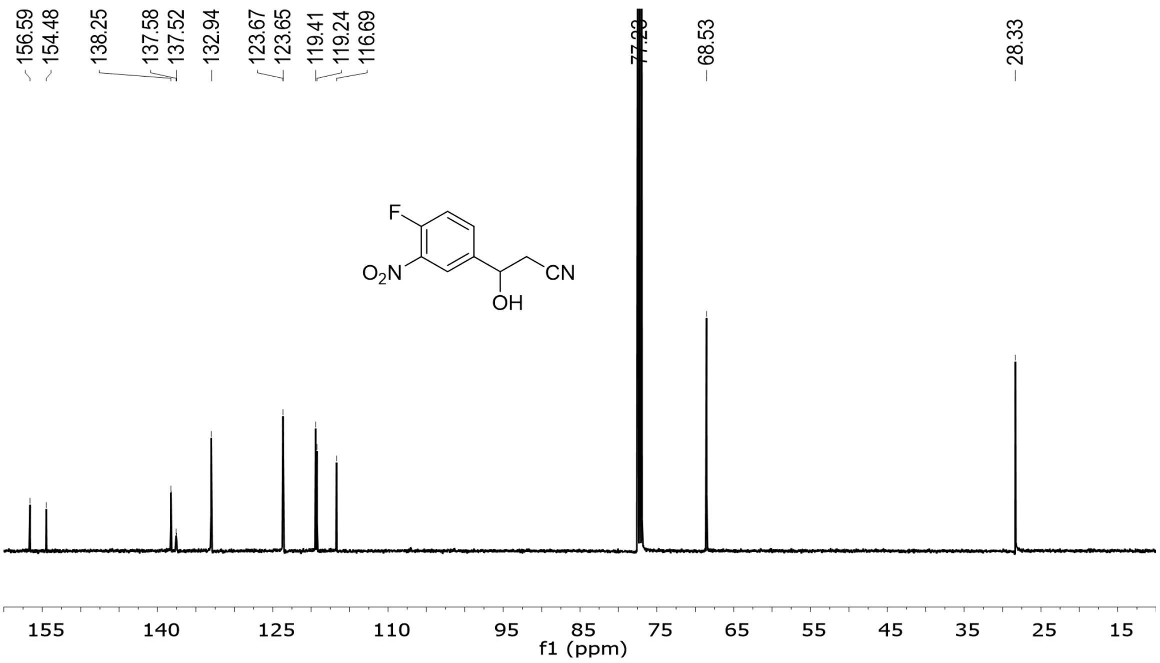

Figure S24: ^{13}C NMR spectrum of 3-(4-Fluoro-3-nitrophenyl)-3-hydroxypropanenitrile **(4b)** ($CDCl_3$, 126 MHz).

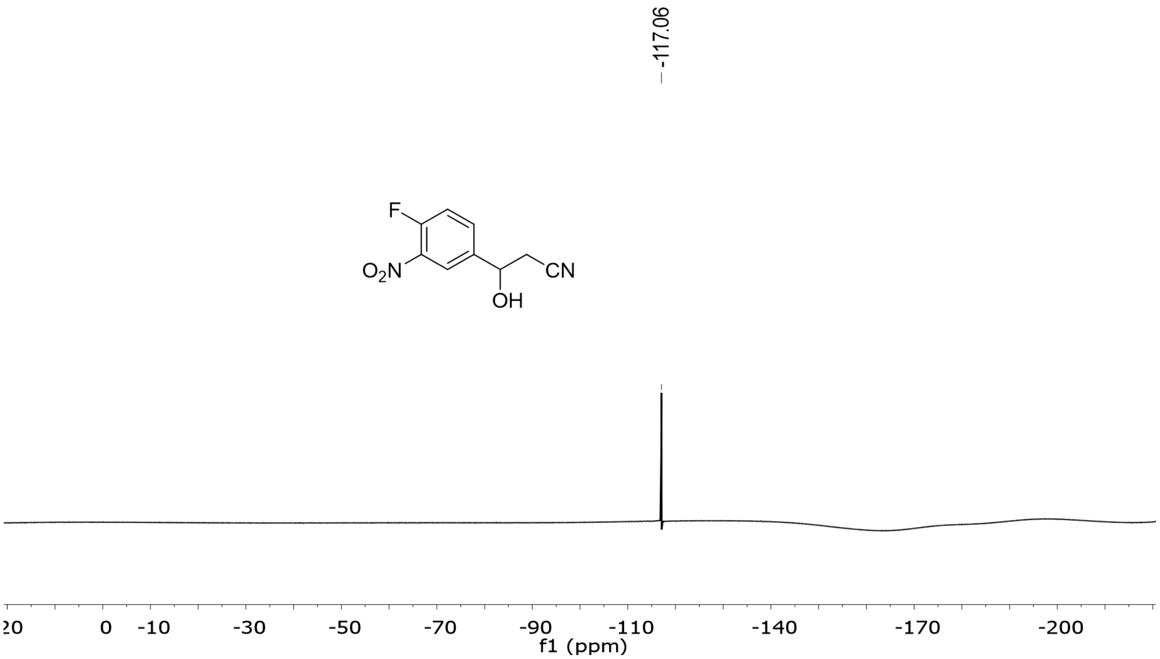

Figure S25: ^{19}F NMR spectrum of 3-(4-Fluoro-3-nitrophenyl)-3-hydroxypropanenitrile **(4b)** ($CDCl_3$, 471 MHz).

Synthetic Procedure for 3-(3-Aminophenyl)-3-hydroxypropanenitrile (5a)

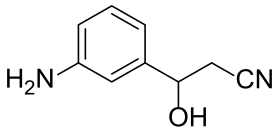

5a

5a was synthesized following general synthetic procedure (purified by flash column chromatography using hexanes/ethyl acetate = 1.5/1) as white solid, yield 70%. ^{1}H NMR (500 MHz, CD_3OD) δ 7.10 (dd, J = 7.5 Hz, 1H), 6.80 (s, 1H), 6.74 (d, J = 7.5 Hz, 1H), 6.67 (d, J = 7.5 Hz, 1H), 4.85 (dd, J = 6.8 Hz, 5.5 Hz), 2.85-2.74 (m, 2H). $^{13}C(^1H)$ NMR (126 MHz, CD_3OD) δ 149.2, 144.8, 130.4, 119.2, 116.6, 116.4, 113.8, 70.9, 28.5. HRMS (ESI): m/z calculated for $C_9H_{10}N_2O$ $[M]^+$ 162.0793; found 163.0865 $[M+H]^+$.

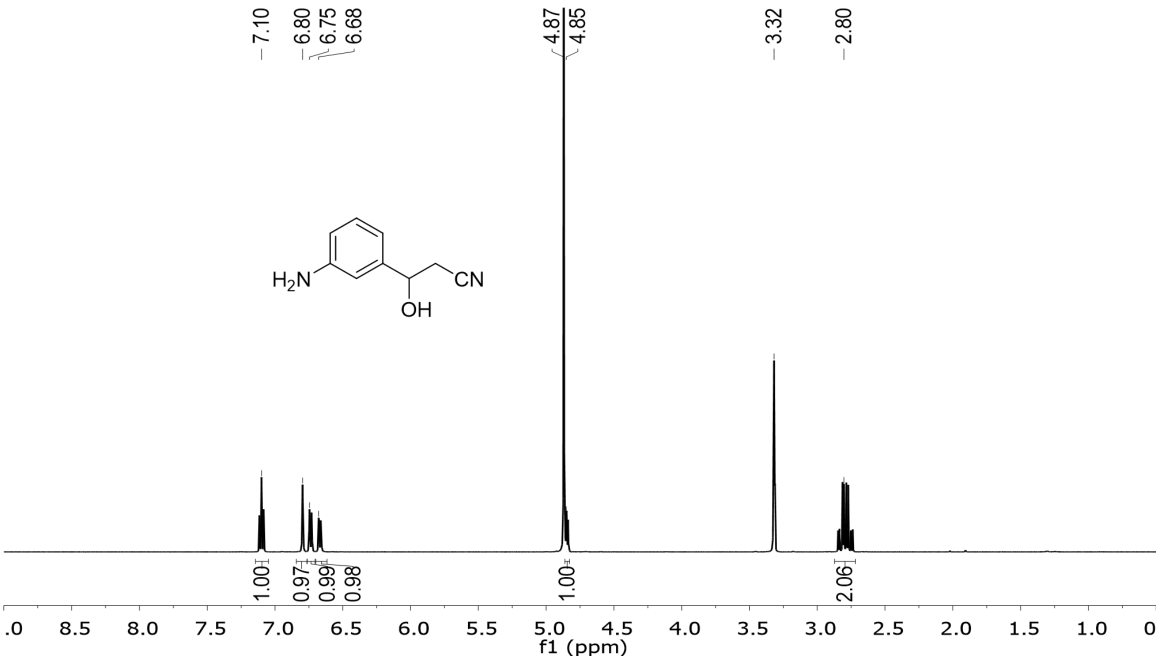

Figure S26: ^{1}H NMR spectrum of 3-(3-Aminophenyl)-3-hydroxypropanenitrile **(5a)** (CD_3OD, 500 MHz).

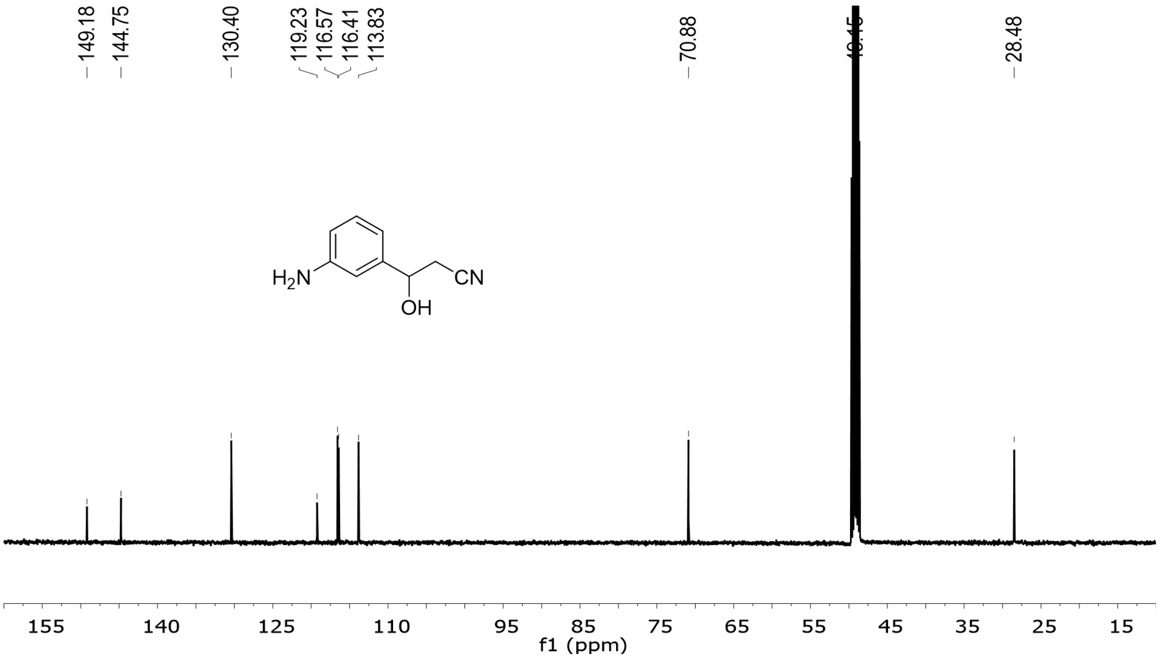

Figure S27: ^{13}C NMR spectrum of 3-(3-Aminophenyl)-3-hydroxypropanenitrile **(5a)** (CD_3OD, 126 MHz).

Synthetic Procedure for 3-(3-Amino-4-fluorophenyl)-3-hydroxypropanenitrile (5b)

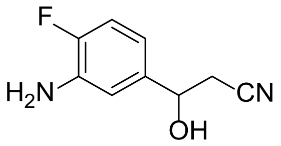

5b

5b was synthesized following general synthetic procedure (purified by flash column chromatography using hexanes/ethyl acetate = 1.5/1) as pale yellow solid, yield 65%. **Figure S26:** ^{1}H NMR (500 MHz, CD_3OD) δ 6.96-6.92 (m, 2H), 6.71-6.68 (m, 1H), 4.86 (dd, *J* = 6.0 Hz, 1H), 2.84-2.74 (m, 2H). **Figure S27:** ^{13}C(^{1}H) NMR (126 MHz, CD_3OD) δ 152.9 (d, $J_{C\text{-}F}$ = 112.14 Hz), 140.2 (d, $J_{C\text{-}F}$ = 13.86 Hz), 119.1, 116.3 (d, $J_{C\text{-}F}$ = 7.56 Hz), 115.9 (d, $J_{C\text{-}F}$ = 18.90 Hz), 115.4 (d, $J_{C\text{-}F}$ = 3.78 Hz), 70.3, 28.6. **Figure S28:** ^{19}F NMR (471 MHz, CD_3OD) δ -138.42. HRMS (ESI): *m/z* calculated for $C_9H_9FN_2O$ [M]$^+$ 180.0699; found 180.0699.

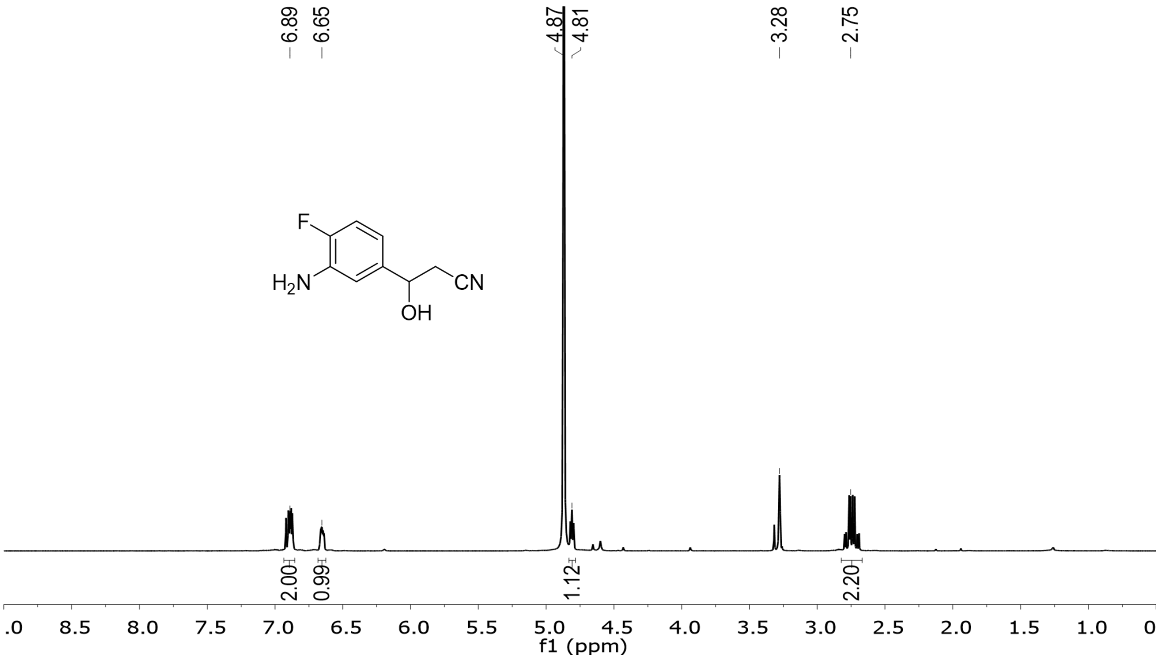

Figure S28: ^{1}H NMR spectrum of 3-(3-Amino-4-fluorophenyl)-3-hydroxypropanenitrile **(5b)** (CD_3OD, 500 MHz).

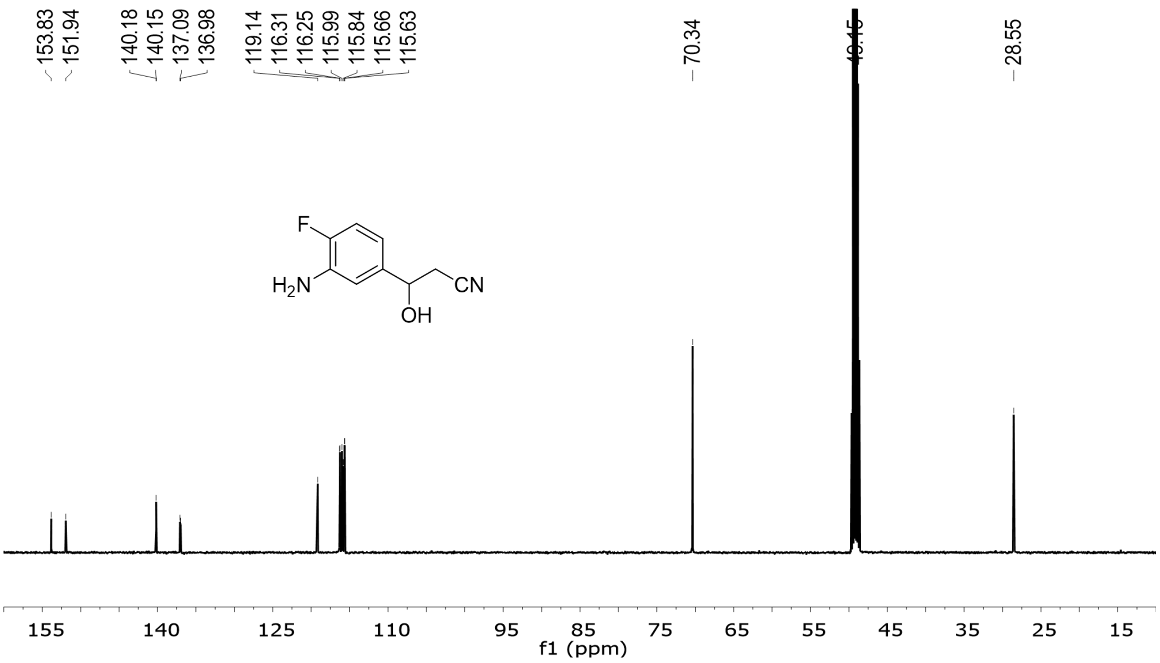

Figure S29: ^{13}C NMR spectrum of 3-(3-Amino-4-fluorophenyl)-3-hydroxypropanenitrile **(5b)** (CD_3OD, 126 MHz).

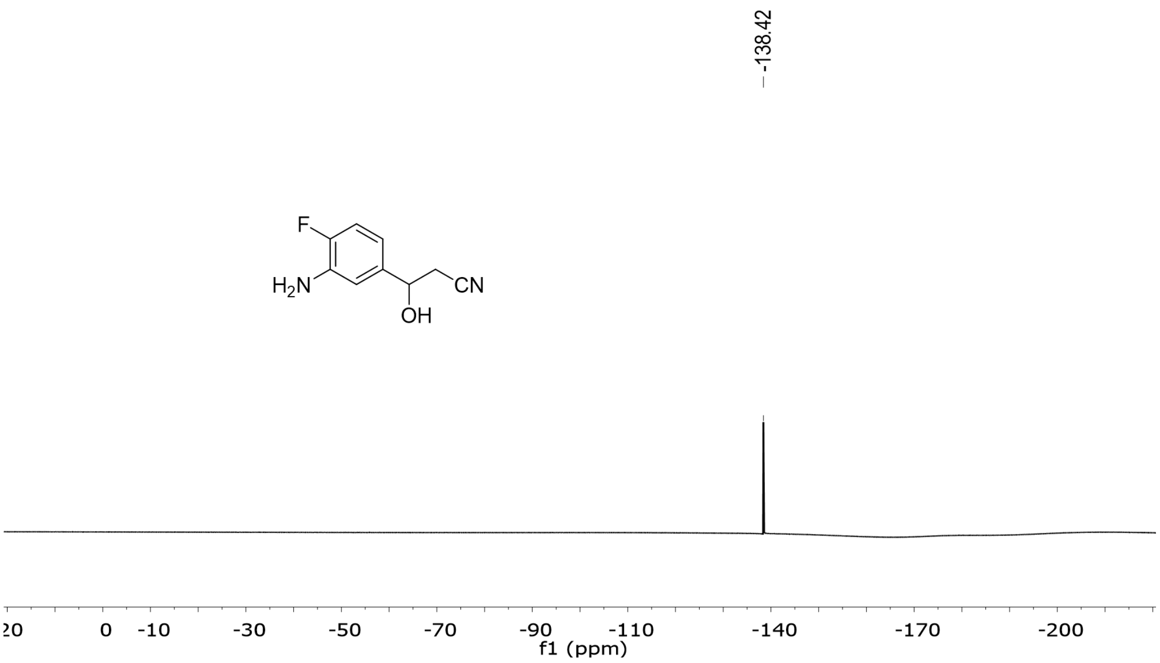

Figure S30: ^{19}F NMR spectrum of 3-(3-Amino-4-fluorophenyl)-3-hydroxypropanenitrile **(5b)** (CD_3OD, 471 MHz).

Synthetic Procedure for 3-Amino-1-(3-aminophenyl)propan-1-ol (6a)

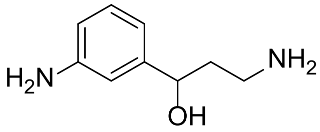

6a

6a was synthesized following general synthetic procedure (purified by flash column chromatography using ethyl acetate/methanol/ammonium hydroxide = 9/1/0.4) as pale yellow oil, yield 62%. ^{1}H NMR (500 MHz, CD_3OD) δ 7.11 (dd, *J* = 7.5 Hz, 1H), 6.78 (s, 1H), 6.73 (d, *J* = 7.5 Hz, 1H), 6.65 (d, *J* = 7.5 Hz, 1H), 4.63 (dd, *J* = 5.5 Hz, 1H), 2.77-2.68 (m, 2H), 1.95-1.79 (m, 2H). ^{13}C(^{1}H) NMR (126 MHz, CD_3OD) δ 148.8, 147.5, 130.2, 117.0, 115.6, 114.2, 73.8, 42.9, 39.8. HRMS (ESI): *m/z* calculated for $C_9H_{14}N_2O$ $[M]^+$ 166.1106; found 167.1067 $[M+H]^+$.

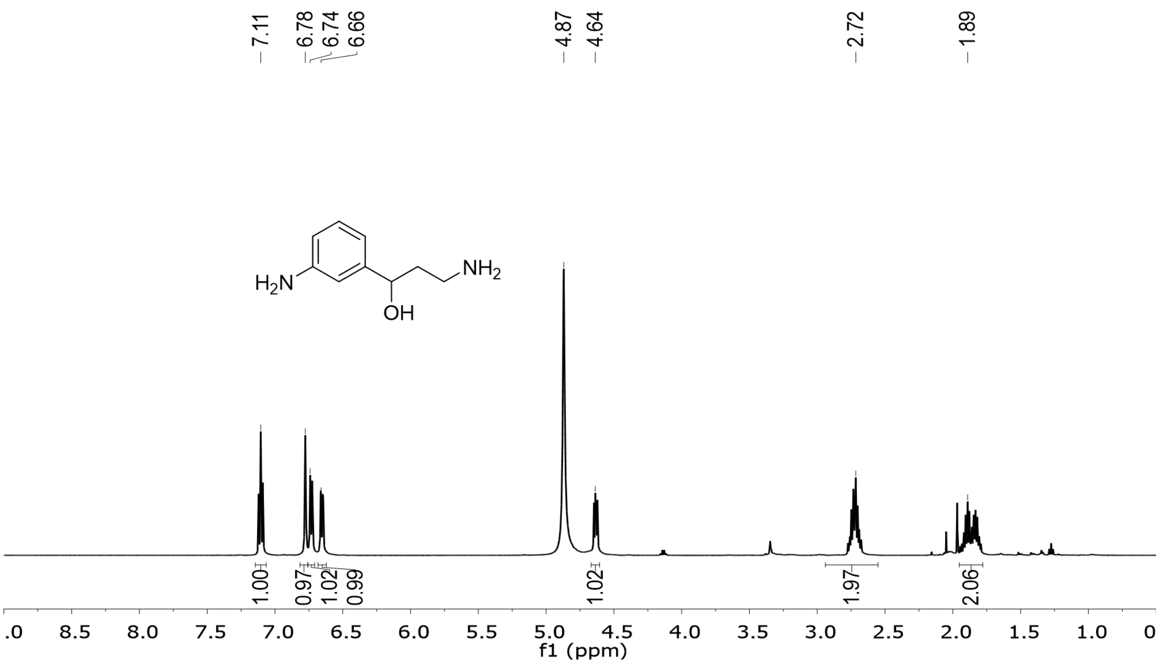

Figure S31: ^{1}H NMR spectrum of 3-Amino-1-(3-aminophenyl)propan-1-ol **(6a)** (CD_3OD, 500 MHz).

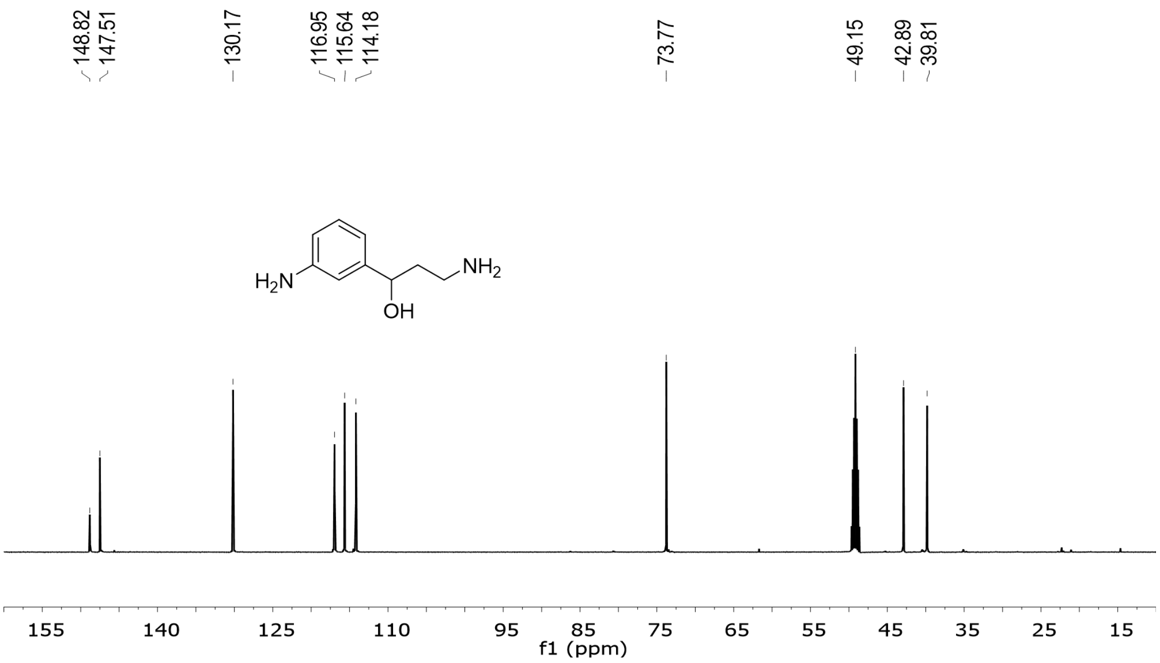

Figure S32: ^{13}C NMR spectrum of 3-Amino-1-(3-aminophenyl)propan-1-ol **(6a)** (CD_3OD, 126 MHz).

Synthetic Procedure for 3-Amino-1-(3-amino-4-fluorophenyl)propan-1-ol (6b)

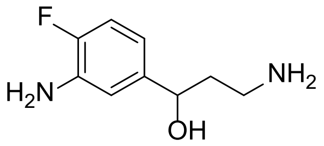

6b

6b was synthesized following general synthetic procedure (purified by flash column chromatography using ethyl acetate/methanol/ammonium hydroxide = 9/1/0.4) as pale yellow oil, yield 76%. ^{1}H NMR (500 MHz, CD_3OD) δ 6.94-6.86 (m, 2H), 6.67-6.64 (m, 1H), 4.62 (dd, J = 8.0 Hz, 5.5 Hz, 1H), 2.77-2.67 (m, 2H), 1.92-1.76 (m, 2H). $^{13}C(^{1}H)$ NMR (126 MHz, CD_3OD) δ 152.5 (d, $J_{C\text{-}F}$ = 236.88 Hz), 142.8 (d, $J_{C\text{-}F}$ = 3.78 Hz), 136.6 (d, $J_{C\text{-}F}$ = 12.60 Hz), 116.6 (d, $J_{C\text{-}F}$ = 6.30 Hz), 115.9 (d, $J_{C\text{-}F}$ = 3.78 Hz) 115.7 (d, $J_{C\text{-}F}$ = 20.16 Hz), 73.3, 42.8, 39.8. ^{19}F NMR (471 MHz, CD_3OD) δ -139.38. HRMS (ESI): *m/z* calculated for $C_9H_{13}FN_2O$ $[M]^+$ 184.1012; found 184.1012.

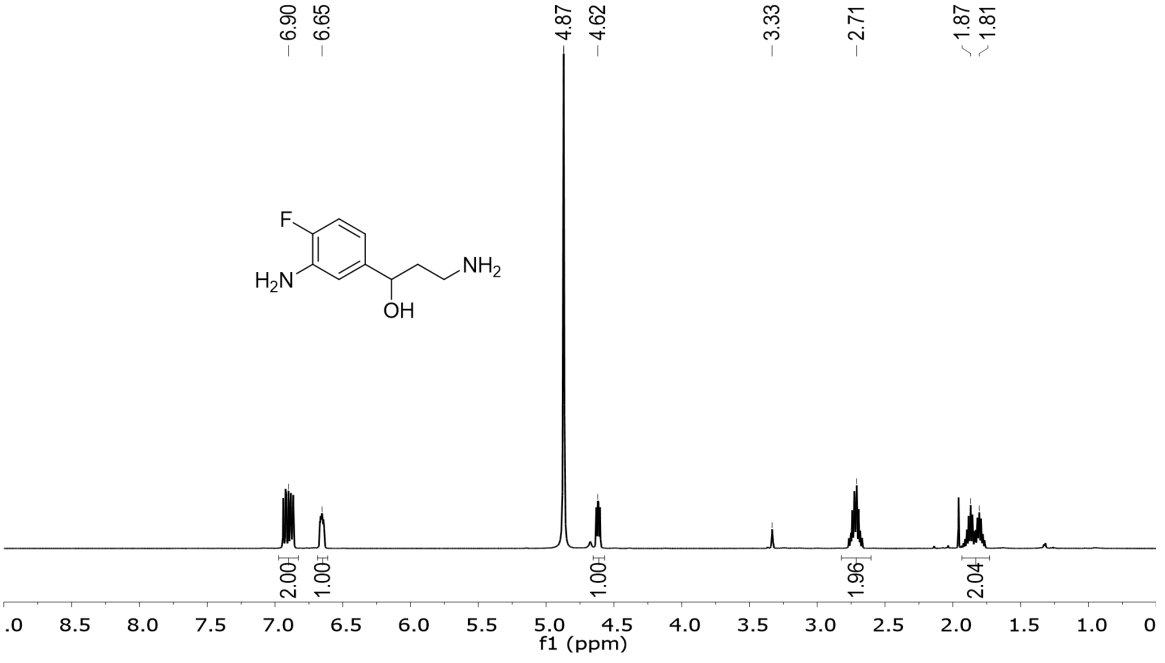

Figure S33: ^{1}H NMR spectrum of 3-Amino-1-(3-amino-4-fluorophenyl)propan-1-ol **(6b)** (CD_3OD, 500 MHz).

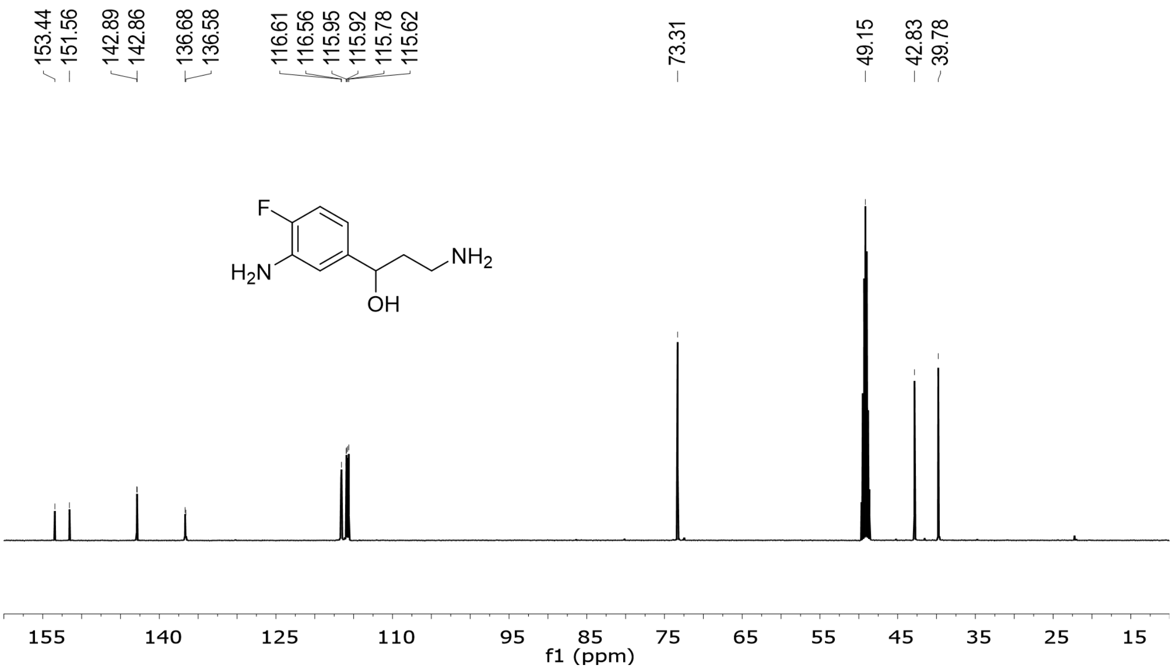

Figure S34: ^{13}C NMR spectrum of 3-Amino-1-(3-amino-4-fluorophenyl)propan-1-ol **(6b)** (CD_3OD, 126 MHz).

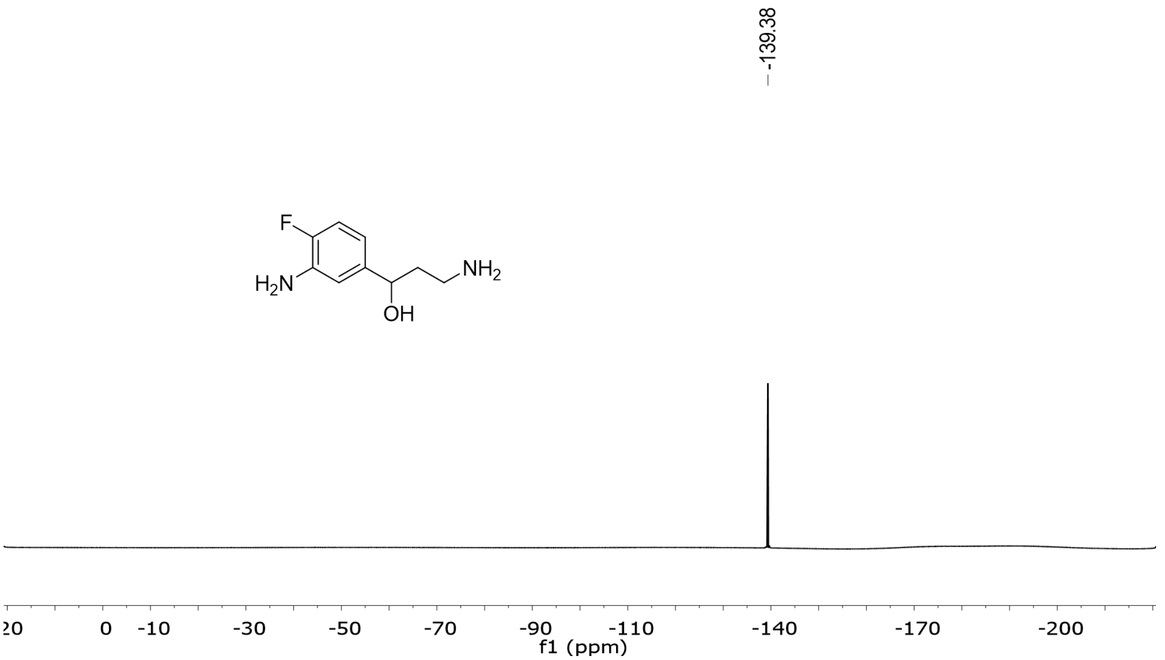

Figure S35: ^{19}F NMR spectrum of 3-Amino-1-(3-amino-4-fluorophenyl)propan-1-ol **(6b)** (CD_3OD, 471 MHz).

Synthetic Procedure for (*E*)-3-Amino-1-(3-(phenyldiazenyl)phenyl)propan-1-ol [(*E*)-7]

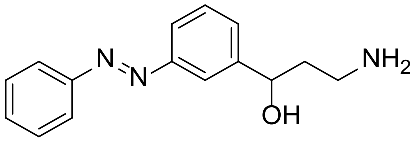

(*E*)-7 was synthesized following general synthetic procedure (purified by flash column chromatography using ethyl acetate/methanol/ammonium hydroxide = 14/1/0.4) as orange solid, yield 62%. ^{1}H NMR (500 MHz, CD_3OD) δ 7.95 (s, 1H), 7.91 (d, *J* = 7.0 Hz, 2H), 7.80 (s, 1H), 7.56-7.49 (m, 5H), 4.63 (dd, *J* = 5.5 Hz, 1H), 2.83-2.73 (m, 2H), 1,99-1.84 (m, 2H). ^{13}C(^{1}H) NMR (126 MHz, CD_3OD) δ 154.2, 154.1, 148.3, 132.4, 130.4, 130.4, 129.9, 123.9, 123.1, 121.1, 73.2, 43.0, 39.8. HRMS (ESI): *m/z* calculated for $C_{15}H_{17}N_3O$ $[M]^+$ 256.1444; found 256.1435.

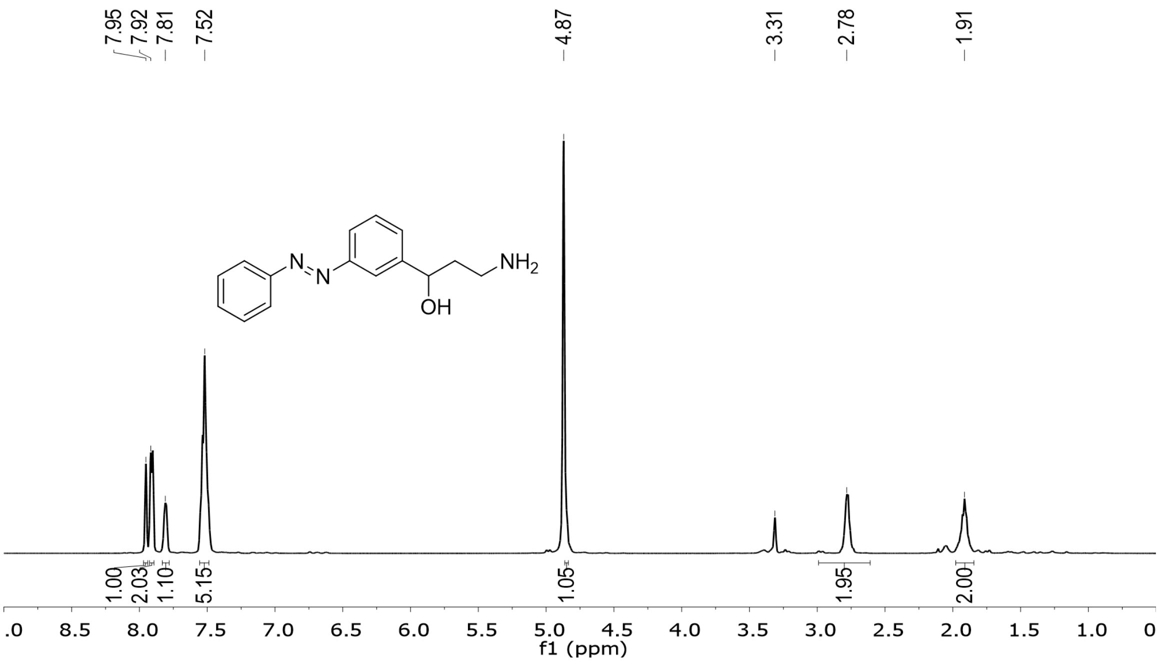

Figure S36: ^{1}H NMR spectrum of (*E*)-3-Amino-1-(3-(phenyldiazenyl)phenyl)propan-1-ol [***(E)*-7**] (CD_3OD, 500 MHz).

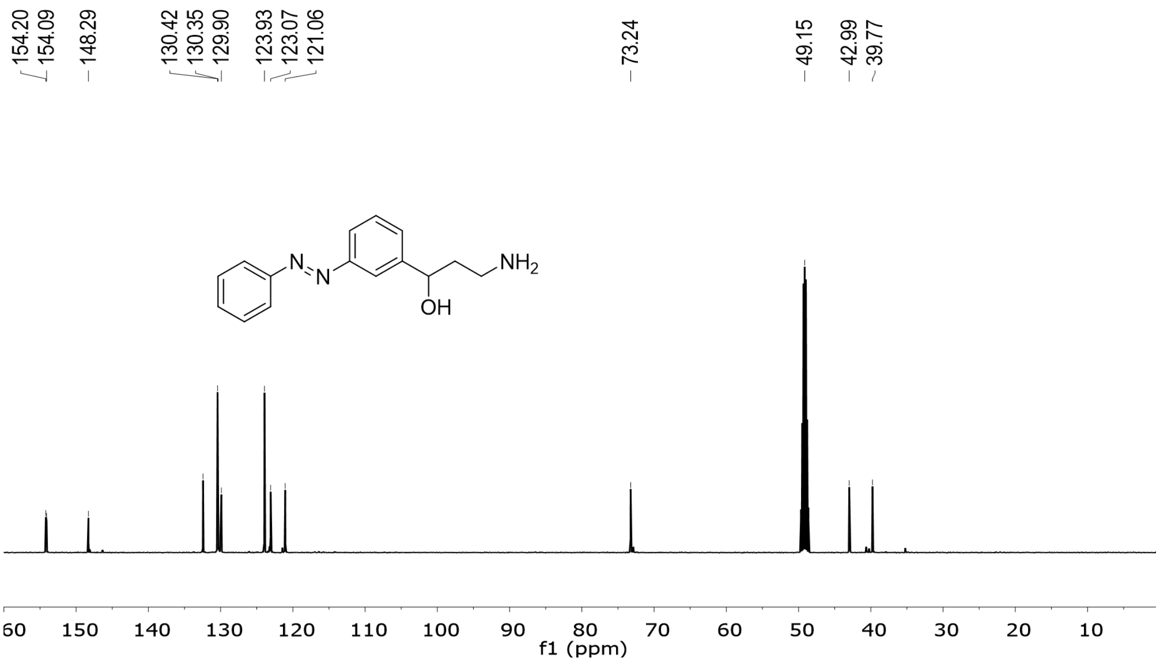

Figure S37: ^{13}C NMR spectrum of (*E*)-3-Amino-1-(3-(phenyldiazenyl)phenyl)propan-1-ol [***(E)*-7**] (CD_3OD, 126 MHz).

Synthetic Procedure for (*E*)-3-Amino-1-(3-((2,6-difluorophenyl)diazenyl)phenyl)propan-1-ol [(*E*)-8]

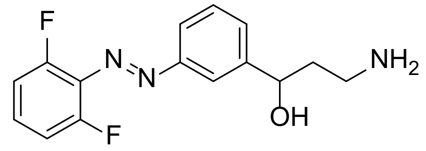

(*E*)-8 was synthesized following general synthetic procedure (purified by flash column chromatography using ethyl acetate/methanol/ammonium hydroxide = 14/1/0.4) as orange solid, yield 58%. ^{1}H NMR (500 MHz, CD_3OD) δ 7.94 (s, 1H), 7.80 (d, *J* = 7.5 Hz, 1H), 7.59-7.52 (m, 2H), 7.44 (m, 1H), 7.14 (dd, *J* = 9.0 Hz, 2H). $^{13}C(^{1}H)$ NMR (126 MHz, CD_3OD) δ 157.12 (dd, $J_{C\text{-}F}$ = 258.24 Hz, 4.40 Hz), 154.8, 148.4, 132.3 (t, $J_{C\text{-}F}$ = 10.46 Hz), 130.9, 130.5, 123.2, 121.0, 113.9 (d, $J_{C\text{-}F}$ = 4.03 Hz), 113.7 (d, $J_{C\text{-}F}$ = 4.03 Hz), 73.1, 42.1, 39.6. ^{19}F NMR (471 MHz, CD_3OD) δ -123.96. HRMS (ESI): *m/z* calculated for $C_{15}H_{15}F_2N_3O$ $[M]^+$ 291.1183; found 292.1250 $[M+H]^+$.

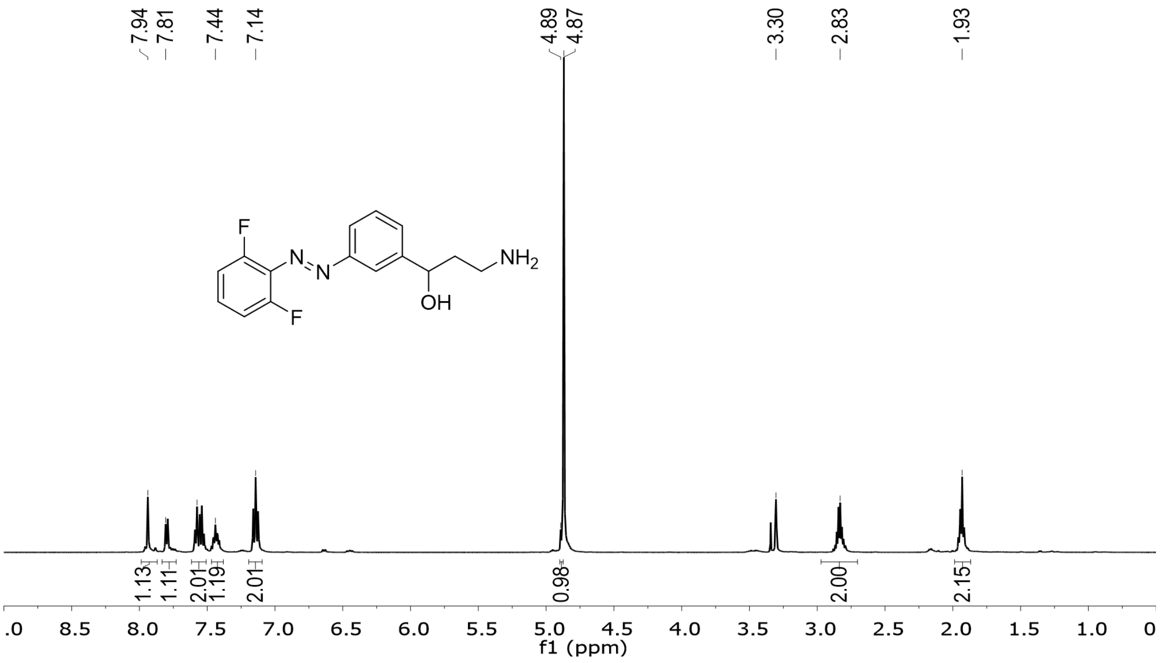

Figure S38: ^{1}H NMR spectrum of (*E*)-3-Amino-1-(3-((2,6-difluorophenyl)diazenyl)phenyl)propan-1-ol [(***E***)**-8**] (CD_3OD, 500 MHz).

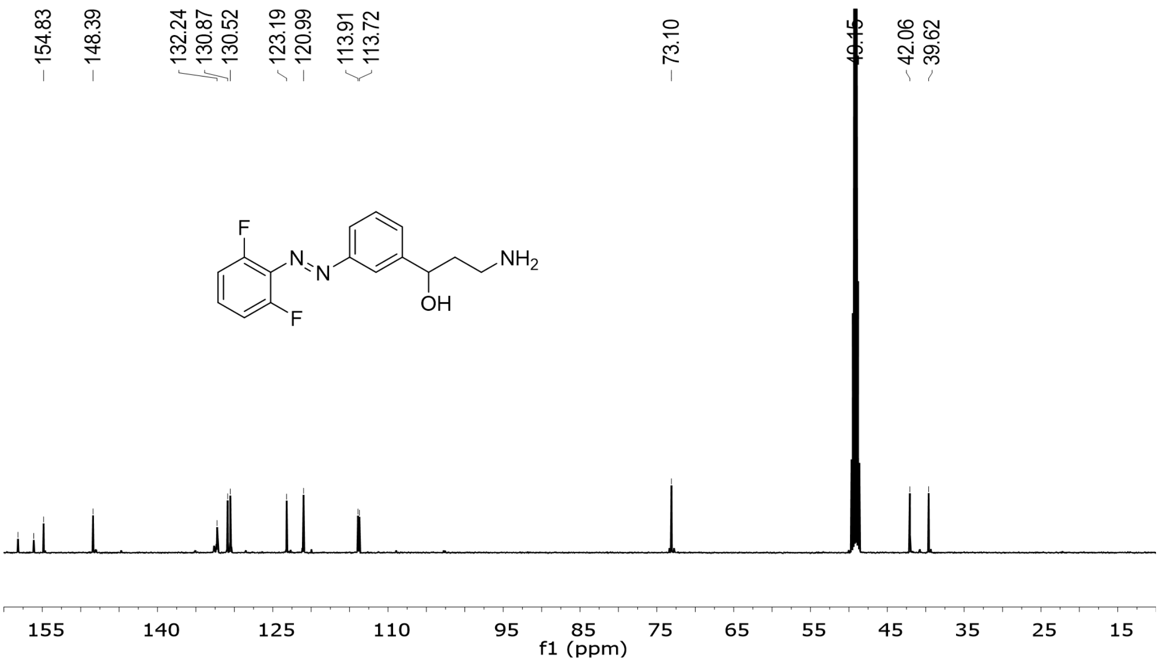

Figure S39: ^{13}C NMR spectrum of (*E*)-3-Amino-1-(3-((2,6-difluorophenyl)diazenyl)phenyl)propan-1-ol [**(*E*)-8**] (CD_3OD, 126 MHz).

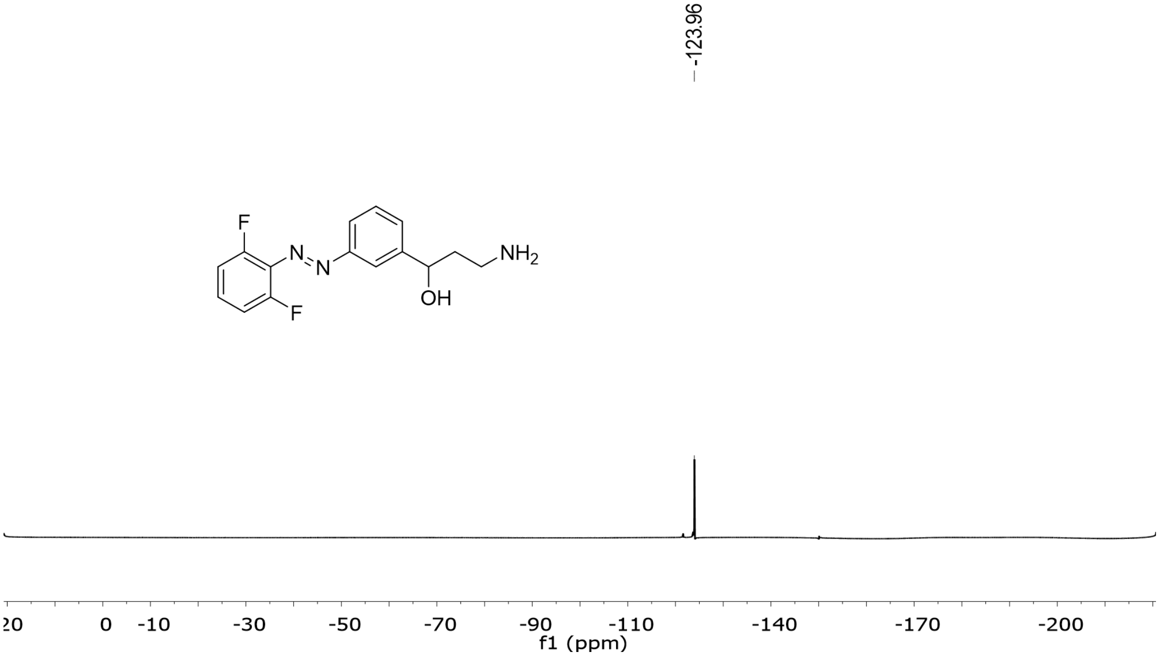

Figure S40: ^{19}F NMR spectrum of (*E*)-3-Amino-1-(3-((2,6-difluorophenyl)diazenyl)phenyl)propan-1-ol **[(*E*)-8]** (CD_3OD, 471 MHz).

Synthetic Procedure for (*E*)-3-Amino-1-(3-((2,6-difluorophenyl)diazenyl)-4-fluorophenyl)propan-1-ol [(*E*)-9]

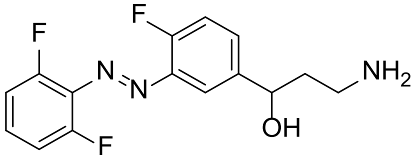

(*E*)-9 was synthesized following general synthetic procedure (purified by flash column chromatography using ethyl acetate/methanol/ammonium hydroxide = 14/1/0.4) as orange solid, yield 44%. ^{1}H NMR (500 MHz, CD_3OD) δ 7.76 (dd, *J* = 7 Hz, 2 Hz, 1H), 7.64-7.61 (m, 1H), 7.52-7.46 (m, 1H), 7.35 (dd, *J* = 10 Hz, 8.5 Hz, 1H), 7.18 (dd, *J* = 9 Hz, 2H), 4.82 (dd, *J* = 8 Hz, 5.5 Hz, 1H), 2.85-2.76 (m, 2H), 1.92-1.86 (m, 2H). ^{13}C(^{1}H) NMR (126 MHz, CD_3OD) δ 160.9 (d, $J_{C\text{-}F}$ = 258.30 Hz), 157.2 (dd, $J_{C\text{-}F}$ = 258.30 Hz, 3.78 Hz), 143.7 (d, $J_{C\text{-}F}$ = 2.52 Hz), 142.2 (d, $J_{C\text{-}F}$ = 7.56 Hz), 132.9 (d, $J_{C\text{-}F}$ = 11.34 Hz), 132.7 (d, $J_{C\text{-}F}$ = 8.82 Hz), 118.3 (d, $J_{C\text{-}F}$ = 20.16 Hz), 115.4, 114.0 (d, $J_{C\text{-}F}$ = 5.04 Hz), 113.8 (d, $J_{C\text{-}F}$ = 3.78 Hz), 72.6, 42.5, 39.6. ^{19}F NMR (471 MHz, CD_3OD) δ -123.52, -127.93. HRMS (ESI): *m/z* calculated for $C_{15}H_{13}F_3N_3O$ $[M]^+$ 309.1089; found 309.1090.

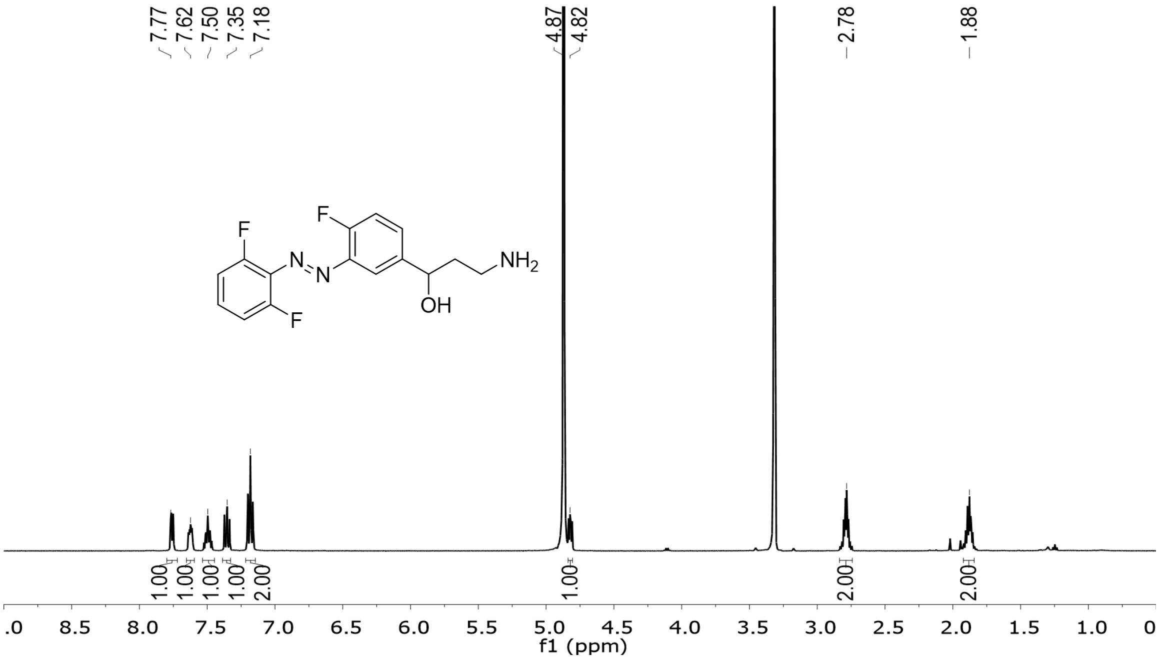

Figure S41: ^{1}H NMR spectrum of (*E*)-3-Amino-1-(3-((2,6-difluorophenyl)diazenyl)-4-fluorophenyl)propan-1-ol **[(*E*)-9]** (CD_3OD, 500 MHz).

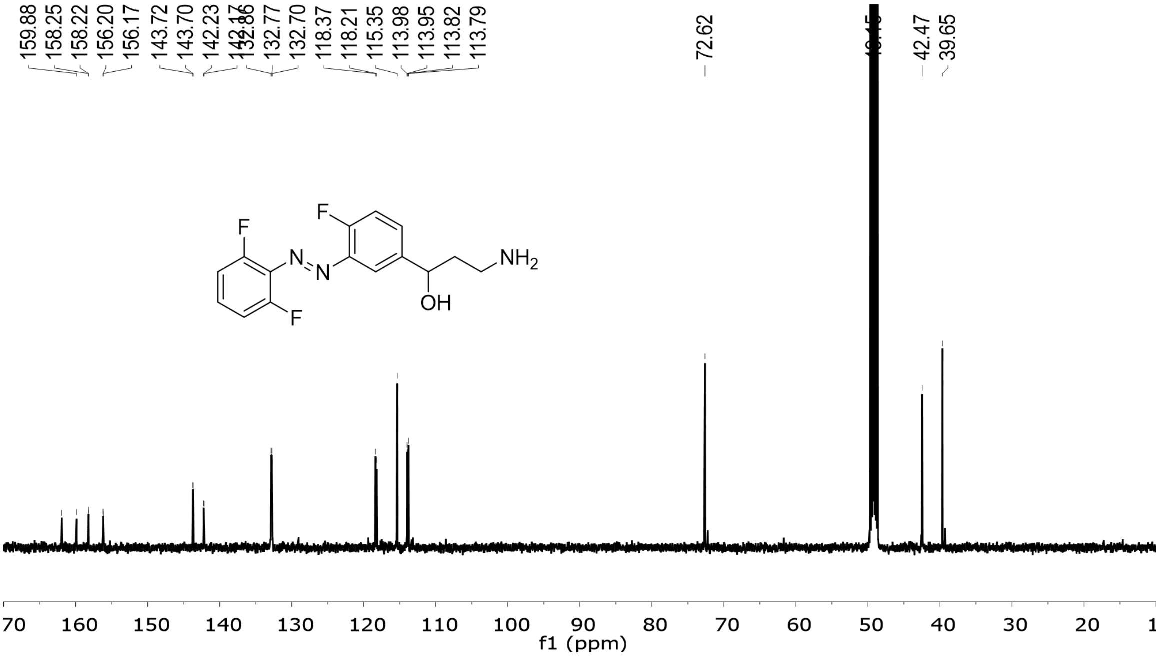

Figure S42: ^{13}C NMR spectrum of (*E*)-3-Amino-1-(3-((2,6-difluorophenyl)diazenyl)-4-fluorophenyl)propan-1-ol [***(E)*-9**] (CD_3OD, 126 MHz).

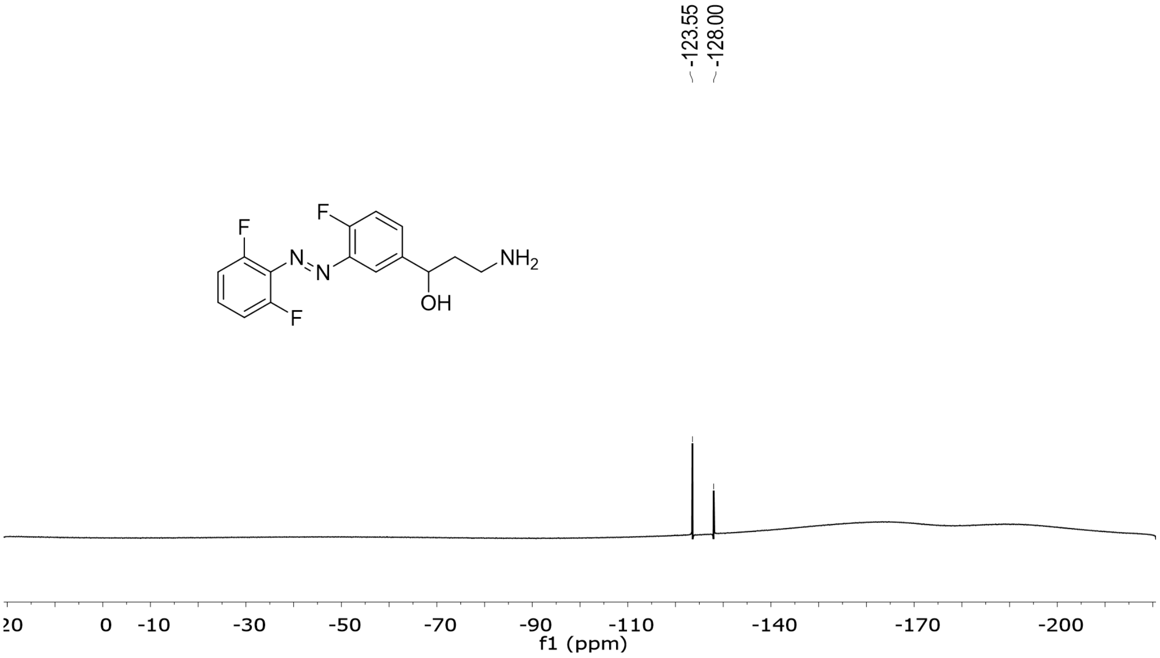

Figure S43: ^{19}F NMR spectrum of (*E*)-3-Amino-1-(3-((2,6-difluorophenyl)diazenyl)-4-fluorophenyl)propan-1-ol **[(*E*)-3F]** (CD_3OD, 471 MHz).

References

(1) Stecher, H.; Palczewski, K. Multienzyme analysis of visual cycle. *Methods Enzymol.* **2000**, *316*, 330-344. DOI: 10.1016/s0076-6879(00)16733-3 From NLM Medline.

(2) Kabsch, W. Integration, scaling, space-group assignment and post-refinement. *Acta Crystallogr. D Biol. Crystallogr.* **2010**, *66* (Pt 2), 133-144. DOI: 10.1107/S0907444909047374 From NLM Medline. Kabsch, W. Xds. *Acta Crystallogr. D Biol. Crystallogr.* **2010**, *66* (Pt 2), 125-132. DOI: 10.1107/S0907444909047337 From NLM Medline.

(3) Emsley, P.; Lohkamp, B.; Scott, W. G.; Cowtan, K. Features and development of Coot. *Acta Crystallogr. D Biol. Crystallogr.* **2010**, *66* (Pt 4), 486-501. DOI: 10.1107/S0907444910007493 From NLM Medline.

(4) Williams, C. J.; Headd, J. J.; Moriarty, N. W.; Prisant, M. G.; Videau, L. L.; Deis, L. N.; Verma, V.; Keedy, D. A.; Hintze, B. J.; Chen, V. B.; et al. MolProbity: More and better reference data for improved all-atom structure validation. *Protein Sci.* **2018**, *27* (1), 293-315. DOI: 10.1002/pro.3330 From NLM Medline.

(5) Read, R. J.; Adams, P. D.; Arendall, W. B., 3rd; Brunger, A. T.; Emsley, P.; Joosten, R. P.; Kleywegt, G. J.; Krissinel, E. B.; Lutteke, T.; Otwinowski, Z.; et al. A new generation of crystallographic validation tools for the protein data bank. *Structure* **2011**, *19* (10), 1395-1412. DOI: 10.1016/j.str.2011.08.006 From NLM Medline.

(6) O'Boyle, N. M.; Banck, M.; James, C. A.; Morley, C.; Vandermeersch, T.; Hutchison, G. R. Open Babel: An open chemical toolbox. *J Cheminform* **2011**, *3*, 33. DOI: 10.1186/1758-2946-3-33 From NLM PubMed-not-MEDLINE.

(7) Bannwarth, C.; Caldeweyher, E.; Ehlert, S.; Hansen, A.; Pracht, P.; Seibert, J.; Spicher, S.; Grimme, S. Extended tight-binding quantum chemistry methods. *WIREs Computational Molecular Science* **2020**, *11* (2). DOI: 10.1002/wcms.1493.

(8) Pracht, P.; Grimme, S.; Bannwarth, C.; Bohle, F.; Ehlert, S.; Feldmann, G.; Gorges, J.; Muller, M.; Neudecker, T.; Plett, C.; et al. CREST-A program for the exploration of low-energy molecular chemical space. *J Chem Phys* **2024**, *160* (11). DOI: 10.1063/5.0197592 From NLM PubMed-not-MEDLINE.

(9) Neese, F. The ORCA program system. *WIREs Computational Molecular Science* **2011**, *2* (1), 73-78. DOI: 10.1002/wcms.81.

(10) Shao, Y.; Mei, Y.; Sundholm, D.; Kaila, V. R. I. Benchmarking the Performance of Time-Dependent Density Functional Theory Methods on Biochromophores. *J Chem Theory Comput* **2020**, *16* (1), 587-600. DOI: 10.1021/acs.jctc.9b00823 From NLM Medline. Liang, J.; Feng, X.; Hait, D.; Head-Gordon, M. Revisiting the Performance of Time-Dependent Density Functional Theory for Electronic Excitations: Assessment of 43 Popular and Recently Developed Functionals from Rungs One to Four. *J Chem Theory*

Comput **2022**, *18* (6), 3460-3473. DOI: 10.1021/acs.jctc.2c00160 From NLM PubMed-not-MEDLINE. Takeshita, T. DFT and TD-DFT Study on Azobenzene-Based Dye Covalently Attached to Silane Coupling Agents: Toward Dye-Sensitized TiO2 Catalyst and Dye-Sensitized Solar Cell Applications. *ChemistrySelect* **2021**, *6* (24), 6011-6018. DOI: 10.1002/slct.202101495. Jacquemin, D.; Perpète, E. A.; Scuseria, G. E.; Ciofini, I.; Adamo, C. Extensive TD-DFT investigation of the first electronic transition in substituted azobenzenes. *Chemical Physics Letters* **2008**, *465* (4-6), 226-229. DOI: 10.1016/j.cplett.2008.09.071.

(11) Bassetto, M.; Kolesnikov, A. V.; Lewandowski, D.; Kiser, J. Z.; Halabi, M.; Einstein, D. E.; Choi, E. H.; Palczewski, K.; Kefalov, V. J.; Kiser, P. D. Dominant role for pigment epithelial CRALBP in supplying visual chromophore to photoreceptors. *Cell Rep.* **2024**, *43* (5), 114143. DOI: 10.1016/j.celrep.2024.114143 From NLM Publisher.

(12) Claire Deo, N. B., Rémi Métivier, Pascal Retailleau, Juan Xie. Photoswitchable Arene Ruthenium Complexes Containing o-Sulfonamide Azobenzene Ligands. *Organometallics* **2015**, *34* (24), 5775-5784.

(13) Bowen Li, Y. H., Gregory P. Tochtrop. An Acid-Controlled Method for the Regioselective Functionalization of Anilines over Aliphatic Amines. *European Journal of Chemistry* **2023**, *29* (59). DOI: https://doi.org/10.1002/chem.202301336.

(14) Hong Ji Li, L. W. Triethanolamine as an Efficient and Reusable Base, Ligand and Reaction Medium for Phosphane-Free Palladium-Catalyzed Heck Reactions. *European Journal of Chemistry* **2006**, *2006* (22), 5099-5102. DOI: 10.1002/ejoc.200600561.

(15) Saranya, S., Chandiran, S., Gopalan, G., Jijitha, V., Kv, R. A Facile Access to trans-3-Styryl-4-hydrazinocyclopentenes via Palladium--Catalyzed Ring Opening of Diazanorbornenes with (Z)-β-Bromostyrenes/2,3-Dibromohydrocinnamic Acids. *Synthesis* **2017**, *50* (1). DOI: 10.1055/s-0036-1589108.

Detailed Description of Experimental Contributions

Design, synthesis, purification, and analytical characterization of stilbene emixustat analogs (*E*)-3 and (*Z*)-3 (Schemes S1–S2, Fig. 1B,C): **B.L., J.Za.**

Design, synthesis, purification, and analytical characterization of azobenzene emixustat analogs 7–9 and intermediates (including development of successful synthetic route in Scheme 1) (Schemes S1–S2, Scheme 1, Fig. 1D,F): **B.L., Y.H., J.Za., L.M.B.,**

In vitro RPE65 inhibition assays with emixustat and stilbene analogs (*E*)-3 and (*Z*)-3 to determine IC_{50} values (Fig. 1C): **B.L., J.Zh., J.Za.,**

In vitro RPE65 inhibition assays with emixustat and azobenzene analogs (*E*)-7 and (*Z*)-7 to determine IC_{50} values (Fig. 1G): **B.L., J.Zh., J.Za.,**

UV–Vis spectroscopic characterization of azobenzene analog (*E*)-7 and (*Z*)-7, including identification of π→π and n→π bands** (Fig. 1E): **B.L.**

Determination of photostationary states (PSS) of azobenzene emixustat derivatives (*E*)-7, (*E*)-8, and (*E*)-9 under different excitation wavelengths (254–460 nm) via HPLC (Table 1): **B.L.**

Thermal *Z*→*E* isomerization kinetics of (*Z*)-7, (*Z*)-8, and (*Z*)-9 in PBS/10% BSA at 37 °C (and related stability experiments in DMSO and PBS/10% FBS) (Fig. 2A, Fig. S2): **B.L.**

UV–Vis spectroscopy of (*E*)-9 and (*Z*)-9, including analysis of separated n→π bands induced by ortho-fluorination* (Fig. 2B): **B.L.**

Photoisomerization kinetics of (*Z*)-9 under visible white light at different intensities (500–5000 lux) with determination of time constants to PSS by HPLC (Fig. 2C): **B.L.**

In vitro RPE65 inhibition assays comparing emixustat, (*E*)-9, (*Z*)-9, and (*Z*)-9 under 395/405 nm illumination to quantify light-driven potency shift (Fig. 2D): **B.L., G.P.T.**

Purification, crystallization, and X-ray structure determination of bovine RPE65 in complex with (*E*)-9, including electron density analysis and comparison to emixustat/MB-001 complexes (Fig. 3A–D, Table S2): **M.B., P.D.K.,**

Quantum-chemical (TDDFT) calculations of UV–Vis spectra for (*E*)-9 and (*Z*)-9 in solution, including assignment of π→π and n→π transitions** (Fig. 3E, Fig. S1): **P.M.W.**

TDDFT calculations for the RPE65-bound conformation of (*E*)-9 to model suppression of the n→π band and impact of azo twisting on photophysics* (Fig. 3F): **P.M.W.**

In vivo scotopic ERG measurements to assess RPE65 inhibition and visual cycle suppression after photobleach and IP administration of vehicle, emixustat, (*E*)-9, or (*Z*)-9 at multiple doses (Fig. 4A–C): **M.B.**

Whole-eye retinoid extraction and normal-phase HPLC quantification of 11-cis-retinal and all-trans-retinyl esters following photobleach and inhibitor treatment (Fig. 4D–F): **M.B., G.P.T.**

LC–MS/MS method development for azo-emixustat derivatives and stilbene internal standards, including optimization of precursor/product ion MRM transitions (Fig. S6): **M.B., X.C., G.P.T.**

In vivo LC–MS/MS quantification of (*E*)-9/(*Z*)-9 ratios in whole-eye homogenates after systemic administration of (*Z*)-9 with or without 405 nm or 630 nm illumination (Fig. 5A,B): **M.B., X.C., G.P.T.**

Functional assessment of light-triggered visual cycle suppression: pre- vs post-405 nm illumination dosing with (*E*)-9 or (*Z*)-9, followed by dark adaptation and HPLC measurement of 11-cis-retinal regeneration (Fig. 5C,D): **M.B.**

Spectral characterization (emission spectra) of white LED photobleach sources, violet/red LEDs, and vivarium fluorescent lighting used in the in vivo experiments (Fig. S3): **M.B.**

In vivo scotopic ERG comparison of dark adaptation after photobleach with white vs violet LED light (Fig. S4): **M.B.**

Oral dosing pharmacodynamic studies: scotopic ERG time course after single oral administration of emixustat or (*E*)-9 following photobleach to assess duration of visual cycle suppression (Fig. S5A,B): **M.B.**

Execution of retinal light-damage (photic retinopathy) protocol: 8 h, 15,000 lux white LED exposure after IP dosing with vehicle, emixustat, or (*E*)-9, including animal handling and dosing schedule (underlies Fig. 6A–H): **M.B.**

In vivo scanning laser ophthalmoscopy (SLO) imaging and analysis of retinal autofluorescent puncta one week after light damage and treatment (Fig. 6A): **M.B.**

Optical coherence tomography (OCT) imaging and quantification of outer nuclear layer thickness at defined distances from the optic nerve head after light damage and treatment (Fig. 6B,C): **M.B.**

Histological processing (fixation, embedding, H&E staining) of retinal sections and quantification of ONL thickness / photoreceptor nuclei counts along the dorsal–ventral axis (Fig. 6D,E): **M.B.**

Dark adapted ERG intensity–response measurements (0.002–100 cd·s/m²) one week after light damage to assess preservation of retinal function by emixustat and (*E*)-9 (Fig. 6F–H): **M.B.**
